# Genomic diversity affects the accuracy of bacterial single-nucleotide polymorphism–calling pipelines

**DOI:** 10.1093/gigascience/giaa007

**Published:** 2020-02-06

**Authors:** Stephen J Bush, Dona Foster, David W Eyre, Emily L Clark, Nicola De Maio, Liam P Shaw, Nicole Stoesser, Tim E A Peto, Derrick W Crook, A Sarah Walker

**Affiliations:** 1 Nuffield Department of Medicine, University of Oxford, John Radcliffe Hospital, Headington, Oxford, OX3 9DU, UK; 2 National Institute for Health Research Health Research Protection Unit in Healthcare Associated Infections and Antimicrobial Resistance at University of Oxford in partnership with Public Health England, Oxford, John Radcliffe Hospital, Headington, Oxford, OX3 9DU, UK; 3 National Institute for Health Research Oxford Biomedical Research Centre, Oxford, John Radcliffe Hospital, Headington, Oxford, OX3 9DU, UK; 4 The Roslin Institute and Royal (Dick) School of Veterinary Studies, University of Edinburgh, Easter Bush Campus, Midlothian, EH25 9RG, UK; 5 European Molecular Biology Laboratory, European Bioinformatics Institute (EMBL-EBI), Wellcome Genome Campus, Hinxton, Cambridgeshire, CB10 1SH, UK

**Keywords:** SNP calling, variant calling, evaluation, benchmarking, bacteria

## Abstract

**Background:**

Accurately identifying single-nucleotide polymorphisms (SNPs) from bacterial sequencing data is an essential requirement for using genomics to track transmission and predict important phenotypes such as antimicrobial resistance. However, most previous performance evaluations of SNP calling have been restricted to eukaryotic (human) data. Additionally, bacterial SNP calling requires choosing an appropriate reference genome to align reads to, which, together with the bioinformatic pipeline, affects the accuracy and completeness of a set of SNP calls obtained. This study evaluates the performance of 209 SNP-calling pipelines using a combination of simulated data from 254 strains of 10 clinically common bacteria and real data from environmentally sourced and genomically diverse isolates within the genera *Citrobacter, Enterobacter*, *Escherichia*, and *Klebsiella*.

**Results:**

We evaluated the performance of 209 SNP-calling pipelines, aligning reads to genomes of the same or a divergent strain. Irrespective of pipeline, a principal determinant of reliable SNP calling was reference genome selection. Across multiple taxa, there was a strong inverse relationship between pipeline sensitivity and precision, and the Mash distance (a proxy for average nucleotide divergence) between reads and reference genome. The effect was especially pronounced for diverse, recombinogenic bacteria such as *Escherichia coli* but less dominant for clonal species such as *Mycobacterium tuberculosis*.

**Conclusions:**

The accuracy of SNP calling for a given species is compromised by increasing intra-species diversity. When reads were aligned to the same genome from which they were sequenced, among the highest-performing pipelines was Novoalign/GATK. By contrast, when reads were aligned to particularly divergent genomes, the highest-performing pipelines often used the aligners NextGenMap or SMALT, and/or the variant callers LoFreq, mpileup, or Strelka.

## Introduction

Accurately identifying single-nucleotide polymorphisms (SNPs) from bacterial DNA is essential for monitoring outbreaks (as in [1, 2]) and predicting phenotypes, such as antimicrobial resistance [3], although the pipeline selected for this task strongly affects the outcome [4]. Current bacterial sequencing technologies generate short fragments of DNA sequence (“reads”) from which the bacterial genome can be reconstructed. Reference-based mapping approaches use a known reference genome to guide this process, using a combination of an aligner, which identifies the location in the genome from which each read is likely to have arisen, and a variant caller, which summarizes the available information at each site to identify variants including SNPs and indels (see reviews for an overview of alignment [5, 6] and SNP calling [7] algorithms). This evaluation focuses only on SNP calling; we did not evaluate indel calling because this can require different algorithms (see review [8]).

The output from different aligner/caller combinations is often poorly concordant. For example, up to 5% of SNPs are uniquely called by 1 of 5 different pipelines [9] with even lower agreement on structural variants [10].

Although a mature field, systematic evaluations of variant-calling pipelines are often limited to eukaryotic data, usually human [11–15] but also *Caenorhabditis elegans* [16] and dairy cattle [17] (see also review [7]). This is because truth sets of known variants, such as the Illumina Platinum Genomes [18], are relatively few in number and human-centred, being expensive to create and biased toward the methods that produced them [19]. As such, to date, bacterial SNP calling evaluations are comparatively limited in scope (e.g., comparing 4 aligners with 1 caller, mpileup [20], using *Listeria monocytogenes* [21]).

Relatively few truth sets exist for bacteria, so the choice of pipeline for bacterial SNP calling is often informed by performance on human data. Many evaluations conclude in favour of the publicly available BWA-mem [22] or commercial Novoalign [23] as choices of aligner, and GATK [24, 25] or mpileup as variant callers, with recommendations for a default choice of pipeline, independent of specific analytic requirements, including Novoalign followed by GATK [15], and BWA-mem followed by either mpileup [14], GATK [12], or VarDict [11].

This study evaluates a range of SNP-calling pipelines across multiple bacterial species, both when reads are sequenced from and aligned to the same genome, and when reads are aligned to a representative genome of that species.

SNP-calling pipelines are typically constructed around a read aligner (which takes FASTQ as input and produces BAM as output) and a variant caller (which takes BAM as input and produces VCF as output), often with several pre- and post-processing steps (e.g., cleaning a raw FASTQ prior to alignment, or filtering a BAM prior to variant calling). For the purpose of this study, when evaluating the 2 core components of aligner and caller, we use “pipeline” to mean “an aligner/caller combination, with all other steps in common.”

To cover a broad range of methodologies (see review for an overview of the different algorithmic approaches [26]), we assessed the combination of 16 short-read aligners (BBMap [27], Bowtie2 [28], BWA-mem and BWA-sw [22], Cushaw3 [29], GASSST [30], GEM [31], HISAT2 [32], minimap2 [33], MOSAIK [34], NextGenMap [35], Novoalign, SMALT [36], SNAP [37], and Stampy [38] [both with and without pre-alignment with BWA-aln], and Yara [39]) used in conjunction with 14 variant callers (16GT [40], DeepVariant [41], Freebayes [42], GATK HaplotypeCaller [24, 25], LoFreq [43], mpileup [20], Octopus [44], Pilon [45], Platypus [46], SolSNP [47], SNVer [48], SNVSniffer [49], Strelka [50], and VarScan [51]). We also evaluated 3 “all-in-one” variant -alling pipelines, Snippy [52], SPANDx [53], and SpeedSeq [54], which consolidate various open-source packages into 1 tool. Reasons for excluding other programs are detailed in Supplementary Text 1. Where possible, we applied a common set of pre- or post-processing steps to each aligner/caller combination, although note that these could differ from those applied within an “all-in-one” tool (discussed further in Supplementary Text 1).

Benchmarking evaluations are, however comprehensive, ephemeral. As programs are being constantly created and updated, it will always be possible to expand the scope of any evaluation. To that end, this study originally assessed an initial subset of 41 pipelines, the combination of 4 aligners (BWA-mem, minimap2, Novoalign, and Stampy) and 10 variant callers (the aforementioned list, excluding DeepVariant, Octopus, Pilon, and SolSNP), plus Snippy.

To evaluate each of this initial set of 41 pipelines, we simulated 3 sets of 150 bp and 3 sets of 300 bp reads (characteristic of the Illumina NextSeq and MiSeq platforms, respectively) at 50-fold depth from 254 strains of 10 clinically common species (2–36 strains per species), each with fully sequenced (closed) core genomes: the gram-positive *Clostridioides difficile* (formerly *Clostridium difficile* [55]), *Listeria monocytogenes, Staphylococcus aureus*, and *Streptococcus pneumoniae* (all gram-positive)*, Escherichia coli*, *Klebsiella pneumoniae, Neisseria gonorrhoeae, Salmonella enterica*, and *Shigella dysenteriae* (all gram-negative), and *Mycobacterium tuberculosis*. For each strain, we evaluated all pipelines using 2 different genomes for alignment: one being the same genome from which the reads were simulated, and one being the NCBI “reference genome,” a high-quality (but essentially arbitrary) representative of that species, typically chosen on the basis of assembly and annotation quality, available experimental support, and/or wide recognition as a community standard (such as *C. difficile* 630, the first sequenced strain for that species [56]). We added ∼8,000–25,000 SNPs *in silico* to each genome, equivalent to 5 SNPs per genic region, or 1 SNP per 60–120 bases.

While simulation studies can offer useful insight, they can be sensitive to the specific details of the simulations. Therefore, we also evaluated performance on real data to verify our conclusions. We used 16 environmentally sourced and genomically diverse gram-negative species of the genera *Citrobacter, Enterobacter*, *Escherichia*, and *Klebsiella*, along with 2 reference strains, from which closed hybrid *de novo* assemblies were previously generated using both Illumina (short) and ONT (long; Oxford Nanopore Technologies) reads [57]. For this aspect of the study, we quintupled the scope of the evaluation from the initial set of 41 pipelines and also present results for a larger set of 209 pipelines.

All pipelines aim to call variants with high specificity (i.e., a high proportion of non-variant sites in the truth set are correctly identified as the reference allele by the pipeline) and high sensitivity (i.e., a high proportion of true SNPs are found by the pipeline). The optimal trade-off between these 2 properties may vary depending on the application. For example, in transmission inference, minimizing false-positive SNP calls (i.e., high specificity) is likely to be most important, whereas high sensitivity may be more important when identifying variants associated with antibiotic resistance. We therefore report detailed performance metrics for all pipelines, including recall (sensitivity), precision (positive predictive value, the proportion of SNPs identified that are true SNPs), and the F-score, the harmonic mean of precision and recall [11].

## Results

### Evaluating SNP-calling pipelines when the genome for alignment is also the source of the reads

**Figure 1:**
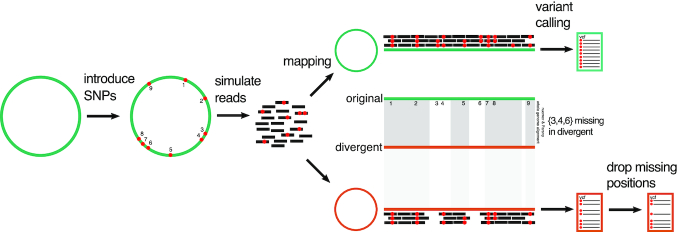
Overview of SNP-calling evaluation. SNPs were introduced *in silico* into 254 closed bacterial genomes (Supplementary Table 2) using Simulome. Reads were then simulated from these genomes. A total of 41 SNP-calling pipelines (Supplementary Table 1) were evaluated using 2 different genomes for read alignment: the original genome from which the reads were simulated and a divergent genome, the species-representative NCBI “reference genome.” In the latter case, it will not be possible to recover all of the original *in silico* SNPs because some will be found only within genes unique to the original genome. Accordingly, to evaluate SNP calls, the coordinates of the original genome need to be converted to those of the representative genome. To do so, whole-genome alignments were made using both nucmer and Parsnp, with consensus calls identified within 1-to-1 alignment blocks. Inter-strain SNPs (those not introduced *in silico*) are excluded. The remaining subset of *in silico* calls comprise the truth set for evaluation. There is a strong correlation between the total number of SNPs introduced *in silico* into the original genome and the total number of nucmer/Parsnp consensus SNPs in the divergent genome (Supplementary Figure 3).

The performance of 41 SNP-calling pipelines (Supplementary Table 1) was first evaluated using reads simulated from 254 closed bacterial genomes (Supplementary Table 2), as illustrated in Fig. 1. In order to exclude biases introduced during other parts of the workflow, such as DNA library preparation and sequencing error, reads were simulated error-free. There was negligible difference in performance when reads were simulated with sequencing errors (see Supplementary Text 1).

**Figure 2:**
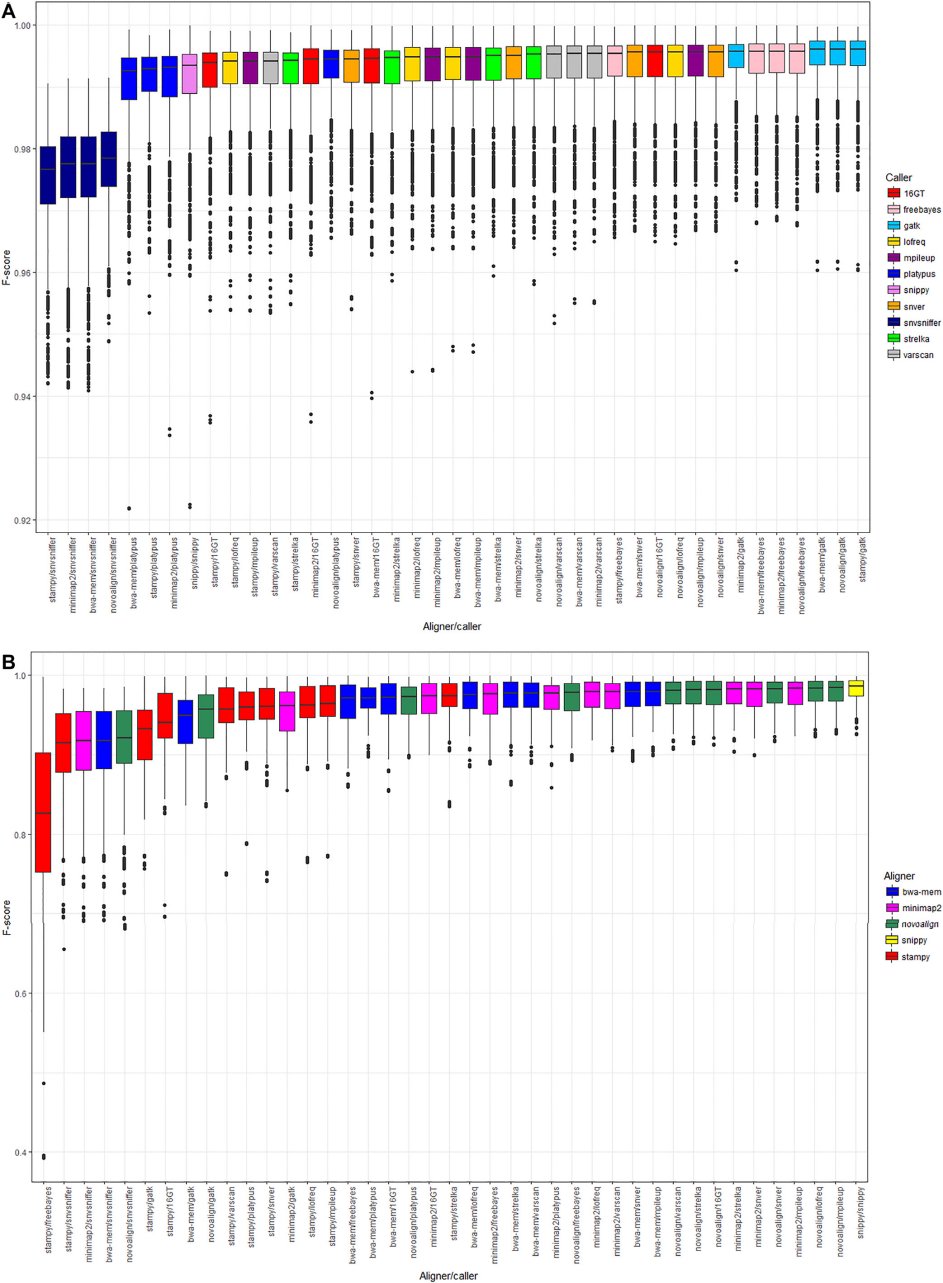
Median F-score per pipeline when the reference genome for alignment is (A) the same as the source of the reads and (B) a representative genome for that species. Panels show the median F-score of 41 different pipelines when SNPs are called using error-free 150- and 300-bp reads simulated from 254 genomes (of 10 species) at 50-fold coverage. Boxes represent the interquartile range of F-score, with midlines representing the median. Upper and lower whiskers extend, respectively, to the largest and smallest values no further than 1.5x the interquartile range. Data beyond the ends of each whisker are outliers and plotted individually. Pipelines are ordered according to median F-score and coloured according to either the variant caller (A) or aligner (B) in each pipeline. Note that because F-scores are uniformly >0.9 when the reference genome for alignment is the same as the source of the reads, the vertical axes on each panel have different scales. Genomes are detailed in Supplementary Table 2, summary statistics for each pipeline in Supplementary Tables 3 and 6, and performance ranks in Supplementary Tables 4 and 7, for alignments to the same or to a representative genome, respectively.

This dataset contains 62,484 VCFs (comprising 2 read lengths [150 and 300 bp] * 3 replicates * 254 genomes * 41 pipelines). The number of reads simulated from each species and the performance statistics for each pipeline—the number of true positives (TP), false positives (FP), and false negatives (FN), precision, recall, F-score, and total number of errors (i.e., FP + FN) per million sequenced bases—are given in Supplementary Table 3, with the distribution of F-scores illustrated in Fig. 2A.

Median F-scores were >0.99 for all but 4 aligner/callers, with small interquartile ranges (∼0.005), although outliers were nevertheless notable (Fig. 2A), suggesting that reference genome can affect performance of a given pipeline.

Table 1 shows the top-ranked pipelines averaged across all species’ genomes, based on 7 different performance measures and on the sum of their ranks (which constitutes an “overall performance” measure, lower values indicating higher overall performance). Supplementary Table 4 shows the sum of ranks for each pipeline per species, with several variant callers consistently found among the highest-performing (Freebayes and GATK) and lowest-performing pipelines (16GT and SNVSniffer), irrespective of aligner.

**Table 1:** Summary of pipeline performance across all species' genomes

	Top-ranked pipeline(s)		
Performance measure	When the reference genome is the same as the source of the reads	When the reference genome is divergent from the reads	Averaged across all simulations
F-score	bwa-mem with freebayes/gatk, minimap2 with freebayes/gatk, novoalign/gatk, stampy/gatk (0.994)	snippy (0.982)*	novoalign with lofreq/mpileup, snippy (0.986)
Precision (specificity)	snippy, bwa-mem/minimap2/novoalign/stampy with 16GT/freebayes/gatk/lofreq/mpileup/platypus/snver/strelka/varscan (1)	novoalign/snvsniffer (0.971)	novoalign/snvsniffer (0.986)
Recall (sensitivity)	bwa-mem/novoalign/stampy with gatk (0.989)	bwa-mem with 16GT/freebayes, stampy/freebayes (0.997)	bwa-mem/minimap2/stampy with freebayes (0.992)
No. of TP calls	novoalign/gatk (15,777)	bwa-mem/freebayes (13,829)	bwa-mem/freebayes (14,791)
No. of FP calls	stampy with mpileup/platypus (0)	novoalign/snvsniffer (1.825)	novoalign/snvsniffer (0.913)
No. of FN calls	novoalign/gatk (0.941)	bwa-mem/freebayes (0.188)	bwa-mem/freebayes (0.641)
Total no. of errors (FP + FN calls) per million sequenced bases	novoalign/gatk (0.944)	snippy (2.627)*	snippy (2.125)
Sum of ranks for all previous measures	novoalign/gatk (10)	snippy (20)*	novoalign/mpileup (42)

Numbers in parentheses refer to the median value, across all simulations, for each performance measure.

* Snippy is based on a BWA-mem/freebayes pipeline, although under default parameters shows improved performance. When the reference genome diverges from the reads and compared to the rank 1 position of Snippy, BWA-mem/freebayes has a median F-score of 0.965 (ranking 12 out of 41 pipelines), a median number of errors per million sequenced bases of 5.265 (ranking 26 out of 41 pipelines), and a sum of ranks of 98. FN: false negative; FP: false positive; TP: true positive.

The evaluation of performance across all species showed that Novoalign/GATK had the highest median F-score (0.994), lowest sum of ranks (10), the lowest number of errors per million sequenced bases (0.944), and the largest absolute number of TP calls (15,777) (Table 1). However, in this initial simulation, as the reads are error-free and the reference genome is the same as the source of the reads, many pipelines avoid FP calls and report a perfect precision of 1.

### Evaluating SNP-calling pipelines when the genome for alignment diverges from the source of the reads

Owing to the high genomic diversity of some bacterial species, the appropriate selection of reference genomes is non-trivial. To assess how pipeline performance is affected by divergence between the source and reference genomes, SNPs were re-called after mapping all reads to a single representative genome for that species (illustrated in Fig. 1). To identify true variants, closed genomes were aligned against the representative genome using both nucmer [58] and Parsnp [59], with consensus calls identified within 1-to-1 alignment blocks (see Methods). Estimates of the distance between each genome and the representative genome are given in Supplementary Table 2, with the genomic diversity of each species summarized in Supplementary Table 5. We quantified genomic distances using the Mash distance, which reflects the proportion of *k*-mers shared between a pair of genomes as a proxy for average nucleotide divergence [60]. The performance statistics for each pipeline are shown in Supplementary Table 6, with an associated ranked summary in Supplementary Table 7.

In general, aligning reads from 1 strain to a divergent reference leads to a decrease in median F-score and increase in interquartile range of the F-score distribution, with pipeline performance more negatively affected by choice of aligner than caller (Fig. 2B).

**Figure 3:**
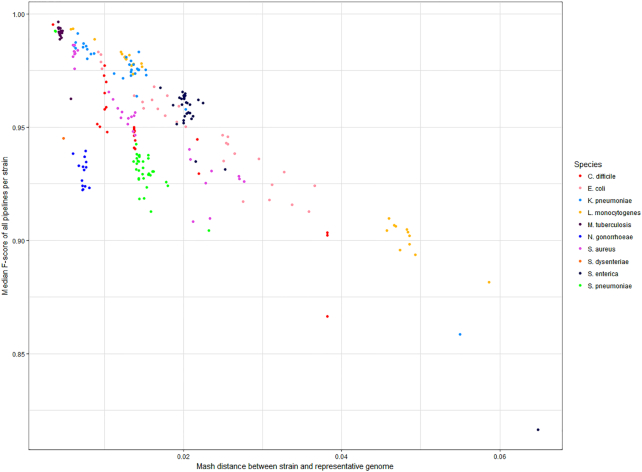
Reduced performance of SNP-calling pipelines with increasing genetic distance between the reads and the reference genome. The median F-score across the complete set of 41 pipelines, per strain, decreases as the distance between the strain and the reference genome increases (assayed as the Mash distance, which is based on the proportion of *k*-mers shared between genomes). Each point indicates the median F-score, across all pipelines, for the genome of 1 strain per species (n = 254 strains). Points are coloured by the species of each strain (n = 10 species). Summary statistics for each pipeline are shown in Supplementary Table 6, performance ranks in Supplementary Table 7, and the genetic distance between strains in Supplementary Table 2. Quantitatively similar results are seen if assaying distance as the total number of SNPs between the strain and representative genome, i.e., the set of strain-specific *in silico* SNPs plus inter-strain SNPs (Supplementary Figure 1).

Although across the full range of genomes, many pipelines show comparable performance (Fig. 2B), there was a strong negative correlation between the Mash distance and F-score (Spearman ρ = −0.72, *P* < 10^−15^; Fig. 3). The negative correlation between F-score and the total number of SNPs between the strain and representative genome, i.e., the set of strain-specific *in silico* SNPs plus inter-strain SNPs, was slightly weaker (ρ = −0.58,*P* < 10^−15^; Supplementary Fig. 1). This overall reduction in performance with increased divergence was more strongly driven by reductions in recall (i.e., by an increased number of FN calls) rather than precision because there was a particularly strong correlation between distance and recall (Spearman ρ = −0.94,*P* < 10^−15^; Supplementary Fig. 2).

Three commonly used pipelines—BWA-mem/Freebayes, BWA-mem/GATK, and Novoalign/GATK—were among the highest performers when the reference genome is also the source of the reads (Table 1 and Supplementary Table 4). However, when the reference diverges from the reads, then considering the 2 “overall performance” measures across the set of 10 species, Snippy instead had both the lowest sum of ranks (20) and the highest median F-score (0.982), along with the lowest number of errors per million sequenced bases (2.627) (Table 1).

Performance per species is presented in Table 2, alongside both the overall sum and range of these ranks per pipeline. Pipelines featuring Novoalign were, in general, consistently high-performing across the majority of species (i.e., having a lower sum of ranks), although they were outperformed by Snippy, which had both strong and uniform performance across all species (Table 2). By contrast, pipelines with a larger range of ranks had more inconsistent performance, such as minimap2/SNVer, which for example performed relatively strongly for *N. gonorrhoeae* but poorly for *S. dysenteriae* (Table 2).

**Table 2:** Overall performance of each pipeline per species, calculated as the sum of 7 ranks, when reads are aligned to a divergent genome

Pipeline	*Clostridiodes difficile*	*Escherichia coli*	*Klebsiella pneumoniae*	*Listeria monocytogenes*	*Mycobacterium tuberculosis*	*Neisseria gonorrhoea*	*Salmonella enterica*	*Shigella dysenteriae*	*Staphylococcus aureus*	*Streptococcus pneumoniae*	Sum of ranks	Range of ranks
snippy *	2	1	1	1	5	1	1	2	1	1	16	4
novoalign/lofreq	1	2	3	10	3	4	2	1	3	2	31	9
novoalign/mpileup	3	3	4	9	2	10	5	4	2	3	45	8
novoalign/16GT	5	5	6	8	8	12	3	18	6	6	77	15
novoalign/snver	4	4	5	12	12	14	4	14	4	10	83	10
minimap2/mpileup	10	6	2	20	9	13	9	9	7	15	100	18
novoalign/strelka	6	9	13	7	13	27	8	11	11	4	109	23
bwa-mem/mpileup	12	14	15	2	7	8	19	17	8	9	111	17
minimap2/strelka	8	11	10	21	15	6	11	12	10	7	111	15
bwa-mem/snver	9	10	11	5	21	2	10	21	14	12	115	19
minimap2/lofreq	20	8	7	18	10	17	18	3	9	14	124	17
novoalign/freebayes	7	13	12	14	1	22	6	24	18	17	134	23
bwa-mem/16GT	22	18	20	6	19	15	17	5	13	8	143	17
bwa-mem/strelka	16	25	22	4	16	5	26	7	17	5	143	22
bwa-mem/lofreq	18	16	19	3	11	20	24	19	5	11	146	21
minimap2/freebayes	14	12	9	15	4	25	7	23	19	18	146	21
minimap2/16GT	21	15	14	16	18	18	16	6	12	13	149	15
minimap2/snver	11	7	8	25	22	3	12	26	15	22	151	23
bwa-mem/freebayes *	15	17	16	13	6	19	13	16	21	16	152	15
novoalign/varscan	13	19	17	17	20	16	15	13	16	21	167	8
bwa-mem/varscan	17	24	21	11	30	9	23	29	23	23	210	21
bwa-mem/platypus	31	23	25	19	36	7	22	10	24	20	217	29
stampy/strelka	24	27	27	22	25	11	32	15	20	19	222	21
minimap2/varscan	19	21	18	29	32	26	21	31	22	25	244	14
novoalign/platypus	29	20	23	23	28	32	14	25	30	27	251	18
minimap2/platypus	23	22	24	34	34	21	20	22	25	29	254	14
stampy/freebayes	26	26	26	24	33	30	29	30	26	24	274	9
bwa-mem/gatk	27	28	32	26	26	31	28	28	27	26	279	6
stampy/mpileup	36	32	29	28	14	23	35	27	31	30	285	22
novoalign/gatk	28	29	31	27	23	34	25	34	28	31	290	11
stampy/lofreq	37	33	30	30	17	29	37	20	32	32	297	20
minimap2/gatk	25	31	33	33	24	35	27	35	34	28	305	11
stampy/gatk	34	34	35	31	27	37	30	32	33	34	327	10
stampy/platypus	38	35	39	35	37	24	33	8	41	39	329	33
novoalign/snvsniffer	33	30	28	32	38	33	31	38	36	33	332	10
stampy/snver	30	39	34	41	29	28	40	37	38	35	351	13
bwa-mem/snvsniffer	32	36	36	38	39	39	34	39	29	38	360	10
stampy/16GT	40	38	37	37	35	36	39	33	39	36	370	7
stampy/varscan	41	40	38	39	31	38	41	36	40	37	381	10
minimap2/snvsniffer	35	37	40	40	40	40	36	40	35	40	383	5
stampy/snvsniffer	39	41	41	36	41	41	38	41	37	41	396	5

* Snippy is based upon a BWA-mem/freebayes pipeline but under default parameters, shows improved performance.

**Figure 4:**
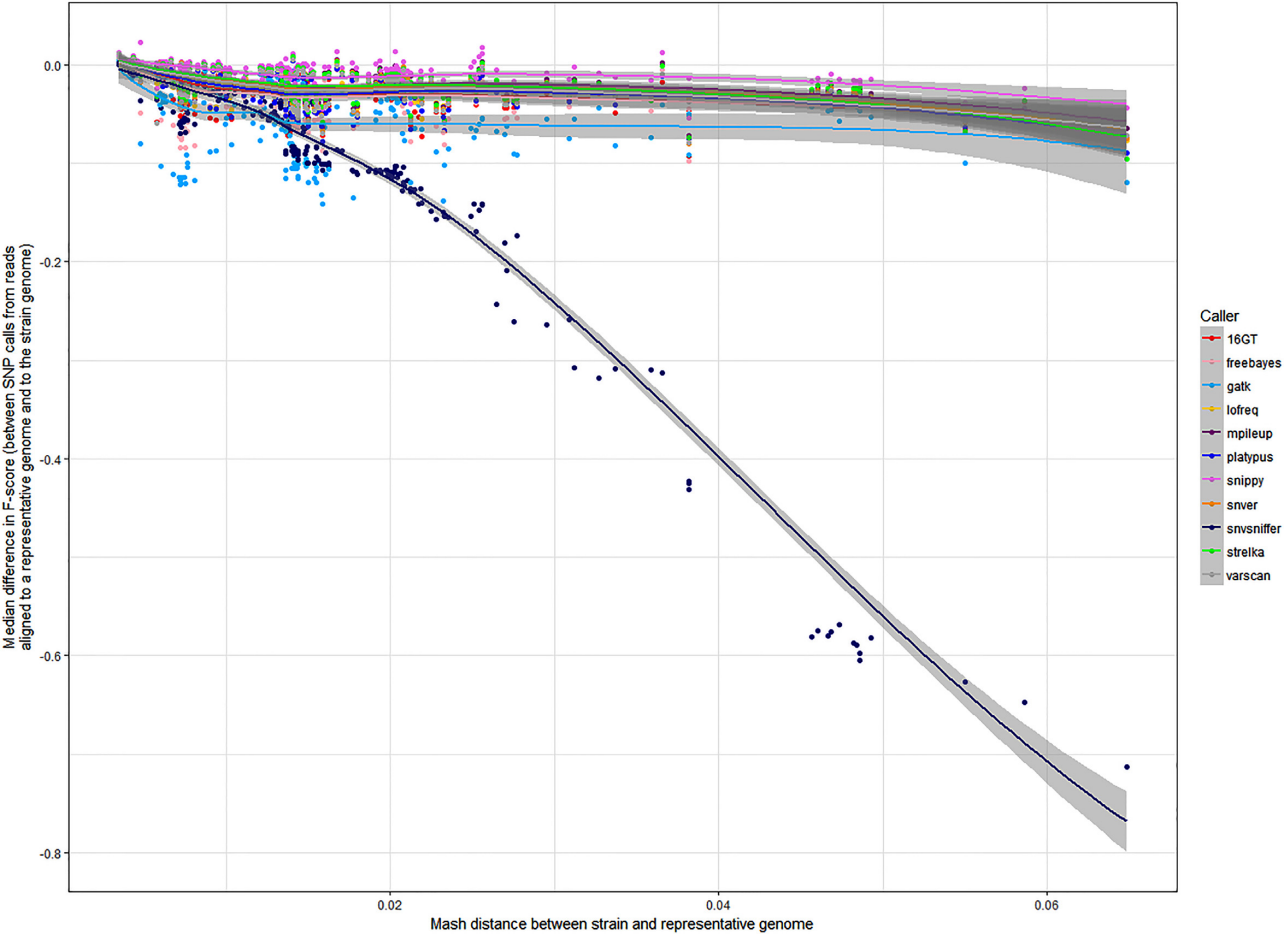
Stability of pipeline performance, in terms of F-score, with increasing genetic distance between the reads and the reference genome. The performance of an SNP-calling pipeline decreases with increasing distance between the genome from which reads are sequenced and the reference genome to which they are aligned. Each point shows the median difference in F-score for a pipeline that calls SNPs when the reference genome is the same as the source of the reads, and when it is instead a representative genome for that species. Points are coloured according to the variant caller in each pipeline, with those towards the top of the figure less affected by distance. Lines fitted using LOESS smoothing, with the grey band representing the 0.95 confidence interval.

Although, in general, the accuracy of SNP calling declined with increasing genetic distances, some pipelines were more stable than others. If considering the median difference in F-score between SNP calls made using the same versus a representative genome, Snippy had smaller differences as the distance between genomes increased (Fig. 4).

**Figure 5:**
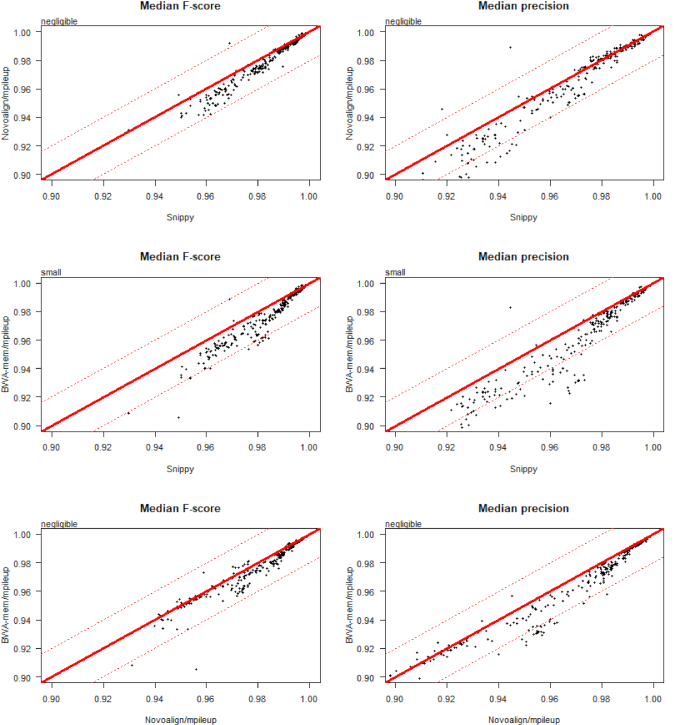
Head-to-head performance comparison of 3 pipelines using simulated data, on the basis of precision, recall, and F-score. This figure directly compares the performance of 3 pipelines using simulated data: Snippy, Novoalign/mpileup, and BWA/mpileup. Each point indicates the median F-score, precision, or recall, for the genome of 1 strain per species (n = 254 strains). Raw data for this figure are given in Supplementary Table 6. Text in the top left of each figure is an interpretation of the difference between each pair of distributions, obtained using the R package “effsize,” which applies the non-parametric effect size estimator Cliff delta to the results of a Mann-Whitney *U* test. The line y = x is shown in solid red. The lines y+0.02 = x and y-0.02 = x are shown in dotted red. An expanded version of this figure, comparing 40 pipelines relative to Snippy, is given as Supplementary Figure 4.

The highest-ranked pipelines in Table 2 had small, but practically unimportant, differences in median F-score and so are arguably equivalently strong candidates for a “general purpose” SNP-calling solution. For instance, on the basis of F-score alone the performance of Novoalign/mpileup was negligibly different from that of BWA-mem/mpileup (Fig. 5). However, when directly comparing pipelines, similarity of F-score distributions (see Fig. 2B) can conceal larger differences in either precision or recall, categorized using the effect size estimator Cliff delta [61, 62]. Thus, certain pipelines may be preferred if the aim is to minimize FP (e.g., for transmission analysis) or maximize TP (e.g., to identify antimicrobial resistance loci) calls. For instance, although Snippy (the top-ranked pipeline in Table 2) is negligibly different from Novoalign/mpileup (the third-ranked pipeline) in terms of F-score and precision, the former is more sensitive (Fig. 5).

### Comparable accuracy of SNP-calling pipelines using real rather than simulated sequencing data

We used real sequencing data from a previous study comprising 16 environmentally sourced gram-negative isolates (all *Enterobacteriaceae*), derived from livestock farms, sewage, and rivers, and cultures of 2 reference strains (*K. pneumoniae* subsp. *pneumoniae* MGH 78,578 and *E. coli* CFT073), for which closed hybrid *de novo* assemblies were generated using both Illumina paired-end short reads and Nanopore long reads [63]. Source locations for each sample, species predictions, and NCBI accession numbers are detailed in Supplementary Table 8. The performance statistics for each pipeline are provided in Supplementary Table 9, with an associated ranked summary in Supplementary Table 10.

**Figure 6:**
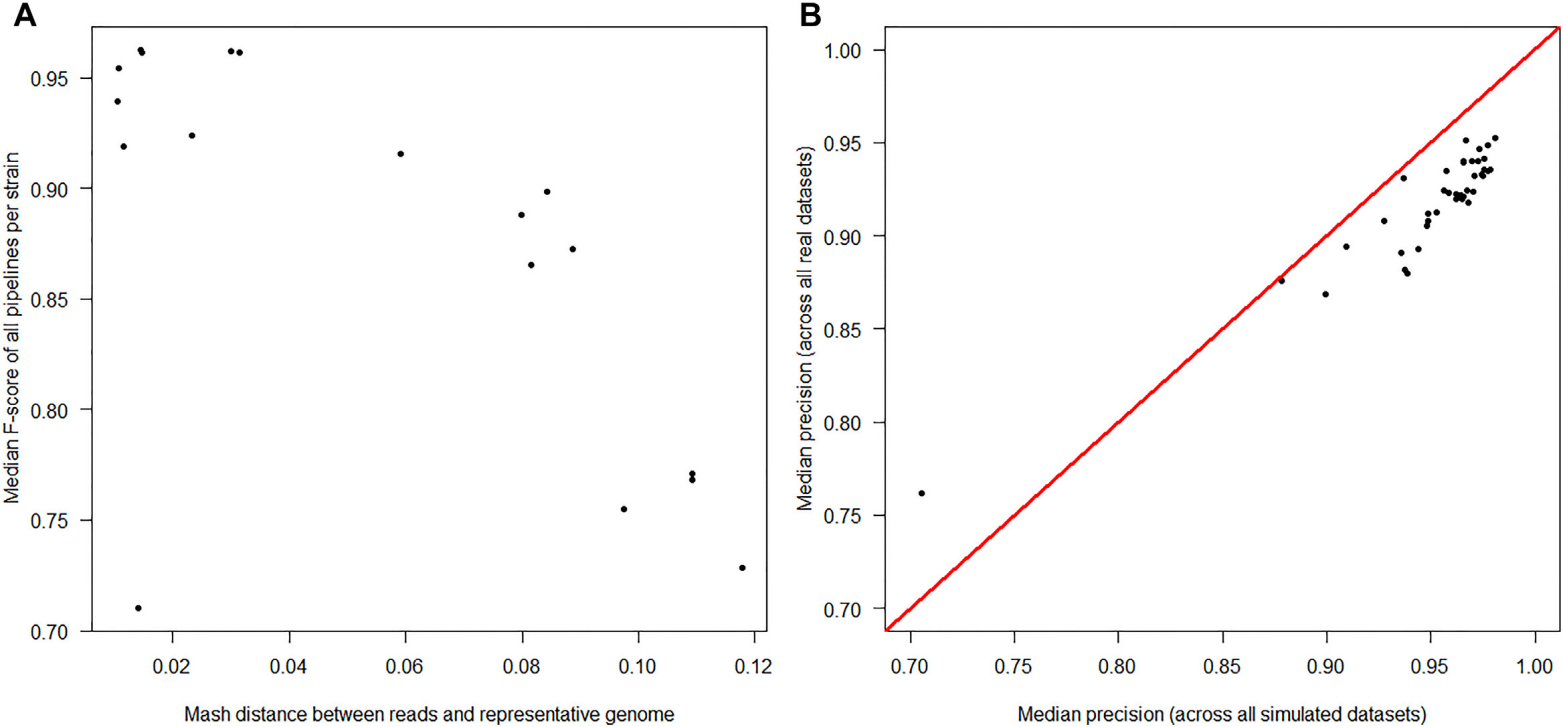
Similarity of performance for pipelines evaluated using both simulated and real sequencing data. Panel A shows that pipelines evaluated using real sequencing data show reduced performance with increasing Mash distances between the reads and the reference genome, similar to that observed with simulated data (see Figure 3A). Each point indicates the median F-score, across all pipelines, for the genome of an environmentally sourced/reference isolate (detailed in Supplementary Table 8). Panel B shows that pipelines evaluated using real and simulated sequencing data have comparable accuracy. Each point shows the median precision of each of 41 pipelines, calculated across both a divergent set of 254 simulated genomes (2–36 strains from 10 clinically common species) and 18 real genomes (isolates of *Citrobacter, Enterobacter*, *Escherichia*, and *Klebsiella*). The outlier pipeline, with lowest precision on both real and simulated data, is Stampy/Freebayes. Raw data for this figure are available in Supplementary Tables 6 (simulated genomes) and 9 (real genomes).

Lower performance was anticipated for all pipelines, particularly for *Citrobacter* and *Enterobacter* isolates, which had comparatively high Mash distances (>0.08) between the reads and the representative genome (Supplementary Table 8), far greater than those in the simulations (241 of the 254 simulated genomes had a Mash distance to the representative genome of <0.04; Supplementary Table 2). Consistent with the simulations (Fig. 3A), there was a strong negative correlation between Mash distance and the median F-score across all pipelines (Spearman ρ = −0.83, *P* = 3.36 × 10^−5^; Fig. 6A), after excluding 1 prominent outlier (*E. coli* isolate RHB11-C04; see Supplementary Table 8).

Notably, the median precision of each pipeline, if calculated across the divergent set of simulated genomes, strongly correlated with the median precision calculated across the set of real genomes (Spearman ρ = 0.83, *P* = 2.81 × 10^−11^; Fig. 6B). While a weaker correlation was seen between simulated and real datasets on the basis of recall (Spearman ρ = .41, *P* = 0.007), this is consistent with the high diversity of *Enterobacteriaceae*, and the accordingly greater number of FN calls with increased divergence (Supplementary Fig. 2).

Overall, this suggests that the accuracy of a given pipeline on simulated data is a reasonable proxy for its performance on real data. While the pipelines that performed more poorly on simulated data similarly performed more poorly on real data, the top-ranked pipelines differed, predominantly featuring BWA-mem, rather than Novoalign, as an aligner (Supplementary Table 10). In both cases, however, among the consistently highest-performing pipelines was Snippy.

**Figure 7:**
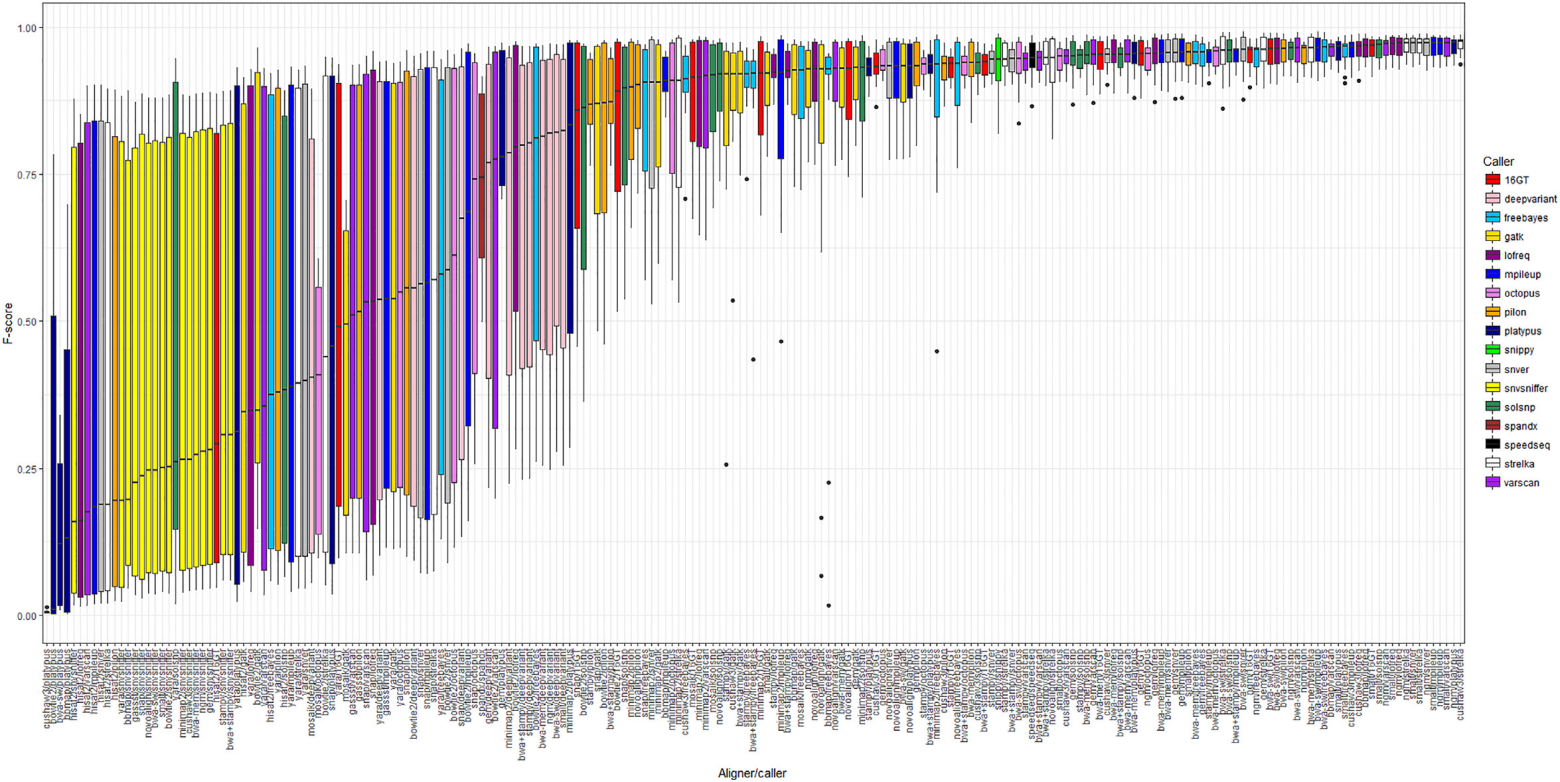
Median F-score per pipeline using real sequencing data, and when the reference genome for alignment can diverge considerably from the source of the reads. This figure shows the F-score distribution of 209 pipelines evaluated using real sequencing data sourced from the REHAB project and detailed in [63]. This dataset comprises 16 environmentally sourced gram-negative isolates (all *Enterobacteriaceae*), and cultures of 2 reference strains (*K. pneumoniae* subsp. *pneumoniae* MGH 78,578 and *E. coli* CFT073). For this figure, data from 1 outlier, *E. coli* isolate RHB11-C04, were excluded. Raw data for this figure are available as Supplementary Table 9, with summary statistics for each pipeline detailed in Supplementary Table 10. Genomes are detailed in Supplementary Table 8. Boxes represent the interquartile range of F-score, with midlines representing the median. Upper and lower whiskers extend, respectively, to the largest and smallest values no further than 1.5x the interquartile range. Data beyond the ends of each whisker are outliers and plotted individually.

Quantitatively similar results were found when quintupling the scope of this evaluation to include 209 pipelines (Fig. 7). With this gram-negative dataset, the most consistently highly-performing pipelines had little variation in F-score, irrespective of the 10-fold difference in Mash distances between reads and representative genome (Supplementary Table 8). Particularly highly-performing pipelines in the expanded dataset used the aligners NextGenMap or SMALT, and/or the variant callers LoFreq, mpileup, or Strelka (Fig. 7).

## Discussion

### Reference genome selection strongly affects SNP-calling performance

Here we initially evaluated 41 SNP-calling pipelines, the combination of 4 aligners with 10 callers, plus 1 “all-in-one” tool, Snippy, using reads simulated from 10 clinically relevant species. These reads were first aligned back to their source genome and SNPs called. As expected under these conditions, the majority of SNP-calling pipelines showed high precision and sensitivity, although between-species variation was prominent.

We next expanded the scope of the evaluation to 209 pipelines (representing the addition of 12 aligners, 4 callers, and 2 “all-in-one” pipelines, SpeedSeq and SPANDx) and introduced a degree of divergence between the reference genome and the reads, analogous to having an accurate species-level classification of the reads but no specific knowledge of the strain. For the purposes of this study, we assumed that reference genome selection was essentially arbitrary, equivalent to a community standard representative genome. Such a genome can differ significantly from the sequenced strain, which complicates SNP calling by introducing inter-specific variation between the sequenced reads and the reference. Importantly, all pipelines in this study are expected to perform well if evaluated with human data, i.e., when there is a negligible Mash distance between the reads and the reference. For example, the mean Mash distance between human assembly GRCh38.p12 and the 3 Ashkenazi assemblies of the Genome In A Bottle dataset (deep sequencing of a mother, father, and son trio [64–66], available under European Nucleotide Archive study accession PRJNA200694 and GenBank assembly accessions GCA_001549595.1, GCA_001549605.1, and GCA_001542345.1, respectively) is 0.001 (i.e., consistent with previous findings that the majority of the human genome has ∼0.1% sequence divergence [67]). Notably, the highest-performing pipeline when reads were aligned to the same genome from which they were simulated, Novoalign/GATK, was also that used by the Genome In A Bottle consortium to align human reads to the reference [64].

While tools initially benchmarked on human data, such as SNVSniffer [49], can in principle also be used on bacterial data, this study shows that in practice many perform poorly. For example, the representative *C. difficile* strain, 630, has a mosaic genome, ∼11% of which comprises mobile genetic elements [56]. With the exception of reads simulated from *C. difficile* genomes that are erythromycin-sensitive derivatives of 630 (strains 630Derm and 630deltaerm; see [68]), aligning reads to 630 compromises accurate SNP calling, resulting in a lower median F-score across all pipelines (Fig. 3). We also observed similar decreases in F-score for more recombinogenic species such as *N. gonorrhoeae*, which has a phase-variable gene repertoire [69] and has been used to illustrate the “fuzzy species” concept, that recombinogenic bacteria do not form clear and distinct isolate clusters as assayed by phylogenies of common housekeeping loci [70, 71]. By contrast, for clonal species, such as those within the *M. tuberculosis* complex [72], the choice of reference genome has negligible influence on the phylogenetic relationships inferred from SNP calls [73] and, indeed, minimal effect on F-score.

In general, more diverse species have a broader range of Mash distances on Fig. 2A (particularly notable for *E. coli*), as do those forming distinct phylogroups, such as the 2 clusters of *L. monocytogenes*, consistent with the division of this species into multiple primary genetic lineages [74–76].

Therefore, 1 major finding of this study is that, irrespective of the core components within an SNP-calling pipeline, the selection of reference genome has a critical effect on output, particularly for more recombinogenic species. This can to some extent be mitigated by using variant callers that are more robust to increased distances between the reads and the reference, such as Freebayes (used by Snippy and SpeedSeq).

A suboptimal choice of reference genome has previously been shown to result in mapping errors, leading to biases in allelic proportions [77]. Heterologous reference genomes are in general suboptimal for read mapping, even when there is strict correspondence between orthologous regions, with short reads particularly vulnerable to FP alignments [78]. There is also an inverse relationship between TP SNP calls and genetic distance, with a greater number of FP calls when the reads diverge from the reference genome [21].

### Study limitations

The experimental design made several simplifying assumptions regarding pipeline usage. Most notably, when evaluating SNP calling when the reference genome diverges from the source of the reads, we needed to convert the coordinates of one genome to those of another, doing so by whole-genome alignment. We took a similar approach to that used to evaluate Pilon, an all-in-one tool for correcting draft assemblies and variant calling [45], which made whole-genome alignments of the *M. tuberculosis* F11 and H37Rv genomes and used the resulting set of inter-strain variants as a truth set for benchmarking (a method we also used when evaluating each pipeline on real data). While this approach assumes a high degree of contiguity for the whole-genome alignment, there are nevertheless significant breaks in synteny between F11 and H37Rv, with 2 regions deemed particularly hypervariable, in which no variant could be confidently called [45]. For the strain–to–representative genome alignments in this study, we considered SNP calls only within 1-to-1 alignment blocks and cannot exclude the possibility that repetitive or highly mutable regions within these blocks have been misaligned. However, we did not seek to identify and exclude SNPs from these regions because, even if they were present, this would have a systematic negative effect on the performance of each pipeline. To demonstrate this, we recalculated each performance metric for the 209 pipelines evaluated using real sequencing data after identifying, and masking, repetitive regions of the reference genome with self-self BLASTn (as in [79]). As we already required reference bases within each 1-to-1 alignment block to be supported by both nucmer and Parsnp calls (i.e., implicitly masking ambiguous bases), we found that repeat-masking the reference genome had negligible effect on overall F-score although marginally improved precision (see Supplementary Text 1). However, it is important to note that the parameters used for repeat-masking will determine which paralogues will be successfully masked. For the purpose of this study, we used reasonably conservative parameters (detailed in Supplementary Text 1) and so expect to have primarily masked more similar paralogues. The likelihood of mis-mapping (and thereby FP SNP calling) would increase among more divergent paralogues, although optimizing parameters to detect these is non-trivial. More lenient repeat-masking parameters, in masking more divergent positions, would also reduce the number of true SNPs it is possible to call.

Furthermore, when aligning reads from 1 genome to a different genome, it is not possible to recover all possible SNPs introduced with respect to the former because some will be found only within genes unique to the original genome (of which there can be many because bacterial species have considerable genomic diversity; see Supplementary Table 5). Nevertheless, there is a strong relationship between the total number of SNPs introduced *in silico* into 1 genome and the maximum number of SNPs it is possible to call should reads instead be aligned to a divergent genome (Supplementary Fig. 3). In any case, this does not affect the evaluation metrics used for pipeline evaluation, such as F-score, because these are based on proportional relationships of TP, FP, and FN calls at variant sites. However, we did not count true-negative calls (and thereby assess pipeline specificity) because these can only be made at reference sites, a far greater number of which do not exist when aligning between divergent genomes.

While the programs chosen for this study are in common use and the findings generalizable, it is also important to note that they are a subset of the tools available (see Supplementary Text 1). It is also increasingly common to construct more complex pipelines that call SNPs with 1 tool and structural variants with another (e.g., in [80]). Here, our evaluation concerned only accurate SNP calling, irrespective of the presence of structural variants introduced by suboptimal reference genome selection (i.e., by aligning the reads to a divergent genome) and so does not test dedicated indel-calling algorithms. Previous indel-specific variant-calling evaluations, using human data, have recommended Platypus [8] or, for calling large indels at low read depths, Pindel [81].

Many of the findings in this evaluation are also based on simulated error-free data for which there was no clear need for pre-processing quality control. While adapter removal and quality-trimming reads are recommended precautionary steps prior to analysing non-simulated data, previous studies differ as to whether pre-processing increases the accuracy of SNP calls [82], has minimal effect upon them [83], or whether benefits instead depend upon the aligner and reference genome used [21]. While more realistic datasets would be subject to sequencing error, we also expect this to be minimal: Illumina platforms have a per-base error rate <0.01% [84]. Accordingly, when comparing pipelines taking either error-free or error-containing reads as input, sequencing error had negligible effect on performance (see Supplementary Text 1).

We have also assumed that given the small genome sizes of bacteria, a consistently high depth of coverage is expected in non-simulated datasets, and so have not evaluated pipeline performance on this basis (discussed further in Supplementary Text 1). In any case, a previous study found that with simulated NextSeq reads, variant-calling sensitivity was largely unaffected by increases in coverage [11]. It has also been reported that random polymerase errors have minimal effect on variant calls for sequencing depths >20-fold and that these are primarily of concern only when calling minor variants [77].

Finally, so as to approximate “out of the box” use conditions, we made a minimal-effort application of each program with no attempt at species-specific optimization. Had we optimized the individual components of an analytic pipeline (which, although often structured around, are not limited to 1 aligner and 1 caller), we could conceivably reduce the high variance in F-score when SNP calling from real data which, in this study, was notably divergent (see Fig. 7). For instance, DeepVariant [41], a TensorFlow machine-learning–based variant caller, had highly variable performance on real data but required as input a training model made using a deep neural network. At the time of use, there was currently no production-grade DeepVariant training pipeline (the default training model supplied with DeepVariant, and used in this study, was based on human data), nor were there a large enough number of non-simulated, bacterial truth sets on which to train it. As such, we expect the performance of DeepVariant to have been under-estimated in this evaluation. Most notably, NextGenMap/DeepVariant was the most precise of the 209 pipelines evaluated on (divergent) real data (mean precision = 0.9715), although this pipeline had comparatively low recall and an accordingly poor F-score (Supplementary Table 10).

In this study we sought to use all aligners and callers uniformly, with equivalent quality control steps applied to all reads. To that end, while direct comparisons of any aligner/caller pipeline with “all-in-one” tools (such as Snippy, SPANDx, and SpeedSeq) are possible, the results should be interpreted with caution. This is because it is in principle possible to improve the performance of the former through additional quality control steps—i.e., compared to an “all-in-one” tool, it is not necessarily the aligner or caller alone to which any difference in performance may be attributed. For instance, although Snippy and SpeedSeq use BWA-mem and Freebayes, both tools are distinct from the BWA-mem/Freebayes pipeline used in this study (Fig. 7 and Supplementary Table 10). This is because they implement additional steps between the BWA and Freebayes components, as well as altering the default parameters relative to standalone use. Snippy, for example, uses samclip [85] to post-process the BAM file produced by BWA-mem, removing clipped alignments in order to reduce FP SNPs near structural variants.

### Recommendations for bacterial SNP calling

Our results emphasize that one of the principal difficulties of alignment-based bacterial SNP calling is not pipeline selection per se but optimal reference genome selection (or, alternatively, its *de novo* creation, not discussed further). If assuming all input reads are from a single, unknown, origin, then in principle a reference genome could be predicted using a metagenomic classifier such as Centrifuge [86], CLARK [87], Kaiju [88], or Kraken [89]. However, correctly identifying the source genome from even a set of single-origin reads is not necessarily simple, with the performance of read classifiers depending in large part on the sequence database they query (such as, e.g., EMBL proGenomes [90] or NCBI RefSeq [91]), which can vary widely in scope, redundancy, and degree of curation (see performance evaluations [92, 93]). This is particularly evident among the *Citrobacter* samples in the real dataset, with 3 methods each making different predictions (Supplementary Table 8). Specialist classification tools such as Mykrobe [94] use customized, tightly curated allele databases and perform highly for certain species (in this case, *M. tuberculosis* and *S. aureus*) although by definition do not have wider utility. An additional complication would also arise from taxonomic disputes such as, for example, *Shigella* spp. being essentially indistinct from *E. coli* [95].

One recommendation, which is quick and simple to apply, would be to test which of a set of candidate reference genomes is most suitable by estimating the distance between each genome and the reads. This can be accomplished using Mash [60], which creates “sketches” of sequence sets (compressed representations of their *k*-mer distributions) and then estimates the Jaccard index (that is, the fraction of shared *k*-mers) between each pair of sequences. Mash distances are a proxy both for average nucleotide identity [60] and measures of genetic distance derived from the whole-genome alignment of genome pairs (Supplementary Table 2), correlating strongly with the total number of SNPs between the strain genome and the representative genome (Spearman ρ = 0.97, *P* < 10^−15^), and to a reasonable degree with the proportion of bases unique to the strain genome (Spearman ρ = = 0.48, *P* < 10^−15^). More closely related genomes would have lower Mash distances and so be more suitable as reference genomes for SNP calling. This would be particularly appropriate if, for example, studying transmission events as a closely related reference would increase specificity, irrespective of the aligner or caller used. For larger studies that require multiple samples to be processed using a common reference, the choice of reference genome could be one that “triangulates” between the set of samples—i.e., has on average a similar distance to each sample, rather than being closer to some and more distant from others.

Using a highly divergent genome (such as the representative *Enterobacter* genomes in the real dataset, each of which differs from the reads by a Mash distance >0.1; Supplementary Table 8) is analogous to variant calling in a highly polymorphic region, such as the human leukocyte antigen, which shows >10% sequence divergence between haplotypes [67] (i.e., even for pipelines optimized for human data—the majority in this study—this would represent an anomalous use case).

Prior to using Mash (or other sketch-based distance estimators, such as Dashing [96] or FastANI [97]), broad-spectrum classification tools such as Kraken could be used to narrow down the scope of the search space to a set of fully sequenced candidate genomes, i.e., those genomes of the taxonomic rank to which the highest proportion of reads could be assigned with confidence. This approach is similar to that implemented by the Python package PlentyOfBugs [98], which, assuming the species or genus is already known, automates the process of downloading and sketching candidate genomes to create a database for querying with Mash.

In the future, reads from long-read sequencing platforms, such as Oxford Nanopore and Pacific Biosciences, are less likely to be ambiguously mapped within a genomic database and so in principle are simpler to classify (sequencing error rate notwithstanding), making it easier to select a suitable reference genome. However, long-read platforms can also, in principle if not yet routinely, generate complete *de novo* bacterial genomes [99] for downstream SNP calling, possibly removing the need to choose a reference entirely. Similarly, using a reference pan-genome instead of a singular representative genome could also maximize the number of SNP calls by reducing the number of genes not present in the reference [100]. A popular means of representing the pan-genome, as used by tools such as Roary [101], is as a collection of individual consensus sequences, ostensibly genes but more specifically open reading frames with protein-coding potential. This use of consensus sequences could also reduce the number of nucleotide differences between a set of sequenced reads (which may be from a highly divergent strain) and the (consensus) reference.

An alternative approach to reducing errors introduced when using a single reference genome could be to merge results from multiple reference genomes (the approach taken by REALPHY to reconstruct phylogenies from bacterial SNPs [102]) or from multiple aligners and/or callers, obtaining consensus calls across a set of methods. This is the approach taken by the NASP pipeline [103], which can integrate data from any combination of the aligners Bowtie2, BWA-mem, Novoalign, and SNAP, and the callers GATK, mpileup, SolSNP, and VarScan (ensemble approaches have similarly been used for somatic variant calling, e.g., by SomaticSeq [104]).

If considering the overall performance of a pipeline as the sum of the 7 different ranks for the different metrics considered, then averaged across the full set of species’ genomes, the highest-performing pipelines are, with simulated data, Snippy and those using Novoalign in conjunction with LoFreq or mpileup (Table 2), and with real (more divergent) data, those using NextGenMap or SMALT in conjunction with LoFreq, mpileup, or Strelka (Supplementary Table 10).

Some of the higher-performing tools apply error correction models that also appear suited to bacterial datasets with high SNP density, despite their original primary use case being in different circumstances. For instance, SNVer (which, in conjunction with BWA-mem, ranks second to Snippy for *N. gonorrhoeae*; see Table 2) implements a statistical model for calling SNPs from pooled DNA samples, where variant allele frequencies are not expected to be either 0, 0.5, or 1 [48]. SNP calling from heterogeneous bacterial populations with high mutation rates, in which only a proportion of cells may contain a given mutation, is also conceptually similar to somatic variant calling in human tumours, where considerable noise is expected [77]. This is a recommended use case for Strelka, which performed highly on real (and particularly divergent) data, being among the top-performing pipelines when paired with many aligners (Fig. 7).

Irrespective of pipeline used, increasing Mash distances between the reads and the reference increases the number of FN calls (Supplementary Fig. 2). Nevertheless, Snippy, which uses Freebayes, is particularly robust to this, being among the most sensitive pipelines when evaluated using simulated data (Fig. 5 and Supplementary Fig. 4). Notably, Freebayes is haplotype-based, calling variants based on the literal sequence of reads aligned to a particular location, so avoiding the problem of 1 read having multiple possible alignments (increasingly likely with increasing genomic diversity) but only being assigned to 1 of them. However, as distance increases further, it is likely that reads will cease being misaligned (which would otherwise increase the number of FP calls), but rather they will not be aligned at all, being too dissimilar to the reference genome.

With an appropriate selection of reference genome, many of these higher-performing pipelines could be optimized to converge on similar results by tuning parameters and post-processing VCFs with specific filtering criteria, another routine task for which there are many different choices of application [105–108]. In this respect, the results of this study should be interpreted as a range-finding exercise, drawing attention to those SNP-calling pipelines that, under default conditions, are generally higher-performing and that may be most straightforwardly optimized to meet user requirements.

## Conclusions

We have performed a comparison of SNP-calling pipelines across both simulated and real data in multiple bacterial species, allowing us to benchmark their performance for this specific use. We find that all pipelines show extensive species-specific variation in performance, which has not been apparent from the majority of existing, human-centred, benchmarking studies. While aligning to a single representative genome is common practice in eukaryotic SNP calling, in bacteria the sequence of this genome may diverge considerably from the sequence of the reads. A critical factor affecting the accuracy of SNP calling is thus the selection of a reference genome for alignment. This is complicated by ambiguity as to the strain of origin for a given set of reads, which is perhaps inevitable for many recombinogenic species, a consequence of the absence (or impossibility) of a universal species concept for bacteria (but see [109]). For many clinically common species, excepting *M. tuberculosis*, the use of standard “representative” reference genomes can compromise accurate SNP calling by disregarding genomic diversity. By first considering the Mash distance between the reads and a candidate set of reference genomes, a genome with minimal distance may be chosen that, in conjunction with one of the higher-performing pipelines, can maximize the number of true variants called.

## Materials and Methods

### Simulating truth sets of SNPs for pipeline evaluation

A total of 264 genomes, representing a range of strains from 10 bacterial species, and their associated annotations, were obtained from the NCBI Genome database [110] ([111], accessed 16 August 2018), as detailed in Supplementary Table 2. One genome per species is considered to be a representative genome (criteria detailed at [112], accessed 16 August 2018), indicated in Supplementary Table 2. Strains with incomplete genomes (i.e., assembled only to the contig or scaffold level) or incomplete annotations (i.e., with no associated GFF, necessary to obtain gene coordinates) were excluded, as were those with multiple available genomes (i.e., the strain name was not unique). After these filters were applied, all species were represented by ∼30 complete genomes (28 *C. difficile*, 29 *M. tuberculosis*, and 36 *S. pneumoniae*), with the exceptions of *N. gonorrhoeae* (n = 15) and *S. dysenteriae* (n = 2). For the 5 remaining species (*E. coli*, *K. pneumoniae*, *L. monocytogenes*, *S. aureus*, and *S. enterica*), there are >100 usable genomes each. Because it was not computationally tractable to test every genome, we chose a subset of isolates based on stratified selection by population structure. We created all-against-all distance matrices using the “triangle” component of Mash v2.1 [60], then constructed dendrograms (Supplementary Figs 5–9) from each matrix using the neighbour-joining method, as implemented in MEGA v7.0.14 (MEGA Software, RRID:SCR_000667) [113]. By manually reviewing the topology, 30 isolates were chosen per species to create a representative sample of its diversity.

For each genome used in this study, we excluded, if present, any non-chromosomal (i.e., circular plasmid) sequence. A simulated version of each core genome, with exactly 5 randomly generated SNPs per genic region, was created using Simulome v1.2 [114] with parameters –whole_genome = TRUE –snp = TRUE –num_snp = 5. Because the coordinates of some genes overlap, not all genes will contain simulated SNPs. The number of SNPs introduced into each genome (from ∼8000 to 25,000) and the median distance between SNPs (from ∼60 to 120 bases) is detailed in Supplementary Table 2.

The coordinates of each SNP inserted into a given genome are, by definition, genome- (that is, strain-) specific. As such, it is straightforward to evaluate pipeline performance when reads from 1 genome are aligned to the same reference. However, to evaluate pipeline performance when reads from 1 genome are aligned to the genome of a divergent strain (i.e., the representative genome of that species), the coordinates of each strain's genome need to be converted to representative genome coordinates. To do so, we made whole-genome (core) alignments of the representative genome to both versions of the strain genome (1 with and 1 without SNPs introduced *in silico*) using nucmer and dnadiff, components of MUMmer v4.0.0beta2 [58], with default parameters (illustrated in Fig. 1). For 1-to-1 alignment blocks, differences between each pair of genomes were identified using MUMmer show-snps with parameters -Clr -x 1, with the tabular output of this program converted to VCF by the script MUMmerSNPs2VCF.py [115]. The 2 resulting VCFs contain the location of all SNPs relative to the representative genome (i.e., inclusive of those introduced *in silico*), and all inter-strain variants, respectively. We excluded from further analysis 2 strains with poor-quality strain–to–representative whole-genome alignments, both calling <10% of the strain-specific *in silico* SNPs (Supplementary Table 11). The proportion of *in silico* SNPs recovered by whole-genome alignment is detailed in Supplementary Table 11 and is, in general, high: of the 254 whole-genome alignments of non-representative to representative strains across the 10 species, 222 detect >80% of the *in silico* SNPs and 83 detect >90%. For the purposes of evaluating SNP-calling pipelines when the reference genome differs from the reads, we are concerned only with calling the truth set of *in silico* SNPs and so discard inter-strain variants (see below). More formally, when using each pipeline to align reads to a divergent genome, we are assessing the concordance of its set of SNP calls with the set of nucmer calls. However, it is possible that for a given call, 1 or more of the pipelines are correct and nucmer is incorrect. To reduce this possibility, a parallel set of whole-genome alignments were made using Parsnp v1.2 with default parameters [59], with the exported SNPs contrasted with the nucmer VCF.

Thus, when aligning to a divergent genome, the truth set of *in silico* SNPs (for which each pipeline is scored for TP calls) are those calls independently identified by both nucmer and Parsnp. Similarly, the set of inter-strain positions are those calls made by 1 or both of nucmer and Parsnp. Because we are not concerned with the correctness of these calls, the lack of agreement between the 2 tools is not considered further; rather, this establishes a set of ambiguous positions, which are discarded when VCFs are parsed.

Simulated SNP-containing genomes, sets of strain–to–representative genome SNP calls (made by both nucmer and Parsnp), and the final truth sets of SNPs are available in Supplementary Dataset 1 (hosted online via the Oxford Research Archive [116]).

### Evaluating SNP-calling pipelines using simulated data

From each of 254 SNP-containing genomes, 3 sets of 150-bp and 3 sets of 300-bp paired-end data were simulated using wgsim, a component of SAMtools v1.7 (SAMTOOLS, RRID:SCR_002105) [20]. This requires an estimate of average insert size (the length of DNA between the adapter sequences), which in real data is often variable, being sensitive to the concentration of DNA used [117]. For read length *x*, we assumed an insert size of 2.2*x*; i.e., for 300-bp reads, the insert size is 660 bp (Illumina paired-end reads typically have an insert longer than the combined length of both reads [117]). The number of reads simulated from each genome is detailed in Supplementary Table 3 and is equivalent to a mean 50-fold base-level coverage, i.e., (50 × genome length)/read length.

Perfect (error-free) reads were simulated from each SNP-containing genome using wgsim parameters -e 0 -r 0 -R 0 -X 0 -A 0 (respectively, the sequencing error rate, mutation rate, fraction of indels, probability an indel is extended, and the fraction of ambiguous bases allowed).

Each set of reads was then aligned both to the genome of the same strain and to the representative genome of that species (from which the strain will diverge), with SNPs called using 41 different SNP-calling pipelines (10 callers each paired with 4 aligners, plus the self-contained Snippy). The programs used, including version numbers and sources, are detailed in Supplementary Table 1, with associated command lines in Supplementary Text 1. All pipelines were run using a high-performance cluster employing the Open Grid Scheduler batch system on Scientific Linux 7. No formal assessment was made of pipeline run time or memory usage. This was because given the number of simulations it was not tractable to benchmark run time using, for instance, a single core. The majority of programs in this study permit multithreading (all except the callers 16GT, GATK, Platypus, SNVer, and SNVSniffer) and so are in principle capable of running very rapidly. We did not seek to optimize each tool for any given species and so made only a minimum-effort application of each pipeline, using default parameters and minimal VCF filtering (see below). This is so that we obtain the maximum possible number of TP calls from each pipeline under reasonable use conditions.

While each pipeline comprises 1 aligner and 1 caller, there are several ancillary steps common in all cases. After aligning reads to each reference genome, all BAM files were cleaned, sorted, had duplicate reads marked, and were indexed using Picard Tools v2.17.11 (Picard, RRID:SCR_006525), [118] CleanSam, SortSam, MarkDuplicates, and BuildBamIndex, respectively. We did not add a post-processing step of local indel realignment (common in older evaluations, e.g., [12]) because this had a negligible effect on pipeline performance, with many variant callers (including GATK HaplotypeCaller [25] [GATK, RRID:SCR_001876] and Freebayes [FreeBayes, RRID:SCR_010761]) already incorporating a method of haplotype assembly (see Supplementary Text 1).

Each pipeline produces a VCF as its final output. As with a previous evaluation [15], all VCFs were regularized using the vcfallelicprimitives module of vcflib v1.0.0-rc2 [119], so that different representations of the same indel or complex variant were not counted separately (these variants can otherwise be presented correctly in multiple ways). This module splits adjacent SNPs into individual SNPs, left-aligns indels, and regularizes the representation of complex variants. The set of non-regularized VCFs cannot be meaningfully compared (see Supplementary Text 1).

Different variant callers populate their output VCFs with different contextual information. Before evaluating the performance of each pipeline, all regularized VCFs were subject to minimal parsing to retain only high-confidence variants. This is because many tools record variant sites even if they have a low probability of variation, under the reasonable expectation of parsing. Some tools (including Snippy and SNVer) apply their own internal set of VCF filtering criteria, giving the user the option of a “raw” or “filtered” VCF; in such cases, we retain the filtered VCF as the default recommendation. Where possible, (additional) filter criteria were applied as previously used by, and empirically selected for, COMPASS [120], an analytic pipeline employing Stampy and mpileup for base-calling non-repetitive core genome sites (outlined in Supplementary Text 1 with filter criteria described in [121] and broadly similar to those recommended by a previous study for maximizing SNP validation rate [122]). No set of generic VCF hard filters can be uniformly applied because each caller quantifies different metrics (such as the number of forward and reverse reads supporting a given call) and/or reports the outcome of a different set of statistical tests, making filtering suggestions on this basis. For instance, in particular circumstances, GATK suggests filtering on the basis of the fields “FS,” “MQRankSum,” and “ReadPosRankSum,” which are unique to it (detailed at [123]). Where the relevant information was included in the VCF, SNPs were required to have (i) a minimum Phred score of 20, (ii) ≥5 reads mapped at that position, (iii) ≥1 read in each direction in support of the variant, and (iv) ≥75% of reads supporting the alternative allele. These criteria were implemented with the “filter” module of BCFtools v1.7 [20] using parameters detailed in Supplementary Table 12.

From these filtered VCFs, evaluation metrics were calculated as detailed below.

### Evaluating SNP-calling pipelines using real sequencing data

Parallel sets of 150-bp Illumina HiSeq 4000 paired-end short reads and ONT long reads were obtained from 16 environmentally sourced samples from the REHAB project (“the environmental REsistome: confluence of Human and Animal Biota in antibiotic resistance spread” [124]), as detailed in [63]: 4 *Enterobacter* spp., 4 *Klebsiella* spp., 4 *Citrobacter* spp., and 4 *Escherichia coli*, with species identified using matrix-assisted laser desorption ionization (MALDI) time-of-flight mass spectrometry, plus subcultures of stocks of 2 reference strains, *K. pneumoniae* subsp. *pneumoniae* MGH 78,578 and *E. coli* CFT073. Additional predictions were made using both the protein- and nucleotide-level classification tools Kaiju v1.6.1 [88] and Kraken2 v2.0.7 (Kraken, RRID:SCR_005484) [125], respectively. Kaiju was used with 2 databases, 1 broad and 1 deep, both created on 5 February 2019: “P” ([126]; >20 million bacterial and archaeal genomes from the compact, manually curated, EMBL proGenomes [127], supplemented by ∼10,000 viral genomes from NCBI RefSeq [128]) and “E” ([129]; >100 million bacterial, archaeal, viral, and fungal genomes from NCBI nr, alongside various microbial eukaryotic taxa). Kaiju was run with parameters -e 5 and -E 0.05, which, respectively, allow 5 mismatches per read and filter results on the basis of an E-value threshold of 0.05. The read classifications from both databases were integrated using the Kaiju “mergeOutputs” module, which adjudicates on the basis of the lowest taxonomic rank of each pair of classifications, provided they are within the same lineage, or else reclassifies the read at the lowest common taxonomic rank ancestral to the two. Kraken2 was run with default parameters using the MiniKraken2 v1 database ([130], created 12 October 2018), which was built from the complete set of NCBI RefSeq bacterial, archaeal, and viral genomes.

Hybrid assemblies were produced using methods detailed in [63] and briefly recapitulated here. Illumina reads were processed using COMPASS (see above). ONT reads were adapter-trimmed using Porechop v0.2.2 [131] with default parameters, and then error-corrected and subsampled (preferentially selecting the longest reads) to 30–40× coverage using Canu v1.5 (Canu, RRID:SCR_015880) [132] with default parameters. Finally, Illumina-ONT hybrid assemblies for each genome were generated using Unicycler v0.4.0 [57] with default parameters. The original study found high agreement between these assemblies and those produced using hybrid assembly with PacBio long reads rather than ONT, giving us high confidence in their robustness.

In the simulated datasets, SNPs are introduced *in silico* into a genome, with reads containing these SNPs then simulated from it. With this dataset, however, there are no SNPs within each genome: we have only the short reads (i.e., real output from an Illumina sequencer) and the genome assembled from them (with which there is an expectation of near-perfect read mapping).

To evaluate pipeline performance when the reads are aligned to a divergent genome, reference genomes were selected as representative of the predicted species, with distances between the 2 calculated using Mash v2.1 [60] and spanning approximately equal intervals from 0.01 to 0.12 (representative genomes and Mash distances are detailed in Supplementary Table 8). The truth set of SNPs between the representative genome and each hybrid assembly was the intersection of nucmer and Parsnp calls, as above.

Samples, source locations, MALDI ID scores, and associated species predictions are detailed in Supplementary Table 8. Raw sequencing data have been deposited with the NCBI under BioProject accession PRJNA422511 [133], with the associated hybrid assemblies available via FigShare [134].

To allow both the replication and expansion of this evaluation using real sequencing data, a complete archive is available as Supplementary Dataset 2 (hosted online via the Oxford Research Archive [135]) comprising reads, assemblies, indexed reference genomes, the associated SNP call truth sets, VCFs, and a suite of Perl scripts.

### Evaluation metrics

For each pipeline, we calculated the absolute number of TP (the variant is in the simulated genome and correctly called by the pipeline), FP (the pipeline calls a variant that is not in the simulated genome), and FN SNP calls (the variant is in the simulated genome but the pipeline does not call it). We did not calculate true-negative calls for 2 reasons. First, to do so requires a VCF containing calls for all sites, a function offered by some variant callers (such as mpileup) but not all. Second, when aligning reads to a divergent genome, a disproportionately large number of reference sites will be excluded, particularly in more diverse species (e.g., gene numbers in *N. gonorrhoeae* differ by up to one-third; see Supplementary Table 5).

We then calculated the precision (positive predictive value) of each pipeline as TP/(TP + FP), recall (sensitivity) as TP/(TP + FN), miss rate as FN/(TP + FN), and total number of errors (FP + FN) per million sequenced bases. We did not calculate specificity because this depends on true-negative calls. We also calculated the F-score (as in [11]), which considers precision and recall with equal weight: F = 2 * [(precision * recall)/(precision + recall)]. The F-score evaluates each pipeline as a single value bounded between 0 and 1 (perfect precision and recall). We also ranked each pipeline on the basis of each metric so that—for example—the pipeline with the highest F-score, and the pipeline with the lowest number of FPs, would be rank 1 in their respective distributions. As an additional “overall performance” measure, we calculated the sum of ranks for the 7 core evaluation metrics (the absolute numbers of TP, FP, and FN calls, and the proportion-based precision, recall, F-score, and total error rate per million sequenced bases). Pipelines with a lower sum of ranks would, in general, have higher overall performance.

We note that when SNPs are called after aligning reads from 1 strain to that of a divergent strain, the SNP-calling pipeline will call positions for both the truth set of strain-specific *in silico* SNPs and any inter-strain variants. To allow a comparable evaluation of pipelines in this circumstance, inter-strain calls (obtained using nucmer and Parsnp; see above) are discarded and not explicitly considered either TP, FP, or FN. While the set of true SNPs when aligning to a divergent strain will be smaller than that when aligned to the same strain (because all SNPs are simulated in genic regions but not all genes are shared between strains), this will not affect proportion-based evaluation metrics, such as F-score.

### Effect size of differences in the F-score distribution between pipelines

Differences between distributions are assessed by Mann-Whitney *U* tests, with results interpreted using the non-parametric effect size estimator Cliff delta [61, 62], estimated at a confidence level of 95% using the R package effsize v0.7.1 [136]. The Cliff delta employs the concept of dominance (which refers to the degree of overlap between distributions) and so is more robust when distributions are skewed. Estimates of delta are bound in the interval (−1, 1), with extreme values indicating a lack of overlap between groups (respectively, set 1 ≪ set 2 and set 1 ≫ set 2). Distributions with |delta| < 0.147 are negligibly different, as in [137]. Conversely, distributions with |delta| ≥ 0.60 are considered to have large differences.

## Availability of Supporting Data and Materials

All data analysed during this study are included in this published article and its supplementary information files. The simulated datasets generated during this study—comprising the SNP-containing genomes, log files of the SNPs introduced into each genome, and VCFs of strain–to–representative genome SNP calls—are available in Supplementary Dataset 1 (hosted online via the Oxford Research Archive at http://dx.doi.org/10.5287/bodleian:AmNXrjYN8).

Raw sequencing data and assemblies from the REHAB project, described in [63], are available in the NCBI under BioProject accession PRJNA42251 (https://www.ncbi.nlm.nih.gov/bioproject/PRJNA422511), with associated hybrid assemblies available via FigShare [134].

A complete archive to facilitate both the replication and expansion of this evaluation using the real (REHAB project) sequencing data is available as Supplementary Dataset 2 (hosted online via the Oxford Research Archive at https://tinyurl.com/v4p6vol). This archive comprises 18 sets of paired-end reads and assemblies, the associated indexed reference genomes, SNP call truth sets, VCFs, and a suite of Perl scripts. These scripts are also available via https://github.com/oxfordmmm/GenomicDiversityPaper. Snapshots of these data and code are also available from the *GigaScience* GigaDB repository [138].

## Availability of Supporting Source Code and Requirements

Project name: Genomic diversity affects the accuracy of bacterial SNP calling pipelines

Project home page: https://github.com/oxfordmmm/GenomicDiversityPaper

Operating system(s): Platform-independent

Programming language: Perl (v5.22.1)

Other requirements: Third-party software prerequisites are detailed in documentation provided with Supplementary Dataset 2 (https://tinyurl.com/v4p6vol)

License: GNU GPL

## Additional Files

**Supplementary Table 1**. Sources of software

**Supplementary Table 2**. Genomes into which SNPs were introduced *in silico*, and various measures of distance between each strain's genome and the representative genome of that species

**Supplementary Table 3**. Summary statistics of SNP-calling pipelines after aligning simulated reads to the same reference genome as their origin

**Supplementary Table 4**. Ranked performance of SNP-calling pipelines after aligning simulated reads to the same reference genome as their origin

**Supplementary Table 5**. Genome size diversity within 5 clinically common bacterial species

**Supplementary Table 6**. Summary statistics of SNP-calling pipelines after aligning simulated reads to a reference genome differing from their origin

**Supplementary Table 7**. Ranked performance of SNP-calling pipelines after aligning simulated reads to reference genome differing from their origin

**Supplementary Table 8**. Environmentally sourced/reference gram-negative isolates and associated representative genomes.

**Supplementary Table 9**. Summary statistics of SNP-calling pipelines after aligning real reads to a reference genome differing from their origin

**Supplementary Table 10**. Ranked performance of SNP-calling pipelines after aligning real reads to reference genome differing from their origin

**Supplementary Table 11**. Proportion of strain-specific *in silico* SNPs detected in whole-genome alignments between the strain genome and a representative genome

**Supplementary Table 12**. VCF filtering parameters, as used by BCFtools

**Supplementary Table 13**. Summary statistics of SNP-calling pipelines after aligning both simulated error-free and error-containing reads to the same reference genome as their origin

**Supplementary Table 14**. Summary statistics of SNP-calling pipelines after aligning both simulated error-free and error-containing reads to a reference genome differing from their origin

**Supplementary Table 15**. Summary statistics of SNP-calling pipelines after aligning simulated error-free reads to a reference genome differing from their origin, both with and without local indel realignment

**Supplementary Table 16**. Summary statistics of *E. coli* SNP-calling pipelines after aligning simulated error-free reads to a reference genome differing from their origin, both with and without VCF regularization

**Supplementary Table 17**. Summary statistics of *E. coli* SNP-calling pipelines after aligning simulated error-free reads to a reference genome differing from their origin, at 5-, 10-, 25- and 50-fold depths of coverage

**Supplementary Figure 1**. Reduced performance of SNP-calling pipelines with increasing genetic distance between the reads and the reference genome (assayed as total number of SNPs). The median F-score across a set of 41 pipelines, per strain, decreases as the distance between the strain and the reference genome increases (assayed as the total number of SNPs between the strain and representative genome, i.e., the set of strain-specific *in silico* SNPs plus inter-strain SNPs). Each point indicates the genome of 1 strain per species (n = 254 strains). Points are coloured by the species of each strain (n = 10 species). Summary statistics for each pipeline are given in Supplementary Table 6, performance ranks in Supplementary Table 7, and the genetic distance between strains in Supplementary Table 2. Quantitatively similar results are seen if assaying distance as the Mash distance, which is based on the proportion of *k*-mers shared between genomes (Fig. 3).

**Supplementary Figure 2**. Decreasing sensitivity (i.e., an increased number of false-negative calls) with increasing genetic distance between the reads and the reference genome (assayed as Mash distance). The median sensitivity (recall) across a set of 41 pipelines, per strain, increases as the distance between the strain and the reference genome increases (assayed as the Mash distance, which is based on the proportion of shared *k*-mers between genomes). Each point indicates the genome of 1 strain per species (n = 254 strains). Points are coloured by the species of each strain (n = 10 species). Summary statistics for each pipeline are given in Supplementary Table 6, performance ranks in Supplementary Table 7, and the genetic distance between strains in Supplementary Table 2.

**Supplementary Figure 3**. Total number of SNPs it is possible to call should reads from 1 strain be aligned to a representative genome of that species. Strong correlation between the total number of SNPs introduced *in silico* into 1 genome and the maximum number of SNPs it is possible to call assuming reads from the former are aligned to a representative genome of that species (which will not necessarily contain the same complement of genes). Each point represents the genome of 1 strain, with genomes detailed in Supplementary Table 2. The line y = x is shown in red.

**Supplementary Figure 4**. Head-to-head performance comparison of all pipelines relative to Snippy, on the basis of F-score, using simulated data. This figure directly compares the performance, using simulated data, of 40 pipelines relative to Snippy. Each point indicates the median F-score for the genome of 1 strain per species (n = 254 strains). Data for Snippy are plotted on the x-axis, and for the named pipeline on the y-axis. Raw data for this figure are given in Supplementary Table 6. Text in the top left of each panel is an interpretation of the difference between each pair of distributions, obtained using the R package “effsize,” which applies the non-parametric effect size estimator Cliff delta to the results of a Mann-Whitney *U* test.

**Supplementary Figure 5**. Selection of *E. coli* isolates by manual review of dendrogram topology. There are numerous usable complete genomes for *E. coli*. For the SNP-calling evaluation, a subset of isolates was selected (indicated in red boxes) so as to maximize the diversity of clades represented. To do so, an all-against-all distance matrix for each genome was created using the “triangle” component of Mash v2.1, with a dendrogram constructed using the neighbour-joining method implemented in MEGA v7.0.14. Sources for the selected genomes are given in Supplementary Table 2.

**Supplementary Figure 6**. Selection of *K. pneumoniae* isolates by manual review of dendrogram topology. There are numerous usable complete genomes for *K. pneumoniae*. For the SNP-calling evaluation, a subset of isolates was selected (indicated in red boxes) so as to maximize the diversity of clades represented. To do so, an all-against-all distance matrix for each genome was created using the “triangle” component of Mash v2.1, with a dendrogram constructed using the neighbour-joining method implemented in MEGA v7.0.14. Sources for the selected genomes are given in Supplementary Table 2.

**Supplementary Figure 7**. Selection of *L. monocytogenes* isolates by manual review of dendrogram topology. There are numerous usable complete genomes for *L. monocytogenes*. For the SNP-calling evaluation, a subset of isolates was selected (indicated in red boxes) so as to maximize the diversity of clades represented. To do so, an all-against-all distance matrix for each genome was created using the “triangle” component of Mash v2.1, with a dendrogram constructed using the neighbour-joining method implemented in MEGA v7.0.14. Sources for the selected genomes are given in Supplementary Table 2.

**Supplementary Figure 8**. Selection of *S. enterica* isolates by manual review of dendrogram topology. There are numerous usable complete genomes for *S. enterica*. For the SNP-calling evaluation, a subset of isolates was selected (indicated in red boxes) so as to maximize the diversity of clades represented. To do so, an all-against-all distance matrix for each genome was created using the “triangle” component of Mash v2.1, with a dendrogram constructed using the neighbour-joining method implemented in MEGA v7.0.14. Sources for the selected genomes are given in Supplementary Table 2.

**Supplementary Figure 9**. Selection of *S. aureus* isolates by manual review of dendrogram topology. There are numerous usable complete genomes for *S. aureus*. For the SNP-calling evaluation, a subset of isolates was selected (indicated in red boxes) so as to maximize the diversity of clades represented. To do so, an all-against-all distance matrix for each genome was created using the “triangle” component of Mash v2.1, with a dendrogram constructed using the neighbour-joining method implemented in MEGA v7.0.14. Sources for the selected genomes are given in Supplementary Table 2.

**Supplementary Dataset 1**. Simulated datasets for evaluating bacterial SNP-calling pipelines. This archive contains the set of 254 SNP-containing genomes, VCFs containing the nucmer and Parsnp strain–to–representative genome SNP calls, and the final truth sets of SNPs used for evaluation.

**Supplementary Dataset 2**. Real sequencing datasets for evaluating bacterial SNP-calling pipelines. This is a complete archive to facilitate both the replication and expansion of this evaluation using real (REHAB project) sequencing data. It comprises 18 sets of paired-end reads and assemblies, the associated indexed reference genomes, SNP call truth sets, VCFs, and a suite of Perl scripts.

## Abbreviations

BWA: Burrows-Wheeler Aligner; COMPASS: Complete Pathogen Sequencing Solution; EMBL: European Molecular Biology Laboratory; FN: false negative; FP: false positive; GASSST: Global Alignment Short Sequence Search Tool; GATK: Genome Analysis Toolkit; GFF: General Feature Format; MALDI: matrix-assisted laser desorption ionization; NCBI: National Center for Biotechnology Information; NHS: National Health Service; ONT: Oxford Nanopore Technologies; SNAP: Semi-HMM-based Nucleic Acid Parser; SNP: single-nucleotide polymorphism; TP: true positive; VCF: variant call format.

## Competing Interests

The authors declare that they have no competing interests.

## Supplementary Material

giaa007_GIGA-D-19-00189_Original_Submission

giaa007_GIGA-D-19-00189_Revision_1

giaa007_GIGA-D-19-00189_Revision_2

giaa007_Response_to_Reviewer_Comments_Original_Submission

giaa007_Response_to_Reviewer_Comments_Revision_1

giaa007_Reviewer_1_Report_Original_Submission Jason Sahl -- 7/15/2019 Reviewed

giaa007_Reviewer_1_Report_Revision_1 Jason Sahl -- 12/17/2019 Reviewed

giaa007_Reviewer_2_Report_Original_Submission C. Titus Brown -- 9/29/2019 Reviewed

giaa007_Supplement_Files
