# Supplementary material for: Genomic diversity affects the accuracy of bacterial single-nucleotide polymorphism–calling pipelines
Source: Gigascience. 2020 Feb 6;9(2):giaa007. doi: 10.1093/gigascience/giaa007 (PMC7002876; doi:10.1093/gigascience/giaa007)
Supplement: giaa007_GIGA-D-19-00189_Revision_2 [file giaa007_giga-d-19-00189_revision_2.pdf]

GigaScience

Genomic diversity affects the accuracy of bacterial SNP calling pipelines

--Manuscript Draft--

**Manuscript Number:**	GIGA-D-19-00189R2
**Full Title:**	Genomic diversity affects the accuracy of bacterial SNP calling pipelines
**Article Type:**	Research
**Funding Information:**	National Institute for Health Research Health Protection Research Unit (HPRU-2012-10041) — Not applicable<br>Antimicrobial Resistance Cross Council Initiative (NE/N019989/1) — Dr Liam P. Shaw<br>Biotechnology and Biological Sciences Research Council (GB) (BB/P013740/1) — Not applicable
**Abstract:**	Background<br>Accurately identifying SNPs from bacterial sequencing data is an essential requirement for using genomics to track transmission and predict important phenotypes such as antimicrobial resistance. However, most previous performance evaluations of SNP calling have been restricted to eukaryotic (human) data. Additionally, bacterial SNP calling requires choosing an appropriate reference genome to align reads to, which, together with the bioinformatic pipeline, affects the accuracy and completeness of a set of SNP calls obtained.<br>This study evaluates the performance of 209 SNP calling pipelines using a combination of simulated data from 254 strains of 10 clinically common bacteria and real data from environmentally-sourced and genomically diverse isolates within the genera Citrobacter, Enterobacter , Escherichia and Klebsiella .<br><br>Results<br>We evaluated the performance of 209 SNP calling pipelines, aligning reads to genomes of the same or a divergent strain. Irrespective of pipeline, a principal determinant of reliable SNP calling was reference genome selection. Across multiple taxa, there was a strong inverse relationship between pipeline sensitivity and precision, and the Mash distance (a proxy for average nucleotide divergence) between reads and reference genome. The effect was especially pronounced for diverse, recombinogenic, bacteria such as Escherichia coli , but less dominant for clonal species such as Mycobacterium tuberculosis .<br><br>Conclusions<br>The accuracy of SNP calling for a given species is compromised by increasing intra-species diversity. When reads were aligned to the same genome from which they were sequenced, among the highest performing pipelines was Novoalign/GATK. By contrast, when reads were aligned to particularly divergent genomes, the highest-performing pipelines often employed the aligners NextGenMap or SMALT, and/or the variant callers LoFreq, mpileup or Strelka.
**Corresponding Author:**	Stephen J Bush<br><br>UNITED KINGDOM
**Corresponding Author Secondary Information:**	
**Corresponding Author's Institution:**	
**Corresponding Author's Secondary Institution:**	
**First Author:**	Stephen J Bush
**First Author Secondary Information:**	
**Order of Authors:**	Stephen J Bush<br>Dona Foster

Powered by Editorial Manager® and ProduXion Manager® from Aries Systems Corporation

	David W. Eyre
	Emily L. Clark
	Nicola De Maio
	Liam P. Shaw
	Nicole Stoesser
	Tim E. A. Peto
	Derrick W. Crook
	A. Sarah Walker
**Order of Authors Secondary Information:**	
**Response to Reviewers:**	Response to reviewers<br><br>For the Perl scripts we would recommend to put these in a code repository and include a software section at the end of the paper that is structured as follows:<br><br>Availability of supporting source code and requirements<br>Project name: e.g. My bioinformatics project<br>Project home page: e.g. https://github.com/ISA-tools<br>Operating system(s): e.g. Platform independent<br>Programming language: e.g. Java<br>Other requirements: e.g. Java 1.3.1 or higher, Tomcat 4.0 or higher<br>License: e.g. GNU GPL, FreeBSD etc.<br>RRID: if applicable, e.g. RRID: SCR_014986 (see below)<br><br>Response: we have added the Perl scripts to a GitHub repository, https://github.com/oxfordmmm/GenomicDiversityPaper, now referred to on line 1130. Lines 1133-1141 specify the requirements:<br>Project name: “Genomic diversity affects the accuracy of bacterial SNP calling pipelines”<br>Project home page: https://github.com/oxfordmmm/GenomicDiversityPaper<br>Operating system(s): platform-independent<br>Programming language: Perl (v5.22.1)<br>Other requirements: third-party software prerequisites are detailed in documentation provided with Supplementary Dataset 2 (https://ora.ox.ac.uk/objects/uuid:8f902497-955e-4b84-9b85-693ee0e4433e).<br>License: GNU GPL.<br><br>Reviewer reports:<br>Reviewer #1: The authors did a good job at addressing my previous comments as well as expanding the analyses to cover a more diverse suite of tools. The authors still use 'pipeline' to sometimes describe an aligner/variant caller and also an all-in-one method, which may cause confusion, but is ultimately their decision. The authors still mention Snippy as one of the best performing tools, which seems odd considering the performance in Supplementary Table 10 using real data. Perhaps the authors could state that snippy did well on simulated data, while other tools performed better on real data. The captions on the supplementary tables could also be updated to differentiate between simulated and real data.<br><br>Response: we removed from the abstract (line 47) the statement that “across the full range of genomes, among the consistently highest performing pipelines was Snippy” as this conclusion was drawn from its performance across both simulated and real datasets, when n=41 pipelines. However, with the expansion of the number of pipelines to 209, and the testing of these additional pipelines only on real data, we sought to keep the conclusions drawn based on real data distinct from those based on simulated data. To that end, we also amended line 549 to read “Nevertheless, Snippy, which employs Freebayes, is particularly robust to this, being among the most sensitive pipelines when evaluated using simulated data (Figure 5 and Supplementary Figure 4).” We have also amended the titles of Figure 5 and Supplementary Figure 4, and Supplementary Tables 3, 4, 6, 7, 13, 14, 15, 16 and 17 to emphasise their use of simulated data (the supplementary tables containing results from real data, numbers 9

Powered by Editorial Manager® and ProduXion Manager® from Aries Systems Corporation

	and 10, were already so labelled).<br><br>Additionally, the authors include an analysis that masks repeats using BLAST. However, the thresholds chosen for BLAST will likely only mask very similar paralogs, while the more divergent paralogs are expected to have a greater impact on mis-mapping and variant discovery (this could just be a discussion point).<br><br>Response: we agree that the parameters used for repeat-masking are especially important and have expanded the discussion to include this. We have added, at line 377: “it is important to note that the parameters used for repeat-masking will determine which paralogues will be successfully masked. For the purpose of this study, we used reasonably conservative parameters (detailed in Supplementary Text 1) and so expect to have primarily masked more similar paralogues. The likelihood of mis-mapping (and thereby false positive SNP calling) would increase among more divergent paralogues, although optimising parameters to detect these is non-trivial. More lenient repeat-masking parameters, in masking more divergent positions, would also reduce the number of true SNPs it is possible to call.” This has also been added to the supplementary text, at lines 680-686.<br><br>Some additional thoughts that may improve the manuscript:<br><br>L306: The authors should mention that they also now include 2 additional "all-in-one" pipelines<br><br>Response: we have revised the sentence to read “we next expanded the scope of the evaluation to 209 pipelines (representing the addition of 12 aligners, 4 callers, and 2 ‘all-in-one’ pipelines, SpeedSeq and SPANDx)…”<br><br>L1127-1128: Please check this link. I received a 404 error when I tried to access it. The link in the response to reviewers did work for me<br><br>Response: I’m afraid we can’t replicate this 404 – we’ve re-checked the link (https://ora.ox.ac.uk/objects/uuid:8f902497-955e-4b84-9b85-693ee0e4433e) and do find it accessible.<br><br>Figure 7: The x-axis labels don't line up with the bars, which makes it difficult to interpret. Would staggering the labels between the top and bottom of the graph help with this?<br><br>Response: we have re-drawn with Figure 7 with better-positioned x-axis labels.
**Additional Information:**	
**Question**	**Response**
Are you submitting this manuscript to a special series or article collection?	No
**Experimental design and statistics**<br><br>Full details of the experimental design and statistical methods used should be given in the Methods section, as detailed in our Minimum Standards Reporting Checklist. Information essential to interpreting the data presented should be made available in the figure legends.<br><br>Have you included all the information requested in your manuscript?	Yes

Powered by Editorial Manager® and ProduXion Manager® from Aries Systems Corporation

**Resources**<br><br>A description of all resources used, including antibodies, cell lines, animals and software tools, with enough information to allow them to be uniquely identified, should be included in the Methods section. Authors are strongly encouraged to cite Research Resource Identifiers (RRIDs) for antibodies, model organisms and tools, where possible.<br><br>Have you included the information requested as detailed in our Minimum Standards Reporting Checklist?	Yes
**Availability of data and materials**<br><br>All datasets and code on which the conclusions of the paper rely must be either included in your submission or deposited in publicly available repositories (where available and ethically appropriate), referencing such data using a unique identifier in the references and in the “Availability of Data and Materials” section of your manuscript.<br><br>Have you have met the above requirement as detailed in our Minimum Standards Reporting Checklist?	Yes

Manuscript Click here to access/download;Manuscript;Full Manuscript.docx

Genomic diversity affects the accuracy of bacterial SNP calling pipelines

Stephen J. Bush[1,2 *], Dona Foster[1,3], David W. Eyre[1], Emily L. Clark[4], Nicola De Maio[5], Liam P. Shaw[1], Nicole Stoesser[1], Tim E. A. Peto[1,2,3], Derrick W. Crook[1,2,3], A. Sarah Walker[1,2,3]

[1] Nuffield Department of Medicine, University of Oxford, Oxford, UK
[2] National Institute for Health Research Health Research Protection Unit in Healthcare Associated Infections and Antimicrobial Resistance at University of Oxford in partnership with Public Health England, Oxford, UK
[3] National Institute for Health Research Oxford Biomedical Research Centre, Oxford, UK
[4] The Roslin Institute and Royal (Dick) School of Veterinary Studies, University of Edinburgh, Edinburgh, UK
[5] European Molecular Biology Laboratory, European Bioinformatics Institute (EMBL-EBI), Wellcome Genome Campus, Hinxton, Cambridgeshire, CB10 1SH
* corresponding author

ORCID IDs
Stephen Bush: 0000-0001-9341-2562; David W. Eyre: 0000-0001-5095-6367; Nicola De Maio: 0000-0002-1776-8564; Liam P. Shaw: 0000-0001-7332-0820; Nicole Stoesser: 0000-0002-4508-7969; Derrick W. Crook: 0000-0002-0590-2850

Abstract

Background
Accurately identifying SNPs from bacterial sequencing data is an essential requirement for using genomics to track transmission and predict important phenotypes such as antimicrobial resistance. However, most previous performance evaluations of SNP calling have been restricted to eukaryotic (human) data. Additionally, bacterial SNP calling requires choosing an appropriate reference genome to align reads to, which, together with the bioinformatic pipeline, affects the accuracy and completeness of a set of SNP calls obtained.
This study evaluates the performance of 209 SNP calling pipelines using a combination of simulated data from 254 strains of 10 clinically common bacteria and real data from environmentally-sourced and genomically diverse isolates within the genera *Citrobacter*, *Enterobacter*, *Escherichia* and *Klebsiella*.

Results

We evaluated the performance of 209 SNP calling pipelines, aligning reads to genomes of the same or a divergent strain. Irrespective of pipeline, a principal determinant of reliable SNP calling was reference genome selection. Across multiple taxa, there was a strong inverse relationship between pipeline sensitivity and precision, and the Mash distance (a proxy for average nucleotide divergence) between reads and reference genome. The effect was especially pronounced for diverse, recombinogenic, bacteria such as *Escherichia coli*, but less dominant for clonal species such as *Mycobacterium tuberculosis*.

Conclusions

The accuracy of SNP calling for a given species is compromised by increasing intra-species diversity. When reads were aligned to the same genome from which they were sequenced, among the highest performing pipelines was Novoalign/GATK. By contrast, when reads were aligned to particularly divergent genomes, the highest-performing pipelines often employed the aligners NextGenMap or SMALT, and/or the variant callers LoFreq, mpileup or Strelka.

Introduction

Accurately identifying single nucleotide polymorphism (SNPs) from bacterial DNA is essential for monitoring outbreaks (as in [1, 2]) and predicting phenotypes, such as antimicrobial resistance [3], although the pipeline selected for this task strongly impacts the outcome [4]. Current bacterial sequencing technologies generate short fragments of DNA sequence (‘reads’) from which the bacterial genome can be reconstructed. Reference-based mapping approaches use a known reference genome to guide this process, using a combination of an aligner, which identifies the location in the genome each read is likely to have arisen from, and a variant caller, which summarises the available information at each site to identify variants including SNPs and indels (see reviews for an overview of alignment [5, 6] and SNP calling [7] algorithms). This evaluation focuses only on SNP calling; we did not evaluate indel calling as this can require different algorithms (see review [8]).
The output from different aligner/caller combinations is often poorly concordant. For example, up to 5% of SNPs are uniquely called by one of five different pipelines [9] with even lower agreement upon structural variants [10].

Although a mature field, systematic evaluations of variant calling pipelines are often limited to eukaryotic data, usually human [11-15] but also *C. elegans* [16] and dairy cattle [17] (see also review [18]). This is because truth sets of known variants, such as the Illumina Platinum Genomes [19], are relatively few in number and human-centred, being expensive to create and biased toward the methods that produced them [20]. As such, to date, bacterial SNP calling evaluations are comparatively limited in scope (for example, comparing 4 aligners with 1 caller, mpileup [21], using *Listeria monocytogenes* [22]).

Relatively few truth sets exist for bacteria and so the choice of pipeline for bacterial SNP calling is often informed by performance on human data. Many evaluations conclude in favour of the publicly-available BWA-mem [23] or commercial Novoalign (www.novocraft.com) as choices of aligner, and GATK [24, 25] or mpileup as variant callers, with recommendations for a default choice of pipeline, independent of specific analytic requirements, including Novoalign followed by GATK [26], and BWA-mem followed by either mpileup [14], GATK [12], or VarDict [11].

This study evaluates a range of SNP calling pipelines across multiple bacterial species, both when reads are sequenced from and aligned to the same genome, and when reads are aligned to a representative genome of that species.

SNP calling pipelines are typically constructed around a read aligner (which takes FASTQ as input and produces BAM as output) and a variant caller (which takes BAM as input and produces VCF as output), often with several pre- and post-processing steps (for instance, cleaning a raw FASTQ prior to alignment, or filtering a BAM prior to variant calling). For the purpose of this study, when evaluating the two core components of aligner and caller, we use ‘pipeline’ to mean ‘an aligner/caller combination, with all other steps in common.’

In order to cover a broad range of methodologies (see review for an overview of the different algorithmic approaches [27]), we assessed the combination of 16 short read aligners (BBMap (https://sourceforge.net/projects/bbmap/), Bowtie2 [28], BWA-mem and BWA-sw [23], Cushaw3 [29], GASSST [30], GEM [31], HISAT2 [32], minimap2 [33], MOSAIK [34], NextGenMap [35], Novoalign, SMALT (http://www.sanger.ac.uk/science/tools/smalt-0), SNAP [36], and Stampy [37] (both with and without pre-alignment with BWA-aln), and Yara [38]) used in conjunction with 14 variant callers (16GT [39], DeepVariant [40], Freebayes

[41], GATK HaplotypeCaller [24, 25], LoFreq [42], mpileup [21], Octopus [43], Pilon [44], Platypus [45], SolSNP (http://sourceforge.net/projects/solsnp/), SNVer [46], SNVSniffer [47], Strelka [48] and VarScan [49]). We also evaluated three 'all-in-one' variant calling pipelines, Snippy (https://github.com/tseemann/snippy), SPANDx [50] and SpeedSeq [51], which consolidate various open-source packages into one tool. Reasons for excluding other programs are detailed in Supplementary Text 1. Where possible, we applied a common set of pre- or post-processing steps to each aligner/caller combination, although note that these could differ from those applied within an 'all-in-one' tool (discussed further in Supplementary Text 1).

Benchmarking evaluations are, however comprehensive, ephemeral. As programs are being constantly created and updated, it will always be possible to expand the scope of any evaluation. To that end, this study originally assessed an initial subset of 41 pipelines, the combination of 4 aligners (BWA-mem, minimap2, Novoalign, and Stampy) and 10 variant callers (the aforementioned list, excluding DeepVariant, Octopus, Pilon, and SolSNP), plus Snippy.

To evaluate each of this initial set of 41 pipelines, we simulated 3 sets of 150bp and 3 sets of 300bp reads (characteristic of the Illumina NextSeq and MiSeq platforms, respectively) at 50-fold depth from 254 strains of 10 clinically common species (2 to 36 strains per species), each with fully sequenced (closed) core genomes: the Gram-positive *Clostridioides difficile* (formerly *Clostridium difficile* [52]), *Listeria monocytogenes*, *Staphylococcus aureus*, and *Streptococcus pneumoniae* (all Gram-positive), *Escherichia coli*, *Klebsiella pneumoniae*, *Neisseria gonorrhoeae*, *Salmonella enterica*, and *Shigella dysenteriae* (all Gram-negative), and *Mycobacterium tuberculosis*. For each strain, we evaluated all pipelines using two different genomes for alignment: one being the same genome from which the reads were simulated, and one being the NCBI 'reference genome', a high-quality (but essentially arbitrary) representative of that species, typically chosen on the basis of assembly and annotation quality, available experimental support, and/or wide recognition as a community standard (such as *C. difficile* 630, the first sequenced strain for that species [53]). We added approximately 8000-25,000 SNPs *in silico* to each genome, equivalent to 5 SNPs per genic region, or 1 SNP per 60-120 bases.

While simulation studies can offer useful insight, they can be sensitive to the specific details of the simulations. Therefore, we also evaluated performance on real data to verify our conclusions. We used 16 environmentally-sourced and genomically diverse Gram-negative species of the genera *Citrobacter, Enterobacter, Escherichia* and *Klebsiella*, along with two reference strains, from which closed hybrid *de novo* assemblies were previously generated using both Illumina (short) and ONT (long; Oxford Nanopore Technologies) reads [54]. For this aspect of the study, we quintupled the scope of the evaluation from the initial set of 41 pipelines and also present results for a larger set of 209 pipelines.

All pipelines aim to call variants with high specificity (i.e. a high proportion of non-variant sites in the truth set are correctly identified as the reference allele by the pipeline) and high sensitivity (i.e. a high proportion of true SNPs are found by the pipeline). The optimal trade-off between these two properties may vary depending on the application. For example, in transmission inference, minimising false positive SNP calls (i.e. high specificity), is likely to be most important, whereas high sensitivity may be more important when identifying variants associated with antibiotic resistance. We therefore report detailed performance metrics for all pipelines, including recall (sensitivity), precision (positive predictive value, the proportion of SNPs identified that are true SNPs), and the F-score, the harmonic mean of precision and recall [55].

Results

Evaluating SNP calling pipelines when the genome for alignment is also the source of the reads

The performance of 41 SNP calling pipelines (Supplementary Table 1) was first evaluated using reads simulated from 254 closed bacterial genomes (Supplementary Table 2), as illustrated in Figure 1. In order to exclude biases introduced during other parts of the workflow, such as DNA library preparation and sequencing error, reads were simulated error-free. There was negligible difference in performance when reads were simulated with sequencing errors (see Supplementary Text 1).

This dataset contains 62,484 VCFs (comprising 2 read lengths [150 and 300bp] * 3 replicates * 254 genomes * 41 pipelines). The number of reads simulated from each species and the performance statistics for each pipeline – the number of true positives (TP), false positives

(FP) and false negatives (FN), precision, recall, F-score, and total number of errors (i.e. FP + FN) per million sequenced bases – are given in Supplementary Table 3, with the distribution of F-scores illustrated in Figure 2A.

Median F-scores were over 0.99 for all but four aligner/callers with small interquartile ranges (approx. 0.005), although outliers were nevertheless notable (Figure 2A), suggesting that reference genome can affect performance of a given pipeline.

Table 1 shows the top ranked pipelines averaged across all species' genomes, based on 7 different performance measures and on the sum of their ranks (which constitutes an 'overall performance' measure, lower values indicating higher overall performance). Supplementary Table 4 shows the sum of ranks for each pipeline per species, with several variant callers consistently found among the highest-performing (Freebayes and GATK) and lowest-performing pipelines (16GT and SNVSniffer), irrespective of aligner.

If considering performance across all species, Novoalign/GATK had the highest median F-score (0.994), lowest sum of ranks (10), the lowest number of errors per million sequenced bases (0.944), and the largest absolute number of true positive calls (15,778) (Table 1). However, in this initial simulation, as the reads are error-free and the reference genome is the same as the source of the reads, many pipelines avoid false positive calls and report a perfect precision of 1.

Evaluating SNP calling pipelines when the genome for alignment diverges from the source of the reads

Due to the high genomic diversity of some bacterial species, the appropriate selection of reference genomes is non-trivial. To assess how pipeline performance is affected by divergence between the source and reference genomes, SNPs were re-called after mapping all reads to a single representative genome for that species (illustrated in Figure 1). To identify true variants, closed genomes were aligned against the representative genome using both nucmer [56] and Parsnp [57], with consensus calls identified within one-to-one alignment blocks (see Methods). Estimates of the distance between each genome and the representative genome are given in Supplementary Table 2, with the genomic diversity of each species summarised in Supplementary Table 5. We quantified genomic distances using the Mash distance, which reflects the proportion of k-mers shared between a pair of genomes as a

proxy for average nucleotide divergence [58]. The performance statistics for each pipeline are shown in Supplementary Table 6, with an associated ranked summary in Supplementary Table 7.

In general, aligning reads from one strain to a divergent reference leads to a decrease in median F-score and increase in interquartile range of the F-score distribution, with pipeline performance more negatively affected by choice of aligner than caller (Figure 2B).

Although across the full range of genomes, many pipelines show comparable performance (Figure 2B), there was a strong negative correlation between the Mash distance and F-score (Spearman's *rho* = -0.72, $p < 10^{-15}$; Figure 3). The negative correlation between F-score and the total number of SNPs between the strain and representative genome, i.e. the set of strain-specific *in silico* SNPs plus inter-strain SNPs, was slightly weaker (*rho* = -0.58, $p < 10^{-15}$; Supplementary Figure 1). This overall reduction in performance with increased divergence was more strongly driven by reductions in recall (i.e., by an increased number of false negative calls) rather than precision as there was a particularly strong correlation between distance and recall (Spearman's *rho* = -0.94, $p < 10^{-15}$; Supplementary Figure 2).

Three commonly used pipelines – BWA-mem/Freebayes, BWA-mem/GATK and Novoalign/GATK – were among the highest performers when the reference genome is also the source of the reads (Table 1 and Supplementary Table 4). However, when the reference diverges from the reads, then considering the two 'overall performance' measures across the set of 10 species, Snippy instead has both the lowest sum of ranks (20) and the highest median F-score (0.982), along with the lowest number of errors per million sequenced bases (2.6) (Table 1).

Performance per species is shown in Table 2, alongside both the overall sum and range of these ranks per pipeline. Pipelines featuring Novoalign were, in general, consistently high-performing across the majority of species (that is, having a lower sum of ranks), although were outperformed by Snippy, which had both strong and uniform performance across all species (Table 2). By contrast, pipelines with a larger range of ranks had more inconsistent performance, such as minimap2/SNVer, which for example performed relatively strongly for *N. gonorrhoeae* but poorly for *S. dysenteriae* (Table 2).

While, in general, the accuracy of SNP calling declined with increasing genetic distances, some pipelines were more stable than others. If considering the median difference in F-score between SNP calls made using the same versus a representative genome, Snippy had smaller differences as the distance between genomes increased (Figure 4).

The highest ranked pipelines in Table 2 had small, but practically unimportant, differences in median F-score and so are arguably equivalently strong candidates for a 'general purpose' SNP calling solution. For instance, on the basis of F-score alone the performance of Novoalign/mpileup is negligibly different from BWA-mem/mpileup (Figure 5). However, when directly comparing pipelines, similarity of F-score distributions (see Figure 2B) can conceal larger differences in either precision or recall, categorised using the effect size estimator Cliff's delta [59, 60]. Thus, certain pipelines may be preferred if the aim is to minimise false positive (e.g. for transmission analysis) or maximise true positive (e.g. to identify antimicrobial resistance loci) calls. For instance, although Snippy (the top ranked pipeline in Table 2) is negligibly different from Novoalign/mpileup (the third ranked pipeline) in terms of F-score and precision, the former is more sensitive (Figure 5).

Comparable accuracy of SNP calling pipelines if using real rather than simulated sequencing data

We used real sequencing data from a previous study comprising 16 environmentally-sourced Gram-negative isolates (all *Enterobacteriaceae*), derived from livestock farms, sewage, and rivers, and cultures of two reference strains (*K. pneumoniae* subsp. *pneumoniae* MGH 78578 and *E. coli* CFT073), for which closed hybrid *de novo* assemblies were generated using both Illumina paired-end short reads and Nanopore long reads [61]. Source locations for each sample, species predictions and NCBI accession numbers are detailed in Supplementary Table 8. The performance statistics for each pipeline are shown in Supplementary Table 9, with an associated ranked summary in Supplementary Table 10.

Lower performance was anticipated for all pipelines, particularly for *Citrobacter* and *Enterobacter* isolates, which had comparatively high Mash distances (> 0.08) between the reads and the representative genome (Supplementary Table 8), far greater than those in the simulations (241 of the 254 simulated genomes had a Mash distance to the representative genome of < 0.04; Supplementary Table 2). Consistent with the simulations (Figure 3A), there was a strong negative correlation between Mash distance and the median F-score across

all pipelines (Spearman's *rho* = -0.83, p = 3.36×10^{-5}; Figure 6A), after excluding one prominent outlier (*E. coli* isolate RHB11-C04; see Supplementary Table 8).

Notably, the median precision of each pipeline, if calculated across the divergent set of simulated genomes, strongly correlated with the median precision calculated across the set of real genomes (Spearman's *rho* = 0.83, p = 2.81×10^{-11}; Figure 6B). While a weaker correlation was seen between simulated and real datasets on the basis of recall (Spearman's *rho* = 0.41, p = 0.007), this is consistent with the high diversity of *Enterobacteriaceae*, and the accordingly greater number of false negative calls with increased divergence (Supplementary Figure 2).

Overall, this suggests that the accuracy of a given pipeline on simulated data is a reasonable proxy for its performance on real data. While the poorer performing pipelines when using simulated data are similarly poorer performing when using real data, the top ranked pipelines differ, predominantly featuring BWA-mem, rather than Novoalign, as an aligner (Supplementary Table 10). In both cases, however, among the consistently highest performing pipelines is Snippy.

Quantitatively similar results were found when quintupling the scope of this evaluation to include 209 pipelines (Figure 7). With this Gram-negative dataset, the most consistently highly performing pipelines had little variation in F-score, irrespective of the 10-fold difference in Mash distances between reads and representative genome (Supplementary Table 8). Particularly highly performing pipelines in the expanded dataset employed the aligners NextGenMap or SMALT, and/or the variant callers LoFreq, mpileup or Strelka (Figure 7).

Discussion

Reference genome selection strongly affects SNP calling performance

Here we initially evaluated 41 SNP calling pipelines, the combination of 4 aligners with 10 callers, plus one 'all-in-one' tool, Snippy, using reads simulated from 10 clinically relevant species. These reads were first aligned back to their source genome and SNPs called. As expected under these conditions, the majority of SNP calling pipelines showed high precision and sensitivity, although between-species variation was prominent.

We next expanded the scope of the evaluation to 209 pipelines (representing the addition of 12 aligners, 4 callers, and 2 'all-in-one' pipelines, SpeedSeq and SPANDx) and introduced a degree of divergence between the reference genome and the reads, analogous to having an accurate species-level classification of the reads but no specific knowledge of the strain. For the purposes of this study, we assumed that reference genome selection was essentially arbitrary, equivalent to a community standard representative genome. Such a genome can differ significantly from the sequenced strain, which complicates SNP calling by introducing inter-specific variation between the sequenced reads and the reference. Importantly, all pipelines in this study are expected to perform well if evaluated with human data, i.e. when there is a negligible Mash distance between the reads and the reference. For example, the mean Mash distance between human assembly GRCh38.p12 and the 3 Ashkenazi assemblies of the Genome In A Bottle dataset (deep sequencing of a mother, father and son trio [62-64], available under ENA study accession PRJNA200694 and GenBank assembly accessions GCA_001549595.1, GCA_001549605.1, and GCA_001542345.1, respectively) is 0.001 (i.e., consistent with previous findings that the majority of the human genome has approximately 0.1% sequence divergence [65]). Notably, the highest performing pipeline when reads were aligned to the same genome from which they were simulated, Novoalign/GATK, was also that used by the Genome In A Bottle consortium to align human reads to the reference [62].

While tools initially benchmarked on human data, such as SNVSniffer [47], can in principle also be used on bacterial data, this study shows that in practice many perform poorly. For example, the representative *C. difficile* strain, 630, has a mosaic genome, approximately 11% of which comprises mobile genetic elements [53]. With the exception of reads simulated from *C. difficile* genomes which are erythromycin-sensitive derivatives of 630 (strains 630Derm and 630deltaerm; see [66]), aligning reads to 630 compromises accurate SNP calling, resulting in a lower median F-score across all pipelines (Figure 3). We also observed similar decreases in F-score for more recombinogenic species such as *N. gonorrhoeae*, which has a phase-variable gene repertoire [67] and has been used to illustrate the 'fuzzy species' concept, that recombinogenic bacteria do not form clear and distinct isolate clusters as assayed by phylogenies of common housekeeping loci [68, 69]. By contrast, for clonal species, such as those within the *M. tuberculosis* complex [70], the choice of reference genome has negligible influence on the phylogenetic relationships inferred from SNP calls [71] and, indeed, minimal effect on F-score.

In general, more diverse species have a broader range of Mash distances on Figure 2A (particularly notable for *E. coli*), as do those forming distinct phylogroups, such as the two clusters of *L. monocytogenes*, consistent with the division of this species into multiple primary genetic lineages [72-74].

Therefore, one major finding of this study is that, irrespective of the core components within a SNP calling pipeline, the selection of reference genome has a critical effect on output, particularly for more recombinogenic species. This can to some extent be mitigated by using variant callers that are more robust to increased distances between the reads and the reference, such as Freebayes (employed by Snippy and SpeedSeq).

A sub-optimal choice of reference genome has previously been shown to result in mapping errors, leading to biases in allelic proportions [75]. Heterologous reference genomes are in general sub-optimal for read mapping, even when there is strict correspondence between orthologous regions, with short reads particularly vulnerable to false positive alignments [76]. There is also an inverse relationship between true positive SNP calls and genetic distance, with a greater number of false positives when the reads diverge from the reference genome [22].

Study limitations

The experimental design made several simplifying assumptions regarding pipeline usage. Most notably, when evaluating SNP calling when the reference genome diverges from the source of the reads, we needed to convert the coordinates of one genome to those of another, doing so by whole genome alignment. We took a similar approach to that used to evaluate Pilon, an all-in-one tool for correcting draft assemblies and variant calling [44], which made whole genome alignments of the *M. tuberculosis* F11 and H37Rv genomes and used the resulting set of inter-strain variants as a truth set for benchmarking (a method we also used when evaluating each pipeline on real data). While this approach assumes a high degree of contiguity for the whole genome alignment, there are nevertheless significant breaks in synteny between F11 and H37Rv, with two regions deemed particularly hypervariable, in which no variant could be confidently called [44]. For the strain-to-representative genome alignments in this study, we considered SNP calls only within one-to-one alignment blocks and cannot exclude the possibility that repetitive or highly mutable regions within these blocks have been misaligned. However, we did not seek to identify and exclude SNPs from

these regions as, even if present, this would have a systematic negative effect on the performance of each pipeline. To demonstrate this, we re-calculated each performance metric for the 209 pipelines evaluated using real sequencing data after identifying, and masking, repetitive regions of the reference genome with self-self BLASTn (as in [77]). As we already required reference bases within each one-to-one alignment block to be supported by both nucmer and Parsnp calls (that is, implicitly masking ambiguous bases), we found that repeat-masking the reference genome had negligible effect on overall F-score although marginally improved precision (see Supplementary Text 1). However, it is important to note that the parameters used for repeat-masking will determine which paralogues will be successfully masked. For the purpose of this study, we used reasonably conservative parameters (detailed in Supplementary Text 1) and so expect to have primarily masked more similar paralogues. The likelihood of mis-mapping (and thereby false positive SNP calling) would increase among more divergent paralogues, although optimising parameters to detect these is non-trivial. More lenient repeat-masking parameters, in masking more divergent positions, would also reduce the number of true SNPs it is possible to call.

Furthermore, when aligning reads from one genome to a different genome, it is not possible to recover all possible SNPs introduced with respect to the former, as some will be found only within genes unique to the original genome (of which there can be many, as bacterial species have considerable genomic diversity; see Supplementary Table 5). Nevertheless, there is a strong relationship between the total number of SNPs introduced *in silico* into one genome and the maximum number of SNPs it is possible to call should reads instead be aligned to a divergent genome (Supplementary Figure 3). In any case, this does not affect the evaluation metrics used for pipeline evaluation, such as F-score, as these are based on proportional relationships of true positive, false positive and false negative calls at variant sites. However, we did not count true negative calls (and thereby assess pipeline specificity) as these can only be made at reference sites, a far greater number of which do not exist when aligning between divergent genomes.

While the programs chosen for this study are in common use and the findings generalisable, it is also important to note that they are a subset of the tools available (see Supplementary Text 1). It is also increasingly common to construct more complex pipelines that call SNPs with one tool and structural variants with another (for example, in [78]). Here, our evaluation concerned only accurate SNP calling, irrespective of the presence of structural variants

introduced by sub-optimal reference genome selection (that is, by aligning the reads to a divergent genome) and so does not test dedicated indel calling algorithms. Previous indel-specific variant calling evaluations, using human data, have recommended Platypus [8] or, for calling large indels at low read depths, Pindel [79].

Many of the findings in this evaluation are also based on simulated error-free data for which there was no clear need for pre-processing quality control. While adaptor removal and quality-trimming reads are recommended precautionary steps prior to analysing non-simulated data, previous studies differ as to whether pre-processing increases the accuracy of SNP calls [80], has minimal effect upon them [81], or whether benefits instead depend upon the aligner and reference genome used [22]. While more realistic datasets would be subject to sequencing error, we also expect this to be minimal: Illumina platforms have a per-base error rate < 0.01% [82]. Accordingly, when comparing pipelines taking either error-free or error-containing reads as input, sequencing error had negligible effect on performance (see Supplementary Text 1).

We have also assumed that given the small genome sizes of bacteria, a consistently high depth of coverage is expected in non-simulated datasets, and so have not evaluated pipeline performance on this basis (discussed further in Supplementary Text 1). In any case, a previous study found that with simulated NextSeq reads, variant calling sensitivity was largely unaffected by increases in coverage [55]. It has also been reported that random polymerase errors have minimal effect on variant calls for sequencing depths greater than 20-fold, and that these are primarily of concern only when calling minor variants [75].

Finally, so as to approximate ‘out of the box’ use conditions, we made a minimal effort application of each program with no attempt at species-specific optimisation. Had we optimised the individual components of an analytic pipeline (which, although often structured around, are not limited to one aligner and one caller), we could conceivably reduce the high variance in F-score when SNP calling from real data which, in this study, was notably divergent (see Figure 7). For instance, DeepVariant [40], a TensorFlow machine-learning based variant caller, had highly variable performance on real data but required as input a training model made using a deep neural network. At the time of use, there was currently no production-grade DeepVariant training pipeline (the default training model supplied with DeepVariant, and used in this study, was based on human data), nor were there a large

enough number of non-simulated, bacterial truth sets on which to train it. As such, we expect the performance of DeepVariant to have been under-estimated in this evaluation. Most notably, NextGenMap/DeepVariant was the most precise of the 209 pipelines evaluated on (divergent) real data (mean precision = 0.9715), although this pipeline had comparatively low recall and an accordingly poor F-score (Supplementary Table 10).

In this study we sought to use all aligners and callers uniformly, with equivalent quality-control steps applied to all reads. To that end, while direct comparisons of any aligner/caller pipeline with 'all-in-one' tools (such as Snippy, SPANDx and SpeedSeq) are possible, the results should be interpreted with caution. This is because it is in principle possible to improve the performance of the former through additional quality control steps – that is, compared to an 'all-in-one' tool, it is not necessarily the aligner or caller alone to which any difference in performance may be attributed. For instance, although Snippy and SpeedSeq employ BWA-mem and Freebayes, both tools are distinct from the BWA-mem/Freebayes pipeline used in this study (Figure 7 and Supplementary Table 10). This is because they implement additional steps between the BWA and Freebayes components, as well as altering the default parameters relative to standalone use. Snippy, for example, employs samclip (https://github.com/tseemann/samclip) to post-process the BAM file produced by BWA-mem, removing clipped alignments in order to reduce false positive SNPs near structural variants.

Recommendations for bacterial SNP calling

Our results emphasise that one of the principal difficulties of alignment-based bacterial SNP calling is not pipeline selection *per se* but optimal reference genome selection (or, alternatively, its *de novo* creation, not discussed further). If assuming all input reads are from a single, unknown, origin, then in principle a reference genome could be predicted using a metagenomic classifier such as Centrifuge [83], CLARK [84], Kaiju [85] or Kraken [86]. However, correctly identifying the source genome from even a set of single-origin reads is not necessarily simple with the performance of read classifiers depending in large part on the sequence database they query (such as, for instance, EMBL proGenomes [87] or NCBI RefSeq [88]), which can vary widely in scope, redundancy, and degree of curation (see performance evaluations [89, 90]). This is particularly evident among the *Citrobacter* samples in the real dataset, with 3 methods each making different predictions (Supplementary Table 8). Specialist classification tools such as Mykrobe [91] use customised, tightly curated,

allele databases and perform highly for certain species (in this case, *M. tuberculosis* and *S. aureus*) although by definition do not have wider utility. An additional complication would also arise from taxonomic disputes such as, for example, *Shigella* spp. being essentially indistinct from *E. coli* [92].

One recommendation, which is quick and simple to apply, would be to test which of a set of candidate reference genomes is most suitable by estimating the distance between each genome and the reads. This can be accomplished using Mash [58], which creates 'sketches' of sequence sets (compressed representations of their k-mer distributions) and then estimates the Jaccard index (that is, the fraction of shared k-mers) between each pair of sequences. Mash distances are a proxy both for average nucleotide identity [58] and measures of genetic distance derived from the whole genome alignment of genome pairs (Supplementary Table 2), correlating strongly with the total number of SNPs between the strain genome and the representative genome (Spearman's *rho* = 0.97, $p < 10^{-15}$), and to a reasonable degree with the proportion of bases unique to the strain genome (Spearman's *rho* = 0.48, $p < 10^{-15}$). More closely related genomes would have lower Mash distances and so be more suitable as reference genomes for SNP calling. This would be particularly appropriate if, for example, studying transmission events as a closely-related reference would increase specificity, irrespective of the aligner or caller used. For larger studies that require multiple samples to be processed using a common reference, the choice of reference genome could be one which 'triangulates' between the set of samples – that is, has on average a similar distance to each sample, rather than being closer to some and more distant from others.

Using a highly divergent genome (such as the representative *Enterobacter* genomes in the real dataset, each of which differs from the reads by a Mash distance > 0.1; Supplementary Table 8) is analogous to variant calling in a highly polymorphic region, such as the human leukocyte antigen, which shows > 10% sequence divergence between haplotypes [65] (i.e., even for pipelines optimised for human data – the majority in this study – this would represent an anomalous use case).

Prior to using Mash (or other sketch-based distance-estimators, such as Dashing [93] or FastANI [94]), broad-spectrum classification tools such as Kraken could be used to narrow down the scope of the search space to a set of fully-sequenced candidate genomes, i.e. those genomes of the taxonomic rank to which the highest proportion of reads could be assigned

with confidence. This approach is similar to that implemented by the Python package PlentyOfBugs (https://github.com/nickp60/plentyofbugs) which, assuming you already know the species or genus, automates the process of downloading and sketching candidate genomes to create a database for querying with Mash.

In the future, reads from long-read sequencing platforms, such as Oxford Nanopore and PacBio, are less likely to be ambiguously mapped within a genomic database and so in principle are simpler to classify (sequencing error rate notwithstanding), making it easier to select a suitable reference genome. However, long-read platforms can also, in principle if not yet routinely, generate complete *de novo* bacterial genomes [95] for downstream SNP calling, possibly removing the need to choose a reference entirely. Similarly, using a reference pan-genome instead of a singular representative genome could also maximise the number of SNP calls by reducing the number of genes not present in the reference [96]. A popular means of representing the pan-genome, as used by tools such as Roary [97], is as a collection of individual consensus sequences, ostensibly genes but more specifically open reading frames with protein-coding potential. This use of consensus sequences could also reduce the number of nucleotide differences between a set of sequenced reads (which may be from a highly divergent strain) and the (consensus) reference.

An alternative approach to reducing errors introduced when using a single reference genome could be to merge results from multiple reference genomes (the approach taken by REALPHY to reconstruct phylogenies from bacterial SNPs [98]) or from multiple aligners and/or callers, obtaining consensus calls across a set of methods. This is the approach taken by the NASP pipeline [99], which can integrate data from any combination of the aligners Bowtie2, BWA-mem, Novoalign and SNAP, and the callers GATK, mpileup, SolSNP and VarScan (ensemble approaches have similarly been used for somatic variant calling, for example by SomaticSeq [100]).

If considering the overall performance of a pipeline as the sum of the 7 different ranks for the different metrics considered, then averaged across the full set of species' genomes, the highest performing pipelines are, with simulated data, Snippy and those utilising Novoalign in conjunction with LoFreq or mpileup (Table 2), and with real (more divergent) data, those utilising NextGenMap or SMALT in conjunction with LoFreq, mpileup or Strelka (Supplementary Table 10).

Some of the higher-performing tools apply error-correction models that also appear suited to bacterial datasets with high SNP density, despite their original primary use case being in different circumstances. For instance, SNVer (which in conjunction with BWA-mem, ranks second to Snippy for *N. gonorrhoeae*; see Table 2) implements a statistical model for calling SNPs from pooled DNA samples, where variant allele frequencies are not expected to be either 0, 0.5 or 1 [46]. SNP calling from heterogeneous bacterial populations with high mutation rates, in which only a proportion of cells may contain a given mutation, is also conceptually similar to somatic variant calling in human tumours, where considerable noise is expected [75]. This is a recommended use case for Strelka, which performed highly on real (and particularly divergent) data, being among the top-performing pipelines when paired with many aligners (Figure 7).

Irrespective of pipeline employed, increasing Mash distances between the reads and the reference increases the number of false negative calls (Supplementary Figure 2). Nevertheless, Snippy, which employs Freebayes, is particularly robust to this, being among the most sensitive pipelines when evaluated using simulated data (Figure 5 and Supplementary Figure 4). Notably, Freebayes is haplotype-based, calling variants based on the literal sequence of reads aligned to a particular location, so avoiding the problem of one read having multiple possible alignments (increasingly likely with increasing genomic diversity) but only being assigned to one of them. However, as distance increases further, it is likely that reads will cease being misaligned (which would otherwise increase the number of false positive calls) but rather they will not be aligned at all, being too dissimilar to the reference genome.

With an appropriate selection of reference genome, many of these higher-performing pipelines could be optimised to converge on similar results by tuning parameters and post-processing VCFs with specific filtering criteria, another routine task for which there are many different choices of application [101-104]. In this respect, the results of this study should be interpreted as a range-finding exercise, drawing attention to those SNP calling pipelines which, under default conditions, are generally higher-performing and which may be most straightforwardly optimised to meet user requirements.

Conclusions

We have performed a comparison of SNP calling pipelines across both simulated and real data in multiple bacterial species, allowing us to benchmark their performance for this specific use. We find that all pipelines show extensive species-specific variation in performance, which has not been apparent from the majority of existing, human-centred, benchmarking studies. While aligning to a single representative genome is common practice in eukaryotic SNP calling, in bacteria the sequence of this genome may diverge considerably from the sequence of the reads. A critical factor affecting the accuracy of SNP calling is thus the selection of a reference genome for alignment. This is complicated by ambiguity as to the strain of origin for a given set of reads, which is perhaps inevitable for many recombinogenic species, a consequence of the absence (or impossibility) of a universal species concept for bacteria (but see [105]). For many clinically common species, excepting *M. tuberculosis*, the use of standard 'representative' reference genomes can compromise accurate SNP calling by disregarding genomic diversity. By first considering the Mash distance between the reads and a candidate set of reference genomes, a genome with minimal distance may be chosen that, in conjunction with one of the higher performing pipelines, can maximise the number of true variants called.

Materials and Methods

Simulating truth sets of SNPs for pipeline evaluation

264 genomes, representing a range of strains from 10 bacterial species, and their associated annotations, were obtained from the NCBI Genome database [106] (https://www.ncbi.nlm.nih.gov/genome, accessed 16th August 2018), as detailed in Supplementary Table 2. One genome per species is considered to be a representative genome (criteria detailed at https://www.ncbi.nlm.nih.gov/refseq/about/prokaryotes/, accessed 16th August 2018), indicated in Supplementary Table 2. Strains with incomplete genomes (that is, assembled only to the contig or scaffold level) or incomplete annotations (that is, with no associated GFF, necessary to obtain gene coordinates) were excluded, as were those with multiple available genomes (that is, the strain name was not unique). After applying these filters, all species were represented by approx. 30 complete genomes (28 *C. difficile*, 29 *M. tuberculosis* and 36 *S. pneumoniae*), with the exceptions of *N. gonorrhoeae* (n = 15) and *S. dysenteriae* (n = 2). For the 5 remaining species (*E. coli*, *K. pneumoniae*, *L. monocytogenes*, *S. aureus* and *S. enterica*), there are > 100 usable genomes each. As it was not

computationally tractable to test every genome, we chose a subset of isolates based on stratified selection by population structure. We created all-against-all distance matrices using the 'triangle' component of Mash v2.1 [58], then constructed dendrograms (Supplementary Figures 5 to 9) from each matrix using the neighbour joining method, as implemented in MEGA v7.0.14 (MEGA Software , RRID:SCR_000667)[107]. By manually reviewing the topology, 30 isolates were chosen per species to create a representative sample of its diversity.

For each genome used in this study, we excluded, if present, any non-chromosomal (i.e. circular plasmid) sequence. A simulated version of each core genome, with exactly 5 randomly generated SNPs per genic region, was created using Simulome v1.2 [108] with parameters --whole_genome=TRUE --snp=TRUE --num_snp=5. As the coordinates of some genes overlap, not all genes will contain simulated SNPs. The number of SNPs introduced into each genome (from approximately 8000 to 25,000) and the median distance between SNPs (from approximately 60 to 120 bases) is detailed in Supplementary Table 2.

The coordinates of each SNP inserted into a given genome are, by definition, genome- (that is, strain-) specific. As such, it is straightforward to evaluate pipeline performance when reads from one genome are aligned to the same reference. However, in order to evaluate pipeline performance when reads from one genome are aligned to the genome of a divergent strain (that is, the representative genome of that species), the coordinates of each strain's genome need to be converted to representative genome coordinates. To do so, we made whole genome (core) alignments of the representative genome to both versions of the strain genome (one with and one without SNPs introduced *in silico*) using nucmer and dnadiff, components of MUMmer v4.0.0beta2 [56], with default parameters (illustrated in Figure 1). For one-to-one alignment blocks, differences between each pair of genomes were identified using MUMmer show-snps with parameters -Clr -x 1, with the tabular output of this program converted to VCF by the script MUMmerSNPs2VCF.py (https://github.com/liangjiaoxue/PythonNGSTools, accessed 16th August 2018). The two resulting VCFs contain the location of all SNPs relative to the representative genome (i.e. inclusive of those introduced *in silico*), and all inter-strain variants, respectively. We excluded from further analysis two strains with poor-quality strain-to-representative whole genome alignments, both calling < 10% of the strain-specific *in silico* SNPs (Supplementary Table 11). The proportion of *in silico* SNPs recovered by whole genome alignment is detailed

in Supplementary Table 11 and is, in general, high: of the 254 whole genome alignments of non-representative to representative strains across the 10 species, 222 detect > 80% of the *in silico* SNPs and 83 detect > 90%. For the purposes of evaluating SNP calling pipelines when the reference genome differs from the reads, we are concerned only with calling the truth set of *in silico* SNPs and so discard inter-strain variants (see below). More formally, when using each pipeline to align reads to a divergent genome, we are assessing the concordance of its set of SNP calls with the set of nucmer calls. However, it is possible that for a given call, one or more of the pipelines are correct and nucmer is incorrect. To reduce this possibility, a parallel set of whole genome alignments were made using Parsnp v1.2 with default parameters [57], with the exported SNPs contrasted with the nucmer VCF.

Thus, when aligning to a divergent genome, the truth set of *in silico* SNPs (for which each pipeline is scored for true positives) are those calls independently identified by both nucmer and Parsnp. Similarly, the set of inter-strain positions are those calls made by one or both of nucmer and Parsnp. As we are not concerned with the correctness of these calls, the lack of agreement between the two tools is not considered further; rather, this establishes a set of ambiguous positions which are discarded when VCFs are parsed.

Simulated SNP-containing genomes, sets of strain-to-representative genome SNP calls (made by both nucmer and Parsnp), and the final truth sets of SNPs are available in Supplementary Dataset 1 (hosted online via the Oxford Research Archive at http://dx.doi.org/10.5287/bodleian:AmNXrjYN8).

Evaluating SNP calling pipelines using simulated data

From each of 254 SNP-containing genomes, 3 sets of 150bp and 3 sets of 300bp paired-end were simulated using wgsim, a component of SAMtools v1.7 (SAMTOOLS , RRID:SCR_002105)[21]. This requires an estimate of average insert size (the length of DNA between the adapter sequences), which in real data is often variable, being sensitive to the concentration of DNA used [109]. For read length *x*, we assumed an insert size of 2.2*x*, i.e. for 300bp reads, the insert size is 660bp (Illumina paired-end reads typically have an insert longer than the combined length of both reads [110]). The number of reads simulated from each genome is detailed in Supplementary Table 3 and is equivalent to a mean 50-fold base-level coverage, i.e. (50 x genome length)/read length.

Perfect (error-free) reads were simulated from each SNP-containing genome using wgsim parameters -e 0 -r 0 -R 0 -X 0 -A 0 (respectively, the sequencing error rate, mutation rate, fraction of indels, probability an indel is extended, and the fraction of ambiguous bases allowed).

Each set of reads was then aligned both to the genome of the same strain and to the representative genome of that species (from which the strain will diverge), with SNPs called using 41 different SNP calling pipelines (10 callers each paired with 4 aligners, plus the self-contained Snippy). The programs used, including version numbers and sources, are detailed in Supplementary Table 1, with associated command lines in Supplementary Text 1. All pipelines were run using a high-performance cluster employing the Open Grid Scheduler batch system on Scientific Linux 7. No formal assessment was made of pipeline run time or memory usage. This was because given the number of simulations it was not tractable to benchmark run time using, for instance, a single core. The majority of programs in this study permit multithreading (all except the callers 16GT, GATK, Platypus, SNVer, and SNVSniffer) and so are in principle capable of running very rapidly. We did not seek to optimise each tool for any given species and so made only a minimum effort application of each pipeline, using default parameters and minimal VCF filtering (see below). This is so that we obtain the maximum possible number of true positives from each pipeline under reasonable use conditions.

While each pipeline comprises one aligner and one caller, there are several ancillary steps common in all cases. After aligning reads to each reference genome, all BAM files were cleaned, sorted, had duplicate reads marked and were indexed using Picard Tools v2.17.11 (Picard, RRID:SCR_006525)[111] CleanSam, SortSam, MarkDuplicates and BuildBamIndex, respectively. We did not add a post-processing step of local indel realignment (common in older evaluations, e.g., [12]) as this had negligible effect upon pipeline performance, with many variant callers (including GATK HaplotypeCaller [25] (GATK , RRID:SCR_001876) and Freebayes(FreeBayes, RRID:SCR_010761)) already incorporating a method of haplotype assembly (see Supplementary Text 1).

Each pipeline produces a VCF as its final output. As with a previous evaluation [26], all VCFs were regularised using the vcfallelicprimitives module of vcflib v1.0.0-rc2 (https://github.com/ekg/vcflib), so that different representations of the same indel or complex

variant were not counted separately (these variants can otherwise be presented correctly in multiple ways). This module splits adjacent SNPs into individual SNPs, left-aligns indels and regularizes the representation of complex variants. The set of non-regularised VCFs cannot be meaningfully compared (see Supplementary Text 1).

Different variant callers populate their output VCFs with different contextual information. Before evaluating the performance of each pipeline, all regularised VCFs were subject to minimal parsing to retain only high-confidence variants. This is because many tools record variant sites even if they have a low probability of variation, under the reasonable expectation of parsing. Some tools (including Snippy and SNVer) apply their own internal set of VCF filtering criteria, giving the user the option of a 'raw' or 'filtered' VCF; in such cases, we retain the filtered VCF as the default recommendation. Where possible, (additional) filter criteria were applied as previously used by, and empirically selected for, COMPASS (Complete Pathogen Sequencing Solution; https://github.com/oxfordmmm/CompassCompact), an analytic pipeline employing Stampy and mpileup for base calling non-repetitive core genome sites (outlined in Supplementary Text 1 with filter criteria described in [112] and broadly similar to those recommended by a previous study for maximising SNP validation rate [113]). No set of generic VCF hard filters can be uniformly applied because each caller quantifies different metrics (such as the number of forward and reverse reads supporting a given call) and/or reports the outcome of a different set of statistical tests, making filtering suggestions on this basis. For instance, in particular circumstances, GATK suggests filtering on the basis of the fields 'FS', 'MQRankSum' and 'ReadPosRankSum', which are unique to it (detailed at https://software.broadinstitute.org/gatk/documentation/article.php?id=6925, accessed 2nd April 2019). Where the relevant information was included in the VCF, SNPs were required to have (a) a minimum Phred score of 20, (b) $\geq$ 5 reads mapped at that position, (c) at least one read in each direction in support of the variant, and (d) $\geq$ 75% of reads supporting the alternative allele. These criteria were implemented with the 'filter' module of BCFtools v1.7 [21] using parameters detailed in Supplementary Table 12.

From these filtered VCFs, evaluation metrics were calculated as detailed below.

Evaluating SNP calling pipelines using real sequencing data

Parallel sets of 150 bp Illumina HiSeq 4000 paired-end short reads and ONT long reads were obtained from 16 environmentally-sourced samples from the REHAB project (‘the environmental REsistome: confluence of Human and Animal Biota in antibiotic resistance spread’; http://modmedmicro.nsms.ox.ac.uk/rehab/), as detailed in [61]: 4 *Enterobacter* spp., 4 *Klebsiella* spp., 4 *Citrobacter* spp., and 4 *Escherichia coli*, with species identified using MALDI-TOF (matrix-assisted laser desorption ionization time-of-flight) mass spectrometry, plus sub-cultures of stocks of two reference strains *K. pneumoniae* subsp. *pneumoniae* MGH 78578 and *E. coli* CFT073. Additional predictions were made using both the protein- and nucleotide-level classification tools Kaiju v1.6.1 [85] and Kraken2 v2.0.7 (Kraken, RRID:SCR_005484)[114], respectively. Kaiju was used with two databases, one broad and one deep, both created on 5th February 2019: ‘P’ (http://kaiju.binf.ku.dk/database/kaiju_db_progenomes_2019-02-05.tgz; > 20 million bacterial and archaeal genomes from the compact, manually curated, EMBL proGenomes [115], supplemented by approximately 10,000 viral genomes from NCBI RefSeq [116]) and ‘E’ (http://kaiju.binf.ku.dk/database/kaiju_db_nr_euk_2019-02-05.tgz; > 100 million bacterial, archaeal, viral and fungal genomes from NCBI nr, alongside various microbial eukaryotic taxa). Kaiju was run with parameters -e 5 and -E 0.05 which, respectively, allow 5 mismatches per read and filter results on the basis of an E-value threshold of 0.05. The read classifications from both databases were integrated using the Kaiju ‘mergeOutputs’ module, which adjudicates based on the lowest taxonomic rank of each pair of classifications, provided they are within the same lineage, else re-classifies the read at the lowest common taxonomic rank ancestral to the two. Kraken2 was run with default parameters using the MiniKraken2 v1 database (https://ccb.jhu.edu/software/kraken2/dl/minikraken2_v1_8GB.tgz, created 12th October 2018), which was built from the complete set of NCBI RefSeq bacterial, archaeal and viral genomes.

Hybrid assemblies were produced using methods detailed in [61] and briefly recapitulated here. Illumina reads were processed using COMPASS (see above). ONT reads were adapter-trimmed using Porechop v0.2.2 (https://github.com/rrwick/Porechop) with default parameters, and then error-corrected and sub-sampled (preferentially selecting the longest reads) to 30-40x coverage using Canu v1.5 (Canu, RRID:SCR_015880)[117] with default parameters. Finally, Illumina-ONT hybrid assemblies for each genome were generated using Unicycler v0.4.0 [54] with default parameters. The original study found high agreement

between these assemblies and those produced using hybrid assembly with PacBio long reads rather than ONT, giving us high confidence in their robustness.

In the simulated datasets, SNPs are introduced *in silico* into a genome, with reads containing these SNPs then simulated from it. With this dataset, however, there are no SNPs within each genome: we have only the short reads (that is, real output from an Illumina sequencer) and the genome assembled from them (with which there is an expectation of near-perfect read mapping).

To evaluate pipeline performance when the reads are aligned to a divergent genome, reference genomes were selected as representative of the predicted species, with distances between the two calculated using Mash v2.1 [58] and spanning approximately equal intervals from 0.01 to 0.12 (representative genomes and Mash distances are detailed in Supplementary Table 8). The truth set of SNPs between the representative genome and each hybrid assembly was the intersection of nucmer and Parsnp calls, as above.

Samples, source locations, MALDI ID scores and associated species predictions are detailed in Supplementary Table 8. Raw sequencing data have been deposited with the NCBI under BioProject accession PRJNA422511 (https://www.ncbi.nlm.nih.gov/bioproject/PRJNA422511), with the associated hybrid assemblies available via FigShare[118].

To allow both the replication and expansion of this evaluation using real sequencing data, a complete archive is available as Supplementary Dataset 2 (hosted online via the Oxford Research Archive at https://ora.ox.ac.uk/objects/uuid:8f902497-955e-4b84-9b85-693ee0e4433e) comprising reads, assemblies, indexed reference genomes, the associated SNP call truth sets, VCFs, and a suite of Perl scripts.

Evaluation metrics

For each pipeline, we calculated the absolute number of true positive (TP; the variant is in the simulated genome and correctly called by the pipeline), false positive (FP; the pipeline calls a variant which is not in the simulated genome) and false negative SNP calls (FN; the variant is in the simulated genome but the pipeline does not call it). We did not calculate true negative calls for two reasons. Firstly, to do so requires a VCF containing calls for all sites, a function

offered by some variant callers (such as mpileup) but not all. Secondly, when aligning reads to a divergent genome, a disproportionately large number of reference sites will be excluded, particularly in more diverse species (for example, gene numbers in *N. gonorrhoeae* differ by up to a third; see Supplementary Table 5).

We then calculated the precision (positive predictive value) of each pipeline as TP/(TP+FP), recall (sensitivity) as TP/(TP+FN), miss rate as FN/(TP+FN), and total number of errors (FP+FN) per million sequenced bases. We did not calculate specificity as this depends on true negative calls. We also calculated the F-score (as in [55]), which considers precision and recall with equal weight: F = 2 * ((precision * recall) / (precision + recall)). The F-score evaluates each pipeline as a single value bounded between 0 and 1 (perfect precision and recall). We also ranked each pipeline based on each metric so that – for example – the pipeline with the highest F-score, and the pipeline with the lowest number of false positives, would be rank 1 in their respective distributions. As an additional ‘overall performance’ measure, we calculated the sum of ranks for the 7 core evaluation metrics (the absolute numbers of TP, FP and FN calls, and the proportion-based precision, recall, F-score, and total error rate per million sequenced bases). Pipelines with a lower sum of ranks would, in general, have higher overall performance.

We note that when SNPs are called after aligning reads from one strain to that of a divergent strain, the SNP calling pipeline will call positions for both the truth set of strain-specific *in silico* SNPs and any inter-strain variants. To allow a comparable evaluation of pipelines in this circumstance, inter-strain calls (obtained using nucmer and Parsnp; see above) are discarded and not explicitly considered either true positive, false positive or false negative. While the set of true SNPs when aligning to a divergent strain will be smaller than that when aligned to the same strain (because all SNPs are simulated in genic regions but not all genes are shared between strains), this will not affect proportion-based evaluation metrics, such as F-score.

Effect size of differences in the F-score distribution between pipelines

Differences between distributions are assessed by Mann Whitney U tests, with results interpreted using the non-parametric effect size estimator Cliff’s delta [59, 60], estimated at a confidence level of 95% using the R package effsize v0.7.1 [119]. Cliff’s delta employs the concept of dominance (which refers to the degree of overlap between distributions) and so is

more robust when distributions are skewed. Estimates of delta are bound in the interval (-1,1), with extreme values indicating a lack of overlap between groups (respectively, set 1 << set 2 and set 1 >> set 2). Distributions with |delta| < 0.147 are negligibly different, as in [120]. Conversely, distributions with |delta| >= 0.60 are considered to have large differences.

Tables

Table 1. Summary of pipeline performance across all species' genomes.

Table 2. Overall performance of each pipeline per species, calculated as the sum of seven ranks, when reads are aligned to a divergent genome.
The seven performance measures for each pipeline (the absolute numbers of true positive, false positive and false negative calls, and the proportion-based precision, recall, F-score, and total error rate per million sequenced bases) are detailed in Supplementary Table 6, with associated ranks in Supplementary Table 7.

Figures

Figure 1. Overview of SNP calling evaluation.
SNPs were introduced *in silico* into 254 closed bacterial genomes (Supplementary Table 2) using Simulome. Reads were then simulated from these genomes. 41 SNP calling pipelines (Supplementary Table 1) were evaluated using two different genomes for read alignment: the original genome from which the reads were simulated and a divergent genome, the species-representative NCBI 'reference genome'. In the latter case, it will not be possible to recover all of the original *in silico* SNPs as some will be found only within genes unique to the original genome. Accordingly, to evaluate SNP calls, the coordinates of the original genome need to be converted to those of the representative genome. To do so, whole genome alignments were made using both nucmer and Parsnp, with consensus calls identified within one-to-one alignment blocks. Inter-strain SNPs (those not introduced *in silico*) are excluded. The remaining subset of *in silico* calls comprise the truth set for evaluation. There is a strong correlation between the total number of SNPs introduced *in silico* into the original genome and the total number of nucmer/Parsnp consensus SNPs in the divergent genome (Supplementary Figure 3).

Figure 2. Median F-score per pipeline when the reference genome for alignment is (A) the same as the source of the reads, and (B) a representative genome for that species. Panels show the median F-score of 41 different pipelines when SNPs are called using error-free 150bp and 300bp reads simulated from 254 genomes (of 10 species) at 50-fold coverage. Pipelines are ordered according to median F-score and coloured according to either the variant caller (A) or aligner (B) in each pipeline. Note that because F-scores are uniformly > 0.9 when the reference genome for alignment is the same as the source of the reads, the vertical axes on each panel have different scales. Genomes are detailed in Supplementary Table 2, summary statistics for each pipeline in Supplementary Tables 3 and 6, and performance ranks in Supplementary Tables 4 and 7, for alignments to the same or to a representative genome, respectively.

Figure 3. Reduced performance of SNP calling pipelines with increasing genetic distance between the reads and the reference genome. The median F-score across the complete set of 41 pipelines, per strain, decreases as the distance between the strain and the reference genome increases (assayed as the Mash distance, which is based on the proportion of k-mers shared between genomes). Each point indicates the median F-score, across all pipelines, for the genome of one strain per species (n = 254 strains). Points are coloured by the species of each strain (n = 10 species). Summary statistics for each pipeline are shown in Supplementary Table 6, performance ranks in Supplementary Table 7 and the genetic distance between strains in Supplementary Table 2. Quantitatively similar results are seen if assaying distance as the total number of SNPs between the strain and representative genome, i.e. the set of strain-specific *in silico* SNPs plus inter-strain SNPs (Supplementary Figure 1).

Figure 4. Stability of pipeline performance, in terms of F-score, with increasing genetic distance between the reads and the reference genome. The performance of a SNP calling pipeline decreases with increasing distance between the genome from which reads are sequenced and the reference genome to which they are aligned. Each point shows the median difference in F-score for a pipeline that calls SNPs when the reference genome is the same as the source of the reads, and when it is instead a representative genome for that species. Points are coloured according to the variant caller in each pipeline, with those towards the top of the figure less affected by distance. Lines fitted using LOESS smoothing.

Figure 5. Head-to-head performance comparison of three pipelines using simulated data, on the basis of precision, recall and F-score.

This figure directly compares the performance of three pipelines using simulated data: Snippy, Novoalign/mpileup and BWA/mpileup. Each point indicates the median F-score, precision or recall (columns 1 through 3, respectively), for the genome of one strain per species (n = 254 strains). Raw data for this figure is given in Supplementary Table 6. Text in the top left of each figure is an interpretation of the difference between each pair of distributions, obtained using the R package 'effsize' which applies the non-parametric effect size estimator Cliff's delta to the results of a Mann Whitney U test. An expanded version of this figure, comparing 40 pipelines relative to Snippy, is given as Supplementary Figure 4.

Figure 6. Similarity of performance for pipelines evaluated using both simulated and real sequencing data.

Panel A shows that pipelines evaluated using real sequencing data show reduced performance with increasing Mash distances between the reads and the reference genome, similar to that observed with simulated data (see Figure 3A). Each point indicates the median F-score, across all pipelines, for the genome of an environmentally-sourced/reference isolate (detailed in Supplementary Table 8). Panel B shows that pipelines evaluated using real and simulated sequencing data have comparable accuracy. Each point shows the median precision of each of 41 pipelines, calculated across both a divergent set of 254 simulated genomes (2-36 strains from ten clinically common species) and 18 real genomes (isolates of *Citrobacter*, *Enterobacter*, *Escherichia* and *Klebsiella*). The outlier pipeline, with lowest precision on both real and simulated data, is Stampy/Freebayes. Raw data for this figure are available in Supplementary Tables 6 (simulated genomes) and 9 (real genomes).

Figure 7. Median F-score per pipeline using real sequencing data, and when the reference genome for alignment can diverge considerably from the source of the reads.

This figure shows the F-score distribution of 209 pipelines evaluated using real sequencing data sourced from the REHAB project and detailed in [61]. This dataset comprises 16 environmentally-sourced Gram-negative isolates (all *Enterobacteriaceae*), and cultures of two reference strains (*K. pneumoniae* subsp. *pneumoniae* MGH 78578 and *E. coli* CFT073). For this figure, data from one outlier, *E. coli* isolate RHB11-C04, is excluded. Raw data for

this figure is available as Supplementary Table 9, with summary statistics for each pipeline detailed in Supplementary Table 10. Genomes are detailed in Supplementary Table 8.

Supplementary Tables

Supplementary Table 1. Sources of software.

Supplementary Table 2. Genomes into which SNPs were introduced *in silico*, and various measures of distance between each strain's genome and the representative genome of that species.

Supplementary Table 3. Summary statistics of SNP calling pipelines after aligning simulated reads to the same reference genome as their origin.

Supplementary Table 4. Ranked performance of SNP calling pipelines after aligning simulated reads to the same reference genome as their origin.

Supplementary Table 5. Genome size diversity within 5 clinically common bacterial species.

Supplementary Table 6. Summary statistics of SNP calling pipelines after aligning simulated reads to a reference genome differing from their origin.

Supplementary Table 7. Ranked performance of SNP calling pipelines after aligning simulated reads to reference genome differing from their origin.

Supplementary Table 8. Environmentally-sourced/reference Gram-negative isolates and associated representative genomes.

Supplementary Table 9. Summary statistics of SNP calling pipelines after aligning real reads to a reference genome differing from their origin.

Supplementary Table 10. Ranked performance of SNP calling pipelines after aligning real reads to reference genome differing from their origin.

Supplementary Table 11. Proportion of strain-specific *in silico* SNPs detected in whole genome alignments between the strain genome and a representative genome.

Supplementary Table 12. VCF filtering parameters, as used by BCFtools.

Supplementary Table 13. Summary statistics of SNP calling pipelines after aligning both simulated error-free and error-containing reads to the same reference genome as their origin.

Supplementary Table 14. Summary statistics of SNP calling pipelines after aligning both simulated error-free and error-containing reads to a reference genome differing from their origin.

Supplementary Table 15. Summary statistics of SNP calling pipelines after aligning simulated error-free reads to a reference genome differing from their origin, both with and without local indel realignment.

Supplementary Table 16. Summary statistics of *E. coli* SNP calling pipelines after aligning simulated error-free reads to a reference genome differing from their origin, both with and without VCF regularisation.

Supplementary Table 17. Summary statistics of *E. coli* SNP calling pipelines after aligning simulated error-free reads to a reference genome differing from their origin, at 5-, 10-, 25- and 50-fold depths of coverage.

Supplementary Figures

Supplementary Figure 1. Reduced performance of SNP calling pipelines with increasing genetic distance between the reads and the reference genome (assayed as total number of SNPs).
The median F-score across a set of 41 pipelines, per strain, decreases as the distance between the strain and the reference genome increases (assayed as the total number of SNPs between the strain and representative genome, i.e. the set of strain-specific *in silico* SNPs plus inter-strain SNPs). Each point indicates the genome of one strain per species (n = 254 strains).

Points are coloured by the species of each strain (n = 10 species). Summary statistics for each pipeline are shown in Supplementary Table 6, performance ranks in Supplementary Table 7 and the genetic distance between strains in Supplementary Table 2. Quantitatively similar results are seen if assaying distance as the Mash distance, which is based on the proportion of k-mers shared between genomes (Figure 3).

Supplementary Figure 2. Decreasing sensitivity (that is, an increased number of false negative calls) with increasing genetic distance between the reads and the reference genome (assayed as Mash distance).
The median sensitivity (recall) across a set of 41 pipelines, per strain, increases as the distance between the strain and the reference genome increases (assayed as the Mash distance, which is based on the proportion of shared k-mers between genomes). Each point indicates the genome of one strain per species (n = 254 strains). Points are coloured by the species of each strain (n = 10 species). Summary statistics for each pipeline are shown in Supplementary Table 6, performance ranks in Supplementary Table 7 and the genetic distance between strains in Supplementary Table 2.

Supplementary Figure 3. Total number of SNPs it is possible to call should reads from one strain be aligned to a representative genome of that species.
Strong correlation between the total number of SNPs introduced *in silico* into one genome and the maximum number of SNPs it is possible to call assuming reads from the former are aligned to a representative genome of that species (which will not necessarily contain the same complement of genes). Each point represents the genome of one strain, with genomes detailed in Supplementary Table 2. The line y = x is shown in red.

Supplementary Figure 4. Head-to-head performance comparison of all pipelines relative to Snippy, on the basis of F-score, using simulated data.
This figure directly compares the performance, using simulated data, of 40 pipelines relative to Snippy. Each point indicates the median F-score for the genome of one strain per species (n = 254 strains). Data for Snippy is plotted on the x-axis, and for the named pipeline on the y-axis. Raw data for this figure is given in Supplementary Table 6. Text in the top left of each figure is an interpretation of the difference between each pair of distributions, obtained using the R package ‘effsize’ which applies the non-parametric effect size estimator Cliff’s delta to the results of a Mann Whitney U test.

Supplementary Figure 5. Selection of *E. coli* isolates by manual review of dendrogram topology.

There are numerous usable complete genomes for *E. coli*. For the SNP calling evaluation, a subset of isolates was selected (indicated in red boxes) so as to maximise the diversity of clades represented. To do so, an all-against-all distance matrix for each genome was created using the 'triangle' component of Mash v2.1, with a dendrogram constructed using the neighbour joining method implemented in MEGA v7.0.14. Sources for the selected genomes are given in Supplementary Table 2.

Supplementary Figure 6. Selection of *K. pneumoniae* isolates by manual review of dendrogram topology.

There are numerous usable complete genomes for *K. pneumoniae*. For the SNP calling evaluation, a subset of isolates was selected (indicated in red boxes) so as to maximise the diversity of clades represented. To do so, an all-against-all distance matrix for each genome was created using the 'triangle' component of Mash v2.1, with a dendrogram constructed using the neighbour joining method implemented in MEGA v7.0.14. Sources for the selected genomes are given in Supplementary Table 2.

Supplementary Figure 7. Selection of *L. monocytogenes* isolates by manual review of dendrogram topology.

There are numerous usable complete genomes for *L. monocytogenes*. For the SNP calling evaluation, a subset of isolates was selected (indicated in red boxes) so as to maximise the diversity of clades represented. To do so, an all-against-all distance matrix for each genome was created using the 'triangle' component of Mash v2.1, with a dendrogram constructed using the neighbour joining method implemented in MEGA v7.0.14. Sources for the selected genomes are given in Supplementary Table 2.

Supplementary Figure 8. Selection of *S. enterica* isolates by manual review of dendrogram topology.

There are numerous usable complete genomes for *S. enterica*. For the SNP calling evaluation, a subset of isolates was selected (indicated in red boxes) so as to maximise the diversity of clades represented. To do so, an all-against-all distance matrix for each genome was created using the 'triangle' component of Mash v2.1, with a dendrogram constructed using the

neighbour joining method implemented in MEGA v7.0.14. Sources for the selected genomes are given in Supplementary Table 2.

Supplementary Figure 9. Selection of *S. aureus* isolates by manual review of dendrogram topology.

There are numerous usable complete genomes for *S. aureus*. For the SNP calling evaluation, a subset of isolates was selected (indicated in red boxes) so as to maximise the diversity of clades represented. To do so, an all-against-all distance matrix for each genome was created using the ‘triangle’ component of Mash v2.1, with a dendrogram constructed using the neighbour joining method implemented in MEGA v7.0.14. Sources for the selected genomes are given in Supplementary Table 2.

Supplementary Datasets

Supplementary Dataset 1. Simulated datasets for evaluating bacterial SNP calling pipelines.

This archive contains the set of 254 SNP-containing genomes, VCFs containing the nucmer and Parsnp strain-to-representative genome SNP calls, and the final truth sets of SNPs used for evaluation.

Supplementary Dataset 2. Real sequencing datasets for evaluating bacterial SNP calling pipelines.

This is a complete archive to facilitate both the replication and expansion of this evaluation using real (REHAB project) sequencing data. It comprises 18 sets of paired-end reads and assemblies, the associated indexed reference genomes, SNP call truth sets, VCFs, and a suite of Perl scripts.

Declarations

Ethics approval and consent to participate

Not applicable.

Consent for publication

Not applicable.

Availability of data and material

All data analysed during this study are included in this published article and its supplementary information files. The simulated datasets generated during this study – comprising the SNP-containing genomes, log files of the SNPs introduced into each genome, and VCFs of strain-to-representative genome SNP calls – are available in Supplementary Dataset 1 (hosted online via the Oxford Research Archive at http://dx.doi.org/10.5287/bodleian:AmNXrjYN8).

Raw sequencing data and assemblies from the REHAB project, described in [61], are available in the NCBI under BioProject accession PRJNA42251 (https://www.ncbi.nlm.nih.gov/bioproject/PRJNA422511), with associated hybrid assemblies available via FigShare [118].

A complete archive to facilitate both the replication and expansion of this evaluation using the real (REHAB project) sequencing data is available as Supplementary Dataset 2 (hosted online via the Oxford Research Archive at https://ora.ox.ac.uk/objects/uuid:8f902497-955e-4b84-9b85-693ee0e4433e). This archive comprises 18 sets of paired-end reads and assemblies, the associated indexed reference genomes, SNP call truth sets, VCFs, and a suite of Perl scripts. These scripts are also available via https://github.com/oxfordmmm/GenomicDiversityPaper. Snapshots of this data and code are also available from the *GigaScience* GigaDB repository[121].

Availability of supporting source code and requirements

Project name: "Genomic diversity affects the accuracy of bacterial SNP calling pipelines".

Project home page: https://github.com/oxfordmmm/GenomicDiversityPaper.

Operating system(s): platform-independent.

Programming language: Perl (v5.22.1).

Other requirements: third-party software prerequisites are detailed in documentation provided with Supplementary Dataset 2 (https://ora.ox.ac.uk/objects/uuid:8f902497-955e-4b84-9b85-693ee0e4433e).

License: GNU GPL.

Competing interests

The authors declare that they have no competing interests.

Funding

This study was funded by the National Institute for Health Research Health Protection Research Unit (NIHR HPRU) in Healthcare Associated Infections and Antimicrobial Resistance at Oxford University in partnership with Public Health England (PHE) [grant HPRU-2012-10041]. DF, DWC, TEAP and ASW are supported by the NIHR Biomedical Research Centre. Computation used the Oxford Biomedical Research Computing (BMRC) facility, a joint development between the Wellcome Centre for Human Genetics and the Big Data Institute supported by Health Data Research UK and the NIHR Oxford Biomedical Research Centre. The report presents independent research funded by the National Institute for Health Research. The views expressed in this publication are those of the author and not necessarily those of the NHS, the National Institute for Health Research, the Department of Health or Public Health England. NS is funded by a University of Oxford/Public Health England Clinical Lectureship. LPS is funded by the Antimicrobial Resistance Cross Council Initiative supported by the seven research councils (NE/N019989/1). DWC, TEAP and ASW are NIHR Senior Investigators.

This work also made use of the Edinburgh Compute and Data Facility (ECDF) at the University of Edinburgh, supported in part by BBSRC Institute Strategic Program Grants awarded to The Roslin Institute including 'Control of Infectious Diseases' (BB/P013740/1).

Authors' contributions

SJB conceived of and designed the study with support from DF, DWE, TEAP, DWC and ASW. SJB performed all informatic analyses related to the SNP calling evaluation. ELC contributed to the acquisition of data and computational resources. NDM, LPS and NS generated and provided the reads and assemblies comprising the REHAB sequencing dataset. LPS created Figure 1. SJB wrote the manuscript, with edits from all other authors. All authors read and approved the final manuscript.

Acknowledgements

The authors would also like to thank the REHAB consortium, which currently includes (bracketed individuals in the main author list): Abuoun M, Anjum M, Bailey MJ, Barker L, Brett H, Bowes MJ, Chau K, (Crook DW), (De Maio N), Gilson D, Gweon HS, Hubbard ATM, Hoosdally S, Kavanagh J, Jones H, (Peto TEA), Read DS, Sebra R, (Shaw LP), Sheppard AE, Smith R, (Stoesser N), Stubberfield E, Swann J, (Walker AS), Wilson DJ, Woodford N.

References

1. Taylor AJ, Lappi V, Wolfgang WJ, Lapierre P, Palumbo MJ, Medus C, et al. Characterization of Foodborne Outbreaks of Salmonella enterica Serovar Enteritidis with Whole-Genome Sequencing Single Nucleotide Polymorphism-Based Analysis for Surveillance and Outbreak Detection. Journal of clinical microbiology. 2015;53 10:3334-40. doi:10.1128/jcm.01280-15.
2. Hendriksen RS, Price LB, Schupp JM, Gillece JD, Kaas RS, Engelthaler DM, et al. Population genetics of Vibrio cholerae from Nepal in 2010: evidence on the origin of the Haitian outbreak. mBio. 2011;2 4:e00157-11. doi:10.1128/mBio.00157-11.
3. Caspar SM, Dubacher N, Kopps AM, Meienberg J, Henggeler C and Matyas G. Clinical sequencing: From raw data to diagnosis with lifetime value. Clinical genetics. 2018;93 3:508-19. doi:10.1111/cge.13190.
4. Altmann A, Weber P, Bader D, Preuss M, Binder EB and Muller-Myhsok B. A beginners guide to SNP calling from high-throughput DNA-sequencing data. Human genetics. 2012;131 10:1541-54. doi:10.1007/s00439-012-1213-z.
5. Reinert K, Langmead B, Weese D and Evers DJ. Alignment of Next-Generation Sequencing Reads. Annual review of genomics and human genetics. 2015;16:133-51. doi:10.1146/annurev-genom-090413-025358.
6. Li H and Homer N. A survey of sequence alignment algorithms for next-generation sequencing. Brief Bioinform. 2010;11 5:473-83. doi:10.1093/bib/bbq015.
7. Mielczarek M and Szyda J. Review of alignment and SNP calling algorithms for next-generation sequencing data. Journal of Applied Genetics. 2016;57 1:71-9. doi:10.1007/s13353-015-0292-7.
8. Hasan MS, Wu X and Zhang L. Performance evaluation of indel calling tools using real short-read data. Human Genomics. 2015;9 1:20. doi:10.1186/s40246-015-0042-2.
9. O'Rawe J, Jiang T, Sun G, Wu Y, Wang W, Hu J, et al. Low concordance of multiple variant-calling pipelines: practical implications for exome and genome sequencing. Genome Medicine. 2013;5 3:28. doi:10.1186/gm432.
10. Alkan C, Coe BP and Eichler EE. Genome structural variation discovery and genotyping. Nature reviews Genetics. 2011;12 5:363-76. doi:10.1038/nrg2958.
11. Sandmann S, de Graaf AO, Karimi M, van der Reijden BA, Hellstrom-Lindberg E, Jansen JH, et al. Evaluating Variant Calling Tools for Non-Matched Next-Generation Sequencing Data. Sci Rep. 2017;7:43169. doi:10.1038/srep43169.
12. Liu X, Han S, Wang Z, Gelernter J and Yang B-Z. Variant Callers for Next-Generation Sequencing Data: A Comparison Study. PLoS ONE. 2013;8 9:e75619. doi:10.1371/journal.pone.0075619.
13. Li H. Toward better understanding of artifacts in variant calling from high-coverage samples. Bioinformatics. 2014;30 20:2843-51. doi:10.1093/bioinformatics/btu356.
14. Hwang S, Kim E, Lee I and Marcotte EM. Systematic comparison of variant calling pipelines using gold standard personal exome variants. Scientific Reports. 2015;5:17875. doi:10.1038/srep17875.
15. Cornish A and Guda C. A Comparison of Variant Calling Pipelines Using Genome in a Bottle as a Reference. BioMed Research International. 2015;2015:11. doi:10.1155/2015/456479.
16. Smith HE and Yun S. Evaluating alignment and variant-calling software for mutation identification in C. elegans by whole-genome sequencing. PLoS ONE. 2017;12 3:e0174446. doi:10.1371/journal.pone.0174446.

17. Baes CF, Dolezal MA, Koltes JE, Bapst B, Fritz-Waters E, Jansen S, et al. Evaluation of variant identification methods for whole genome sequencing data in dairy cattle. BMC Genomics. 2014;15 1:948. doi:10.1186/1471-2164-15-948.
18. Mielczarek M and Szyda J. Review of alignment and SNP calling algorithms for next-generation sequencing data. Journal of applied genetics. 2016;57 1:71-9. doi:10.1007/s13353-015-0292-7.
19. Eberle MA, Fritzilas E, Krusche P, Källberg M, Moore BL, Bekritsky MA, et al. A reference data set of 5.4 million phased human variants validated by genetic inheritance from sequencing a three-generation 17-member pedigree. Genome Research. 2016; doi:10.1101/gr.210500.116.
20. Kómár P and Kural D. geck: trio-based comparative benchmarking of variant calls. Bioinformatics. 2018:bty415-bty. doi:10.1093/bioinformatics/bty415.
21. Li H, Handsaker B, Wysoker A, Fennell T, Ruan J, Homer N, et al. The Sequence Alignment/Map format and SAMtools. Bioinformatics. 2009;25 16:2078-9. doi:10.1093/bioinformatics/btp352.
22. Pightling AW, Petronella N and Pagotto F. Choice of Reference Sequence and Assembler for Alignment of Listeria monocytogenes Short-Read Sequence Data Greatly Influences Rates of Error in SNP Analyses. PLoS ONE. 2014;9 8:e104579. doi:10.1371/journal.pone.0104579.
23. Li H and Durbin R. Fast and accurate short read alignment with Burrows–Wheeler transform. Bioinformatics. 2009;25 14:1754-60. doi:10.1093/bioinformatics/btp324.
24. DePristo MA, Banks E, Poplin RE, Garimella KV, Maguire JR, Hartl C, et al. A framework for variation discovery and genotyping using next-generation DNA sequencing data. Nature genetics. 2011;43 5:491-8. doi:10.1038/ng.806.
25. McKenna A, Hanna M, Banks E, Sivachenko A, Cibulskis K, Kernytsky A, et al. The Genome Analysis Toolkit: A MapReduce framework for analyzing next-generation DNA sequencing data. Genome Research. 2010;20 9:1297-303. doi:10.1101/gr.107524.110.
26. Cornish A and Guda C. A Comparison of Variant Calling Pipelines Using Genome in a Bottle as a Reference. BioMed Research International. 2015;2015:456479. doi:10.1155/2015/456479.
27. Canzar S and Salzberg SL. Short Read Mapping: An Algorithmic Tour. Proc IEEE Inst Electr Electron Eng. 2017;105 3:436-58. doi:10.1109/JPROC.2015.2455551.
28. Langmead B and Salzberg SL. Fast gapped-read alignment with Bowtie 2. Nature methods. 2012;9 4:357-9. doi:10.1038/nmeth.1923.
29. Liu Y, Popp B and Schmidt B. CUSHAW3: sensitive and accurate base-space and color-space short-read alignment with hybrid seeding. PLoS One. 2014;9 1:e86869. doi:10.1371/journal.pone.0086869.
30. Rizk G and Lavenier D. GASSST: global alignment short sequence search tool. Bioinformatics. 2010;26 20:2534-40. doi:10.1093/bioinformatics/btq485.
31. Marco-Sola S, Sammeth M, Guigo R and Ribeca P. The GEM mapper: fast, accurate and versatile alignment by filtration. Nat Methods. 2012;9 12:1185-8.
32. Kim D, Langmead B and Salzberg SL. HISAT: a fast spliced aligner with low memory requirements. Nature methods. 2015;12:357. doi:10.1038/nmeth.3317.
33. Li H. Minimap2: pairwise alignment for nucleotide sequences. Bioinformatics. 2018:bty191-bty. doi:10.1093/bioinformatics/bty191.
34. Lee WP, Stromberg MP, Ward A, Stewart C, Garrison EP and Marth GT. MOSAIK: a hash-based algorithm for accurate next-generation sequencing short-read mapping. PLoS One. 2014;9 3:e90581. doi:10.1371/journal.pone.0090581.

35. Sedlazeck FJ, Rescheneder P and von Haeseler A. NextGenMap: fast and accurate read mapping in highly polymorphic genomes. Bioinformatics. 2013;29 21:2790-1. doi:10.1093/bioinformatics/btt468.
36. Zaharia M, Bolosky WJ, Curtis K, Fox A, Patterson D, Shenker S, et al. Faster and more accurate sequence alignment with SNAP. arXiv preprint arXiv:11115572. 2011.
37. Lunter G and Goodson M. Stampy: A statistical algorithm for sensitive and fast mapping of Illumina sequence reads. Genome Research. 2011;21 6:936-9. doi:10.1101/gr.111120.110.
38. Siragusa E, Weese D and Reinert K. Fast and accurate read mapping with approximate seeds and multiple backtracking. Nucleic Acids Res. 2013;41 7:e78. doi:10.1093/nar/gkt005.
39. Luo R, Schatz MC and Salzberg SL. 16GT: a fast and sensitive variant caller using a 16-genotype probabilistic model. GigaScience. 2017;6 7:1-4. doi:10.1093/gigascience/gix045.
40. Poplin R, Chang P-C, Alexander D, Schwartz S, Colthurst T, Ku A, et al. A universal SNP and small-indel variant caller using deep neural networks. Nature Biotechnology. 2018;36:983. doi:10.1038/nbt.4235.
41. Garrison E and Marth G. Haplotype-based variant detection from short-read sequencing. arXiv. 2012:arXiv:1207.3907 [q-bio.GN].
42. Wilm A, Aw PPK, Bertrand D, Yeo GHT, Ong SH, Wong CH, et al. LoFreq: a sequence-quality aware, ultra-sensitive variant caller for uncovering cell-population heterogeneity from high-throughput sequencing datasets. Nucleic Acids Research. 2012;40 22:11189-201. doi:10.1093/nar/gks918.
43. Cooke DP, Wedge DC and Lunter G. A unified haplotype-based method for accurate and comprehensive variant calling. bioRxiv. 2018:456103. doi:10.1101/456103.
44. Walker BJ, Abeel T, Shea T, Priest M, Abouelliel A, Sakthikumar S, et al. Pilon: An Integrated Tool for Comprehensive Microbial Variant Detection and Genome Assembly Improvement. PLoS ONE. 2014;9 11:e112963. doi:10.1371/journal.pone.0112963.
45. Rimmer A, Phan H, Mathieson I, Iqbal Z, Twigg SRF, Consortium WGS, et al. Integrating mapping-, assembly- and haplotype-based approaches for calling variants in clinical sequencing applications. Nature Genetics. 2014;46:912. doi:10.1038/ng.3036.
46. Wei Z, Wang W, Hu P, Lyon GJ and Hakonarson H. SNVer: a statistical tool for variant calling in analysis of pooled or individual next-generation sequencing data. Nucleic Acids Res. 2011;39 19:e132. doi:10.1093/nar/gkr599.
47. Liu Y, Loewer M, Aluru S and Schmidt B. SNVSniffer: an integrated caller for germline and somatic single-nucleotide and indel mutations. BMC Systems Biology. 2016;10 2:47. doi:10.1186/s12918-016-0300-5.
48. Saunders CT, Wong WS, Swamy S, Becq J, Murray LJ and Cheetham RK. Strelka: accurate somatic small-variant calling from sequenced tumor-normal sample pairs. Bioinformatics. 2012;28 14:1811-7. doi:10.1093/bioinformatics/bts271.
49. Koboldt DC, Chen K, Wylie T, Larson DE, McLellan MD, Mardis ER, et al. VarScan: variant detection in massively parallel sequencing of individual and pooled samples. Bioinformatics. 2009;25 17:2283-5. doi:10.1093/bioinformatics/btp373.
50. Sarovich DS and Price EP. SPANDx: a genomics pipeline for comparative analysis of large haploid whole genome re-sequencing datasets. BMC research notes. 2014;7:618. doi:10.1186/1756-0500-7-618.

51. Chiang C, Layer RM, Faust GG, Lindberg MR, Rose DB, Garrison EP, et al. SpeedSeq: ultra-fast personal genome analysis and interpretation. Nature methods. 2015;12:966. doi:10.1038/nmeth.3505.
52. Lawson PA, Citron DM, Tyrrell KL and Finegold SM. Reclassification of Clostridium difficile as Clostridioides difficile (Hall and O'Toole 1935) Prevot 1938. Anaerobe. 2016;40:95-9. doi:10.1016/j.anaerobe.2016.06.008.
53. Sebaihia M, Wren BW, Mullany P, Fairweather NF, Minton N, Stabler R, et al. The multidrug-resistant human pathogen Clostridium difficile has a highly mobile, mosaic genome. Nat Genet. 2006;38 7:779-86. doi:10.1038/ng1830.
54. Wick RR, Judd LM, Gorrie CL and Holt KE. Unicycler: Resolving bacterial genome assemblies from short and long sequencing reads. PLoS computational biology. 2017;13 6:e1005595. doi:10.1371/journal.pcbi.1005595.
55. Sandmann S, de Graaf AO, Karimi M, van der Reijden BA, Hellström-Lindberg E, Jansen JH, et al. Evaluating Variant Calling Tools for Non-Matched Next-Generation Sequencing Data. Scientific Reports. 2017;7:43169. doi:10.1038/srep43169.
56. Marçais G, Delcher AL, Phillippy AM, Coston R, Salzberg SL and Zimin A. MUMmer4: A fast and versatile genome alignment system. PLoS Computational Biology. 2018;14 1:e1005944. doi:10.1371/journal.pcbi.1005944.
57. Treangen TJ, Ondov BD, Koren S and Phillippy AM. The Harvest suite for rapid core-genome alignment and visualization of thousands of intraspecific microbial genomes. Genome Biology. 2014;15 11:524. doi:10.1186/s13059-014-0524-x.
58. Ondov BD, Treangen TJ, Melsted P, Mallonee AB, Bergman NH, Koren S, et al. Mash: fast genome and metagenome distance estimation using MinHash. Genome Biology. 2016;17 1:132. doi:10.1186/s13059-016-0997-x.
59. Cliff N. Dominance statistics: Ordinal analyses to answer ordinal questions. Psychological Bulletin. 1993;114 3:494-509.
60. Macbeth G, Razumiejczyk E and Ledesma RD. Cliff's delta calculator: a non-parametric effect size program for two groups of observations. Universitas Psychologica. 2011;10 2:545-55.
61. De Maio N, Shaw LP, Hubbard A, George S, Sanderson ND, Swann J, et al. Comparison of long-read sequencing technologies in the hybrid assembly of complex bacterial genomes. Microb Genom. 2019;5 9:e000294. doi:10.1099/mgen.0.000294.
62. Zook J, McDaniel J, Parikh H, Heaton H, Irvine SA, Trigg L, et al. Reproducible integration of multiple sequencing datasets to form high-confidence SNP, indel, and reference calls for five human genome reference materials. bioRxiv. 2018.
63. Zook JM, Catoe D, McDaniel J, Vang L, Spies N, Sidow A, et al. Extensive sequencing of seven human genomes to characterize benchmark reference materials. Scientific Data. 2016;3:160025. doi:10.1038/sdata.2016.25.
64. Zook JM and Salit M. Genomes in a bottle: creating standard reference materials for genomic variation - why, what and how? Genome Biology. 2011;12 Suppl 1:P31-P. doi:10.1186/gb-2011-12-s1-p31.
65. Tian S, Yan H, Neuhauser C and Slager SL. An analytical workflow for accurate variant discovery in highly divergent regions. BMC Genomics. 2016;17 1:703. doi:10.1186/s12864-016-3045-z.
66. van Eijk E, Anvar SY, Browne HP, Leung WY, Frank J, Schmitz AM, et al. Complete genome sequence of the Clostridium difficile laboratory strain 630Δerm reveals differences from strain 630, including translocation of the mobile element CTn5. BMC Genomics. 2015;16 1:31. doi:10.1186/s12864-015-1252-7.

67. Jordan PW, Snyder LA and Saunders NJ. Strain-specific differences in Neisseria gonorrhoeae associated with the phase variable gene repertoire. BMC Microbiology. 2005;5 1:21. doi:10.1186/1471-2180-5-21.
68. Hanage WP. Fuzzy species revisited. BMC Biology. 2013;11 1:41. doi:10.1186/1741-7007-11-41.
69. Hanage WP, Fraser C and Spratt BG. Fuzzy species among recombinogenic bacteria. BMC biology. 2005;3:6-. doi:10.1186/1741-7007-3-6.
70. Dos Vultos T, Mestre O, Rauzier J, Golec M, Rastogi N, Rasolofo V, et al. Evolution and diversity of clonal bacteria: the paradigm of Mycobacterium tuberculosis. PLoS One. 2008;3 2:e1538. doi:10.1371/journal.pone.0001538.
71. Lee RS and Behr MA. Does Choice Matter? Reference-Based Alignment for Molecular Epidemiology of Tuberculosis. Journal of clinical microbiology. 2016;54 7:1891-5. doi:10.1128/jcm.00364-16.
72. Nadon CA, Woodward DL, Young C, Rodgers FG and Wiedmann M. Correlations between molecular subtyping and serotyping of Listeria monocytogenes. Journal of clinical microbiology. 2001;39 7:2704-7. doi:10.1128/jcm.39.7.2704-2707.2001.
73. Rasmussen OF, Skouboe P, Dons L, Rossen L and Olsen JE. Listeria monocytogenes exists in at least three evolutionary lines: evidence from flagellin, invasive associated protein and listeriolysin O genes. Microbiology (Reading, England). 1995;141 (Pt 9):2053-61. doi:10.1099/13500872-141-9-2053.
74. Pirone-Davies C, Chen Y, Pightling A, Ryan G, Wang Y, Yao K, et al. Genes significantly associated with lineage II food isolates of Listeria monocytogenes. BMC Genomics. 2018;19 1:708. doi:10.1186/s12864-018-5074-2.
75. Olson ND, Lund SP, Colman RE, Foster JT, Sahl JW, Schupp JM, et al. Best practices for evaluating single nucleotide variant calling methods for microbial genomics. Frontiers in Genetics. 2015;6:235. doi:10.3389/fgene.2015.00235.
76. Price A and Gibas C. The quantitative impact of read mapping to non-native reference genomes in comparative RNA-Seq studies. PLoS ONE. 2017;12 7:e0180904. doi:10.1371/journal.pone.0180904.
77. Walker TM, Ip CLC, Harrell RH, Evans JT, Kapatai G, Dedicoat MJ, et al. Whole-genome sequencing to delineate Mycobacterium tuberculosis outbreaks: a retrospective observational study. The Lancet Infectious diseases. 2013;13 2:137-46. doi:10.1016/S1473-3099(12)70277-3.
78. Long Q, Rabanal FA, Meng D, Huber CD, Farlow A, Platzer A, et al. Massive genomic variation and strong selection in Arabidopsis thaliana lines from Sweden. Nature genetics. 2013;45 8:884-90. doi:10.1038/ng.2678.
79. Ghoneim DH, Myers JR, Tuttle E and Paciorkowski AR. Comparison of insertion/deletion calling algorithms on human next-generation sequencing data. BMC research notes. 2014;7 1:864. doi:10.1186/1756-0500-7-864.
80. Farrer RA, Henk DA, MacLean D, Studholme DJ and Fisher MC. Using false discovery rates to benchmark SNP-callers in next-generation sequencing projects. Sci Rep. 2013;3:1512. doi:10.1038/srep01512.
81. Liu Q, Guo Y, Li J, Long J, Zhang B and Shyr Y. Steps to ensure accuracy in genotype and SNP calling from Illumina sequencing data. BMC Genomics. 2012;13 Suppl 8:S8. doi:10.1186/1471-2164-13-s8-s8.
82. Glenn TC. Field guide to next-generation DNA sequencers. Molecular Ecology Resources. 2011;11 5:759-69. doi:10.1111/j.1755-0998.2011.03024.x.
83. Kim D, Song L, Breitwieser FP and Salzberg SL. Centrifuge: rapid and sensitive classification of metagenomic sequences. Genome Res. 2016;26 12:1721-9. doi:10.1101/gr.210641.116.

84. Ounit R and Lonardi S. Higher classification sensitivity of short metagenomic reads with CLARK-S. Bioinformatics. 2016;32 24:3823-5. doi:10.1093/bioinformatics/btw542.
85. Menzel P, Ng KL and Krogh A. Fast and sensitive taxonomic classification for metagenomics with Kaiju. Nature communications. 2016;7:11257. doi:10.1038/ncomms11257.
86. Davis MP, van Dongen S, Abreu-Goodger C, Bartonicek N and Enright AJ. Kraken: a set of tools for quality control and analysis of high-throughput sequence data. Methods. 2013;63 1:41-9. doi:10.1016/j.ymeth.2013.06.027.
87. Mende DR, Letunic I, Huerta-Cepas J, Li SS, Forslund K, Sunagawa S, et al. proGenomes: a resource for consistent functional and taxonomic annotations of prokaryotic genomes. Nucleic Acids Research. 2017;45 Database issue:D529-D34. doi:10.1093/nar/gkw989.
88. O'Leary NA, Wright MW, Brister JR, Ciufo S, Haddad D, McVeigh R, et al. Reference sequence (RefSeq) database at NCBI: current status, taxonomic expansion, and functional annotation. Nucleic Acids Research. 2016;44 Database issue:D733-D45. doi:10.1093/nar/gkv1189.
89. McIntyre ABR, Ounit R, Afshinnekoo E, Prill RJ, Hénaff E, Alexander N, et al. Comprehensive benchmarking and ensemble approaches for metagenomic classifiers. Genome Biology. 2017;18 1:182. doi:10.1186/s13059-017-1299-7.
90. Lindgreen S, Adair KL and Gardner PP. An evaluation of the accuracy and speed of metagenome analysis tools. Scientific Reports. 2016;6:19233. doi:10.1038/srep19233.
91. Bradley P, Gordon NC, Walker TM, Dunn L, Heys S, Huang B, et al. Rapid antibiotic-resistance predictions from genome sequence data for Staphylococcus aureus and Mycobacterium tuberculosis. Nature communications. 2015;6:10063. doi:10.1038/ncomms10063.
92. Lan R and Reeves PR. Escherichia coli in disguise: molecular origins of Shigella. Microbes and infection. 2002;4 11:1125-32.
93. Baker DN and Langmead B. Dashing: Fast and Accurate Genomic Distances with HyperLogLog. bioRxiv. 2019:501726. doi:10.1101/501726.
94. Jain C, Rodriguez-R LM, Phillippy AM, Konstantinidis KT and Aluru S. High throughput ANI analysis of 90K prokaryotic genomes reveals clear species boundaries. Nature Communications. 2018;9 1:5114. doi:10.1038/s41467-018-07641-9.
95. Koren S and Phillippy AM. One chromosome, one contig: complete microbial genomes from long-read sequencing and assembly. Current opinion in microbiology. 2015;23:110-20. doi:10.1016/j.mib.2014.11.014.
96. Hurgobin B and Edwards D. SNP Discovery Using a Pangenome: Has the Single Reference Approach Become Obsolete? Biology. 2017;6 1:21. doi:10.3390/biology6010021.
97. Page AJ, Cummins CA, Hunt M, Wong VK, Reuter S, Holden MTG, et al. Roary: rapid large-scale prokaryote pan genome analysis. Bioinformatics. 2015;31 22:3691-3. doi:10.1093/bioinformatics/btv421.
98. Bertels F, Silander OK, Pachkov M, Rainey PB and van Nimwegen E. Automated reconstruction of whole-genome phylogenies from short-sequence reads. Mol Biol Evol. 2014;31 5:1077-88. doi:10.1093/molbev/msu088.
99. Sahl JW, Lemmer D, Travis J, Schupp JM, Gillece JD, Aziz M, et al. NASP: an accurate, rapid method for the identification of SNPs in WGS datasets that supports flexible input and output formats. Microb Genom. 2016;2 8:e000074-e. doi:10.1099/mgen.0.000074.

100. Fang LT, Afshar PT, Chhibber A, Mohiyuddin M, Fan Y, Mu JC, et al. An ensemble approach to accurately detect somatic mutations using SomaticSeq. Genome Biology. 2015;16 1:197-. doi:10.1186/s13059-015-0758-2.
101. Teer JK, Green ED, Mullikin JC and Biesecker LG. VarSifter: visualizing and analyzing exome-scale sequence variation data on a desktop computer. Bioinformatics. 2012;28 4:599-600. doi:10.1093/bioinformatics/btr711.
102. Demirci H and Akgün M. VCF-Explorer: filtering and analysing whole genome VCF files. Bioinformatics. 2017;33 21:3468-70. doi:10.1093/bioinformatics/btx422.
103. Müller H, Jimenez-Heredia R, Krolo A, Hirschmugl T, Dmytrus J, Boztug K, et al. VCF.Filter: interactive prioritization of disease-linked genetic variants from sequencing data. Nucleic acids research. 2017;45 W1:W567-W72. doi:10.1093/nar/gkx425.
104. Ramraj V and Salatino S. BrowseVCF: a web-based application and workflow to quickly prioritize disease-causative variants in VCF files. Briefings in Bioinformatics. 2016;18 5:774-9. doi:10.1093/bib/bbw054.
105. Olm MR, Crits-Christoph A, Diamond S, Lavy A, Matheus Carnevali PB and Banfield JF. Consistent metagenome-derived metrics verify and define bacterial species boundaries. bioRxiv. 2019:647511. doi:10.1101/647511.
106. NCBI Resource Coordinators. Database Resources of the National Center for Biotechnology Information. Nucleic Acids Res. 2017;45 D1:D12-d7. doi:10.1093/nar/gkw1071.
107. Kumar S, Stecher G and Tamura K. MEGA7: Molecular Evolutionary Genetics Analysis Version 7.0 for Bigger Datasets. Mol Biol Evol. 2016;33 7:1870-4. doi:10.1093/molbev/msw054.
108. Price A and Gibas C. Simulome: a genome sequence and variant simulator. Bioinformatics. 2017; doi:10.1093/bioinformatics/btx091.
109. Turner FS. Assessment of insert sizes and adapter content in fastq data from NexteraXT libraries. Frontiers in Genetics. 2014;5:5. doi:10.3389/fgene.2014.00005.
110. Turner FS. Assessment of insert sizes and adapter content in fastq data from NexteraXT libraries. Frontiers in genetics. 2014;5:5-. doi:10.3389/fgene.2014.00005.
111. Broad Institute: Picard: A set of command line tools (in Java) for manipulating high-throughput sequencing (HTS) data and formats such as SAM/BAM/CRAM and VCF. http://broadinstitute.github.io/picard/ (2018).
112. Eyre DW, Cule ML, Wilson DJ, Griffiths D, Vaughan A, O'Connor L, et al. Diverse Sources of C. difficile Infection Identified on Whole-Genome Sequencing. New England Journal of Medicine. 2013;369 13:1195-205. doi:10.1056/NEJMoa1216064.
113. Jia P, Li F, Xia J, Chen H, Ji H, Pao W, et al. Consensus rules in variant detection from next-generation sequencing data. PLoS ONE. 2012;7 6:e38470-e. doi:10.1371/journal.pone.0038470.
114. Wood DE and Salzberg SL. Kraken: ultrafast metagenomic sequence classification using exact alignments. Genome Biology. 2014;15 3:R46. doi:10.1186/gb-2014-15-3-r46.
115. Mende DR, Letunic I, Huerta-Cepas J, Li SS, Forslund K, Sunagawa S, et al. proGenomes: a resource for consistent functional and taxonomic annotations of prokaryotic genomes. Nucleic acids research. 2017;45 D1:D529-D34. doi:10.1093/nar/gkw989.
116. O'Leary NA, Wright MW, Brister JR, Ciufo S, Haddad D, McVeigh R, et al. Reference sequence (RefSeq) database at NCBI: current status, taxonomic expansion, and functional annotation. Nucleic Acids Res. 2016;44 D1:D733-45. doi:10.1093/nar/gkv1189.

117. Koren S, Walenz BP, Berlin K, Miller JR, Bergman NH and Phillippy AM. Canu: scalable and accurate long-read assembly via adaptive k-mer weighting and repeat separation. Genome Research. 2017;27 5:722-36. doi:10.1101/gr.215087.116.
118. Shaw, Liam; De Maio, Nicola; Consortium, The REHAB (2019): Hybrid Enterobacteriaceae assemblies using PacBio+Illumina or ONT+Illumina sequencing. figshare. Dataset. https://doi.org/10.6084/m9.figshare.7649051
119. Torchiano M: effsize: Efficient Effect Size Computation (R package version 0.5.4). http://cran.r-project.org/web/packages/effsize/index.html (2015).
120. Romano J, Kromrey JD, Coraggio J and Skowronek J. Appropriate statistics for ordinal level data: should we really be using t-test and Cohen's d for evaluating group differences on the NSSE and other surveys? *Annual Meeting of the Florida Association of Institutional Research*. Cocoa Beach, Florida, USA2006.

121. Bush SJ; Foster D; Eyre DW; Clark EL; De Maio N; Shaw LP; Stoesser N; Peto TEA; Crook DW; Walker AS (2020): Supporting data for "Genomic diversity affects the accuracy of bacterial SNP calling pipelines" GigaScience Database. http://dx.doi.org/10.5524/100694

Click here to access/download;Table;Table 1.xlsx

Table 1. Summary of pipeline performance across all s

Performance measure
F-score
Precision (specificity)
Recall (sensitivity)
No. of true positive calls
No. of false positive calls
No. of false negative calls
Total no. of errors (FP + FN calls) per million sequenced bases
Sum of ranks for all previous measures

Numbers in parentheses refer to the median value, across all simulatio

Snippy is based upon a BWA-mem/freebayes pipeline, although under

pecies' genomes.

Top ranked pipeline(s) (when the reference genome is the same as the source of the reads)
bwa-mem with freebayes/gatk, minimap2 with freebayes/gatk, novoalign/gatk, stampy/gatk (0.994)
snippy, bwa-mem/minimap2/novoalign/stampy with 16GT/freebayes/gatk/lofreq/mpileup/platypus/snver /strelka/varscan (1.000)
bwa-mem/novoalign/stampy with gatk (0.989)
novoalign/gatk (15,777)
stampy with mpileup/platypus (0.000)
novoalign/gatk (0.941)
novoalign/gatk (0.944)
novoalign/gatk (10)

ons, for each performance measure.
default parameters shows improved performance. Wh

Top ranked pipeline(s) (when the reference genome is divergent from the reads)
snippy (0.982) *
novoalign/snvsniffer (0.971)
bwa-mem with 16GT/freebayes, stampy/freebayes (0.997)
bwa-mem/freebayes (13,829)
novoalign/snvsniffer (1.825)
bwa-mem/freebayes (0.188)
snippy (2.627) *
snippy (20) *

ıen the reference genome diverges from the reads and compared to

Top ranked pipeline(s) (averaged across all simulations)

---

novoalign with lofreq/mpileup, snippy (0.986)

novoalign/snvsniffer (0.986)

bwa-mem/minimap2/stampy with freebayes (0.992)
bwa-mem/freebayes (14,791)
novoalign/snvsniffer (0.913)
bwa-mem/freebayes (0.641)
snippy (2.125)
novoalign/mpileup (42)

the rank 1 position of Snippy, BWA-mem/freebayes has a median F-score of 0.965 (ranking 12 out o

f 41 pipelines), a median number of errors per million sequenced bases of 5.265 (ranking 26 out of 41 pip

ɔelines), and a sum of ranks of 98.

Table 2. Overall performance of each pipeline per species, calculated as the

Pipeline	*Clostridiodes difficile*	*Escherichia coli*	*Klebsiella pneumoniae*	*Listeria monocytogenes*
snippy *	2	1	1	1
novoalign/lofreq	1	2	3	10
novoalign/mpileup	3	3	4	9
novoalign/16GT	5	5	6	8
novoalign/snver	4	4	5	12
minimap2/mpileup	10	6	2	20
novoalign/strelka	6	9	13	7
bwa-mem/mpileup	12	14	15	2
minimap2/strelka	8	11	10	21
bwa-mem/snver	9	10	11	5
minimap2/lofreq	20	8	7	18
novoalign/freebayes	7	13	12	14
bwa-mem/16GT	22	18	20	6
bwa-mem/strelka	16	25	22	4
bwa-mem/lofreq	18	16	19	3
minimap2/freebayes	14	12	9	15
minimap2/16GT	21	15	14	16
minimap2/snver	11	7	8	25
bwa-mem/freebayes *	15	17	16	13
novoalign/varscan	13	19	17	17
bwa-mem/varscan	17	24	21	11
bwa-mem/platypus	31	23	25	19
stampy/strelka	24	27	27	22
minimap2/varscan	19	21	18	29
novoalign/platypus	29	20	23	23
minimap2/platypus	23	22	24	34
stampy/freebayes	26	26	26	24
bwa-mem/gatk	27	28	32	26
stampy/mpileup	36	32	29	28
novoalign/gatk	28	29	31	27
stampy/lofreq	37	33	30	30
minimap2/gatk	25	31	33	33
stampy/gatk	34	34	35	31
stampy/platypus	38	35	39	35
novoalign/snvsniffer	33	30	28	32
stampy/snver	30	39	34	41
bwa-mem/snvsniffer	32	36	36	38
stampy/16GT	40	38	37	37
stampy/varscan	41	40	38	39
minimap2/snvsniffer	35	37	40	40
stampy/snvsniffer	39	41	41	36

* Snippy is based upon a BWA-mem/freebayes pipeline but under default parameters, shows im

e sum of seven ranks, when reads are aligned to a divergent genome.

Mycobacterium tuberculosis	*Neisseria gonorrhoea*	*Salmonella enterica*	*Shigella dysenteriae*	*Staphylococcus aureus*	*Streptococcus pneumoniae*
5	1	1	2	1	1
3	4	2	1	3	2
2	10	5	4	2	3
8	12	3	18	6	6
12	14	4	14	4	10
9	13	9	9	7	15
13	27	8	11	11	4
7	8	19	17	8	9
15	6	11	12	10	7
21	2	10	21	14	12
10	17	18	3	9	14
1	22	6	24	18	17
19	15	17	5	13	8
16	5	26	7	17	5
11	20	24	19	5	11
4	25	7	23	19	18
18	18	16	6	12	13
22	3	12	26	15	22
6	19	13	16	21	16
20	16	15	13	16	21
30	9	23	29	23	23
36	7	22	10	24	20
25	11	32	15	20	19
32	26	21	31	22	25
28	32	14	25	30	27
34	21	20	22	25	29
33	30	29	30	26	24
26	31	28	28	27	26
14	23	35	27	31	30
23	34	25	34	28	31
17	29	37	20	32	32
24	35	27	35	34	28
27	37	30	32	33	34
37	24	33	8	41	39
38	33	31	38	36	33
29	28	40	37	38	35
39	39	34	39	29	38
35	36	39	33	39	36
31	38	41	36	40	37
40	40	36	40	35	40
41	41	38	41	37	41

proved performance.

Sum of ranks	Range of ranks
16	4
31	9
45	8
77	15
83	10
100	18
109	23
111	17
111	15
115	19
124	17
134	23
143	17
143	22
146	21
146	21
149	15
151	23
152	15
167	8
210	21
217	29
222	21
244	14
251	18
254	14
274	9
279	6
285	22
290	11
297	20
305	11
327	10
329	33
332	10
351	13
360	10
370	7
381	10
383	5
396	5

Figure 1

Click here to access/download;Figure;Figure 1.pdf

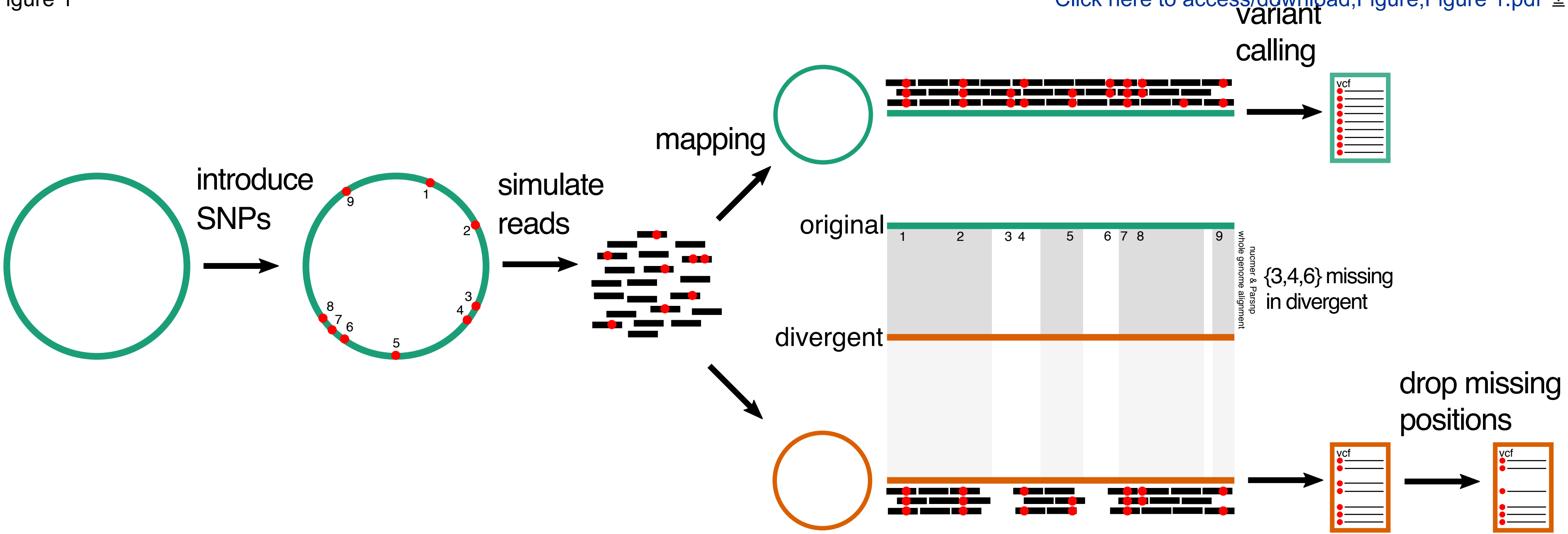

Figure 2

Click here to access/download;Figure;Figure 2.png

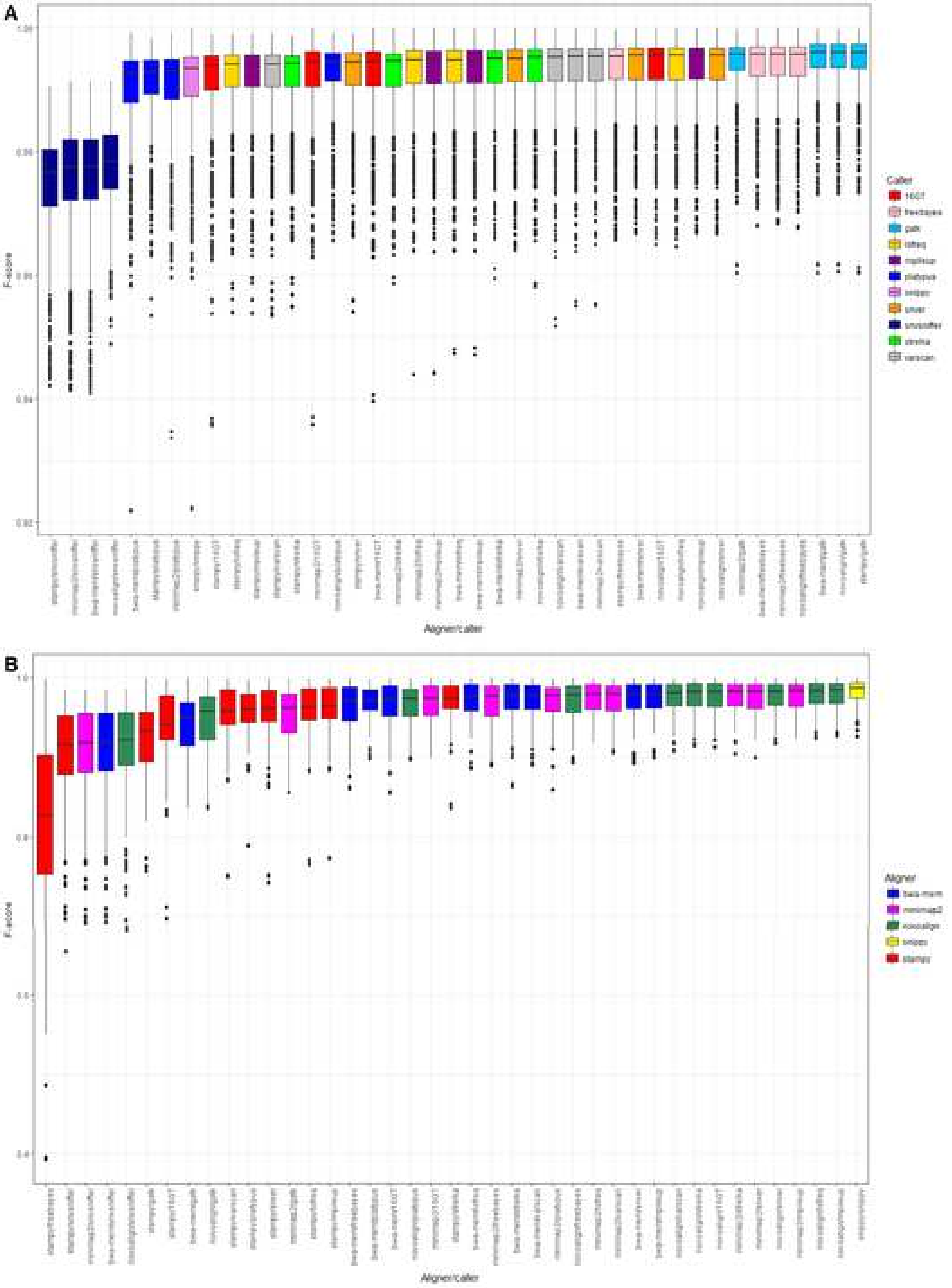

Figure 3

Click here to access/download;Figure;Figure 3.pdf

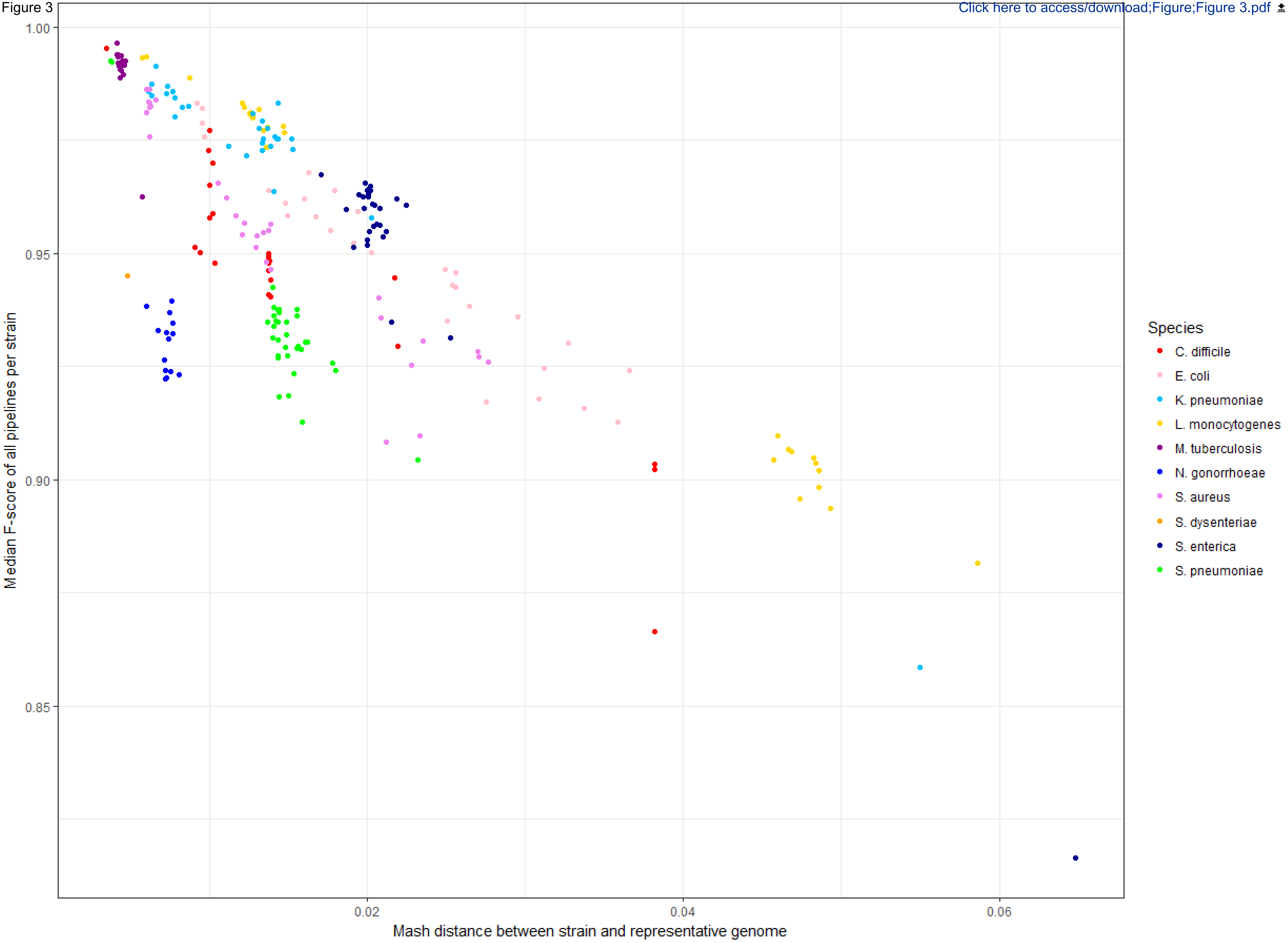

Figure 4

Click here to access/download;Figure;Figure 4.pdf

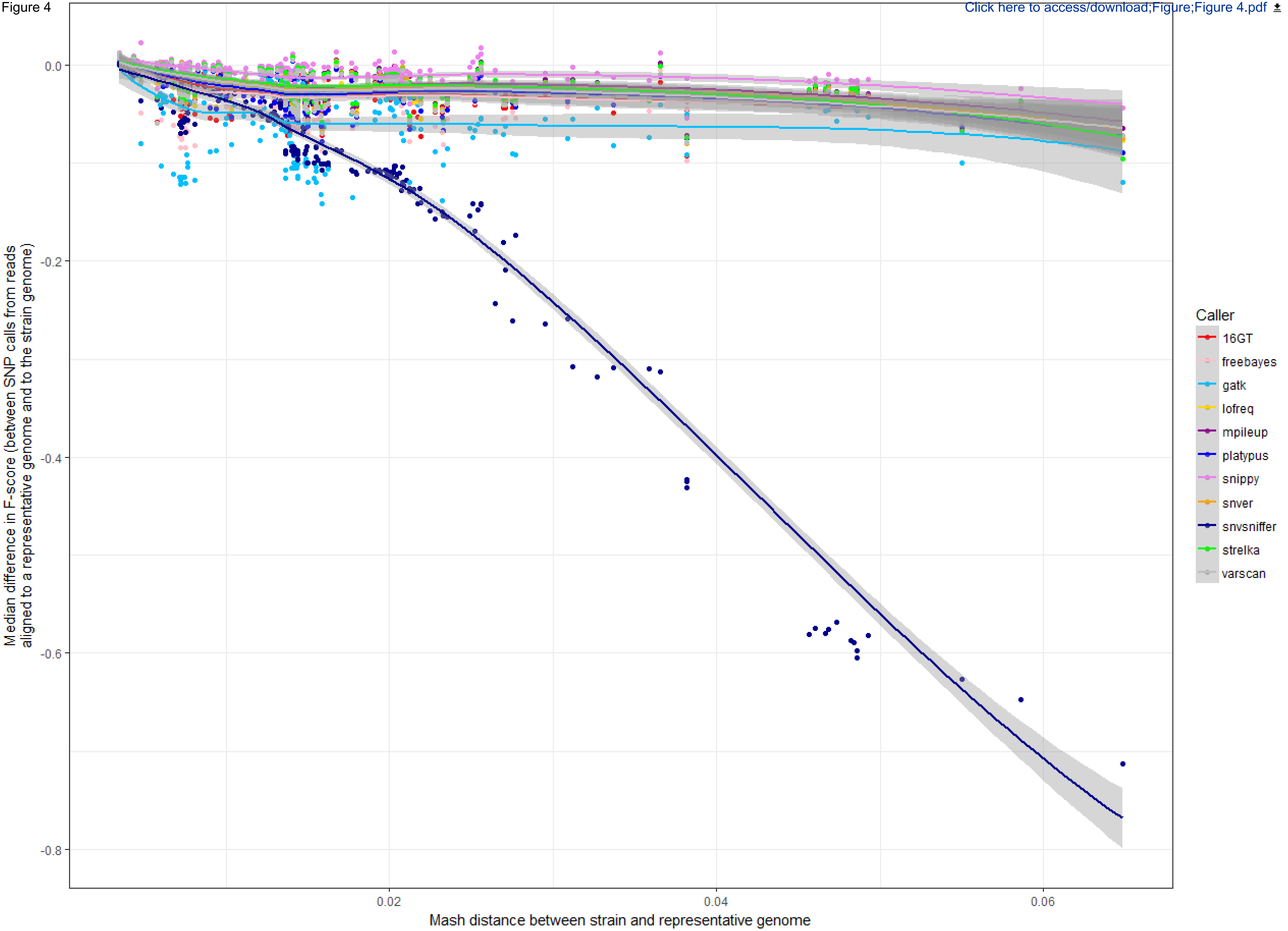

Figure 5

Click here to access/download;Figure;Figure 5.pdf

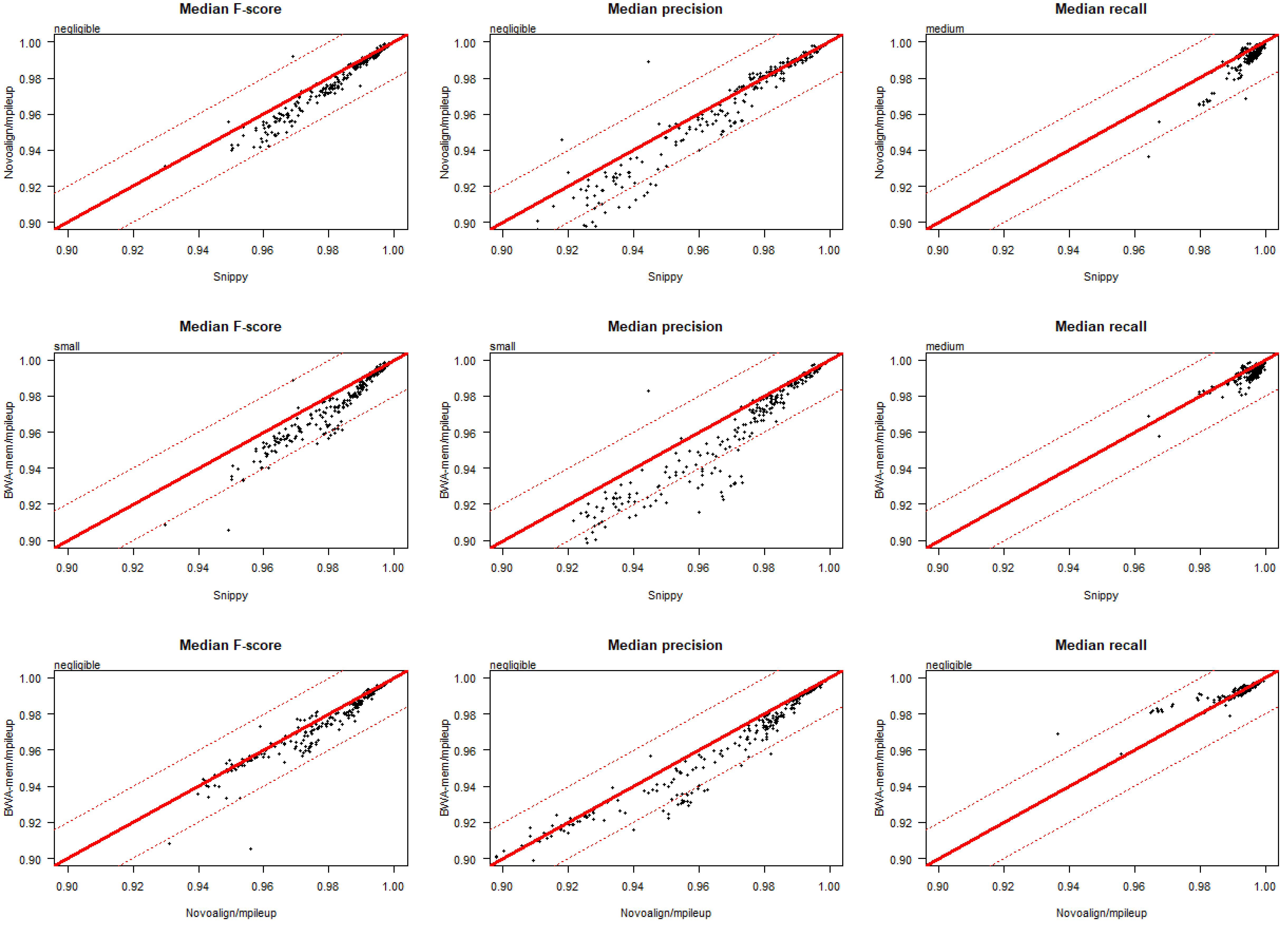

Figure 6

Click here to access/download;Figure;Figure 6.pdf

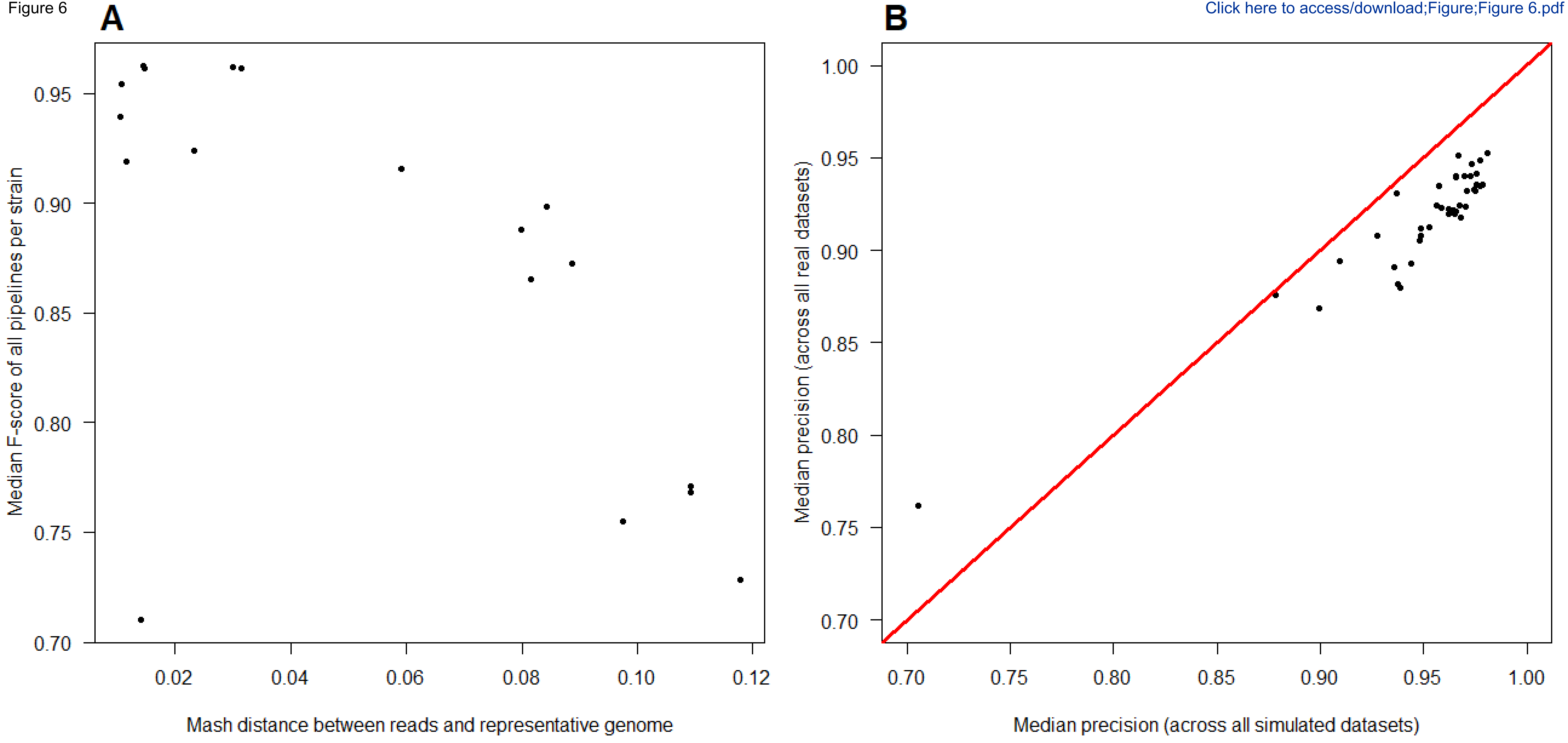

Figure 7

Click here to access/download;Figure;Figure 7.png

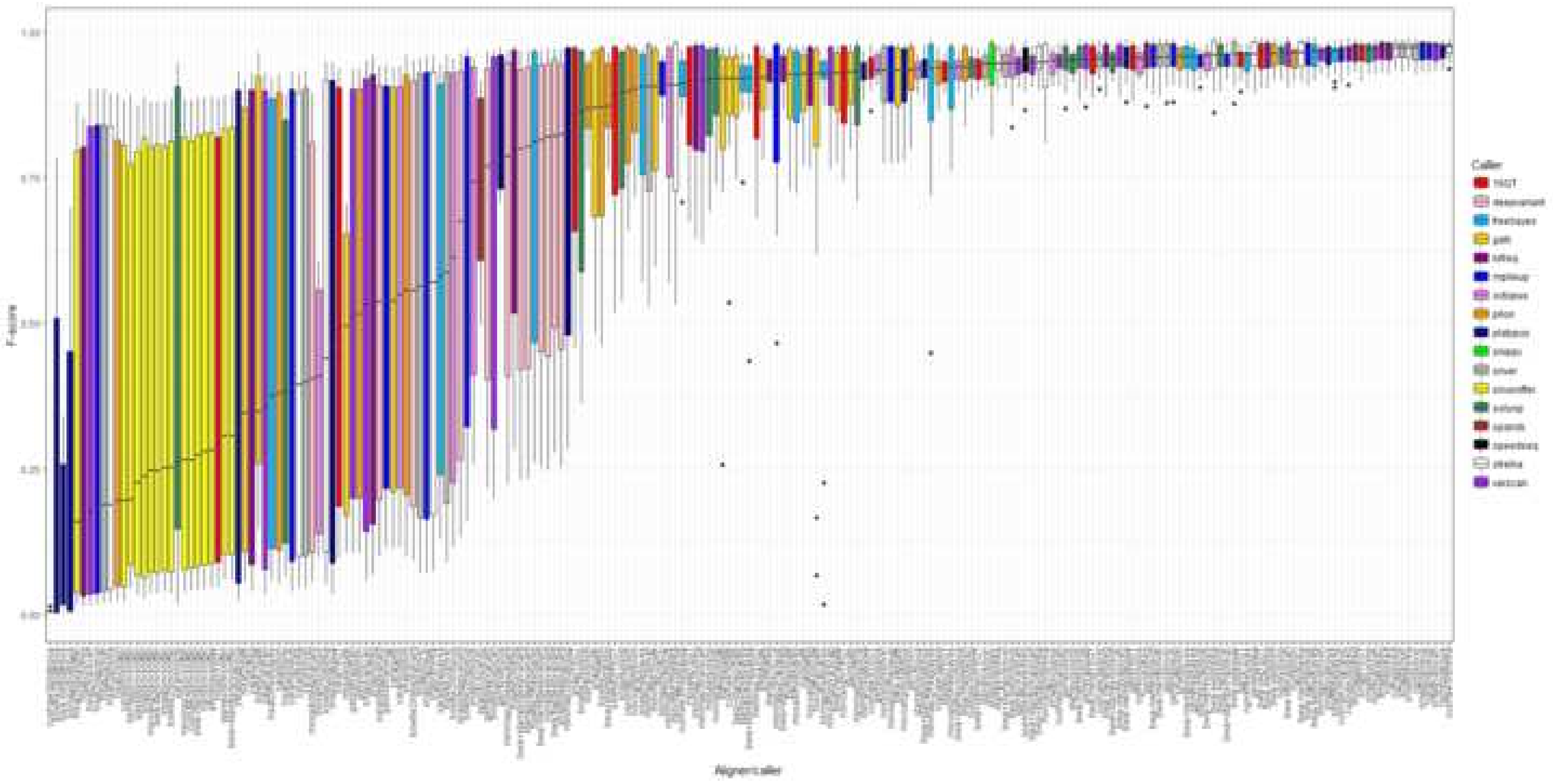

Supplementary Figure 1

Click here to access/download
Supplementary Material
Supplementary Figure 1.png

Supplementary Figure 2

Click here to access/download
Supplementary Material
Supplementary Figure 2.png

Supplementary Figure 3

Click here to access/download
Supplementary Material
Supplementary Figure 3.png

Supplementary Figure 4

Click here to access/download
Supplementary Material
Supplementary Figure 4.png

Supplementary Figure 5

Click here to access/download
Supplementary Material
Supplementary Figure 5.png

Supplementary Figure 6

Click here to access/download
Supplementary Material
Supplementary Figure 6.png

Supplementary Figure 7

Click here to access/download
Supplementary Material
Supplementary Figure 7.png

Supplementary Figure 8

Click here to access/download
Supplementary Material
Supplementary Figure 8.png

Supplementary Figure 9

Click here to access/download
Supplementary Material
Supplementary Figure 9.png

Supplementary Table 1

Click here to access/download
Supplementary Material
Supplementary Table 1.xlsx

Supplementary Table 2

Click here to access/download
Supplementary Material
Supplementary Table 2.xlsx

Supplementary Table 3

Click here to access/download
Supplementary Material
Supplementary Table 3.xlsx

Supplementary Table 4

Click here to access/download
Supplementary Material
Supplementary Table 4.xlsx

Supplementary Table 5

Click here to access/download
Supplementary Material
Supplementary Table 5.xlsx

Supplementary Table 6

Click here to access/download
Supplementary Material
Supplementary Table 6.xlsx

Supplementary Table 7

Click here to access/download
Supplementary Material
Supplementary Table 7.xlsx

Supplementary Table 8

Click here to access/download
Supplementary Material
Supplementary Table 8.xlsx

Supplementary Table 9

Click here to access/download
Supplementary Material
Supplementary Table 9.xlsx

Supplementary Table 10

Click here to access/download
Supplementary Material
Supplementary Table 10.xlsx

Supplementary Table 11

Click here to access/download
Supplementary Material
Supplementary Table 11.xlsx

Supplementary Table 12

Click here to access/download
Supplementary Material
Supplementary Table 12.xlsx

Supplementary Table 13

Click here to access/download
Supplementary Material
Supplementary Table 13.xlsx

Supplementary Table 14

Click here to access/download
Supplementary Material
Supplementary Table 14.xlsx

Supplementary Table 15

Click here to access/download
Supplementary Material
Supplementary Table 15.xlsx

Supplementary Table 16

Click here to access/download
Supplementary Material
Supplementary Table 16.xlsx

Supplementary Table 17

Click here to access/download
Supplementary Material
Supplementary Table 17.xlsx

Supplementary Text 1

Click here to access/download
Supplementary Material
Supplementary Text 1.docx
