# Supplementary material for: Genomic diversity affects the accuracy of bacterial single-nucleotide polymorphism–calling pipelines
Source: Gigascience. 2020 Feb 6;9(2):giaa007. doi: 10.1093/gigascience/giaa007 (PMC7002876; doi:10.1093/gigascience/giaa007)
Supplement: giaa007_Supplement_Files [file giaa007_supplement_files.zip › Supplementary Text 1.docx]

**Supplementary Text**

This supplementary text file contains:

1. the command lines used when running each aligner/caller combination, and
2. operating notes on pipeline functionality,

as well as elaborating on:

(c) the rationale for simulating error-free reads,

(d) the rationale for excluding a local indel realignment step,

(e) the rationale for regularising VCFs,

(f) the rationale for simulating reads at high (50-fold) depth,

(g) the rationale for not repeat-masking the reference genome,

(h) the selection criteria by which programs were excluded from this study, and

(i) the empirical basis of the filter criteria used to parse each VCF.

1. **Command lines**

Command lines for each aligner/caller combination are detailed below. For the purpose of this evaluation, Perl scripts were used to generate each set of command lines, and accordingly, they are presented in this fashion. The colour-coded text refers to variables which may be interpolated. For instance, **$ref** refers to the reference genome (or its appropriate index) used as input to each aligner/caller, and **$fq_1** and **$fq_2** to the paired-end fastq files, respectively.

Output filenames were also given a standard nomenclature, comprising a **$root** component (specifying the genome, read length [150 or 300 bp] and replicate number), followed by the **$aligner**, **$caller** and file type suffix, for example “**$root.$aligner.$caller**.vcf”. Intermediary files may be identified by text immediately preceding the suffix, such as “**$root.$aligner.$caller**.unsorted.bam”.

The exception to the following set of combinatorial aligner/caller command lines are those used for the self-contained pipeline Snippy. Snippy was run using the commands:

snippy --cpus 40 --outdir **$root** --prefix **$root** --cleanup --ref **$ref** --R1 **$fq_1** --R2 **$fq_2**

mv **$root/$root**.filt.vcf **$root.$aligner.$caller**.vcf

rm -r **$root**

The original scripts used for this evaluation are available in Supplementary Dataset 2, hosted online via the Oxford Research Archive at https://ora.ox.ac.uk/objects/uuid:8f902497-955e-4b84-9b85-693ee0e4433e. These Perl scripts output sets of command lines in the form of shell (.sh) scripts.

**First step of each pipeline: command lines for alignment**

All command lines for each of the following aligners produce as output a Picard-cleaned, but unsorted and otherwise not post-processed, BAM: **$root.$aligner**.unsorted.bam. In each case, the variable **$ref** refers to the reference genome (should the aligner use it directly) or its appropriate index. Where programs allowed the number of processors, threads or cores to be specified (e.g. BWA parameter -t), we set the value to 40, commensurate with our available hardware (a high-performance cluster employing the Open Grid Scheduler batch system on Scientific Linux 7).

BBmap

bbmap.sh ref=**$ref** in=**$fq_1** in2=**$fq_2** out=**$root.$aligner**.unsorted.no_read_groups.sam nodisk

samtools view -bS **$root.$aligner**.unsorted.no_read_groups.sam > **$root.$aligner**.unsorted.no_read_groups.bam

rm **$root.$aligner**.unsorted.no_read_groups.sam

java -jar picard.jar CleanSam INPUT=**$root.$aligner**.unsorted.no_read_groups.bam OUTPUT=**$root.$aligner**.unsorted.no_read_groups.cleaned.bam

rm **$root.$aligner**.unsorted.no_read_groups.bam

java -jar picard.jar FixMateInformation INPUT=**$root.$aligner**.unsorted.no_read_groups.cleaned.bam OUTPUT=**$root.$aligner**.unsorted.no_read_groups.cleaned.fixmated.bam

rm **$root.$aligner**.unsorted.no_read_groups.cleaned.bam

java -jar picard.jar AddOrReplaceReadGroups INPUT=**$root.$aligner**.unsorted.no_read_groups.cleaned.fixmated.bam OUTPUT=**$root.$aligner**.unsorted.bam RGID=**$root** RGLB=lib RGPL=Illumina RGPU=unit RGSM=sample

rm **$root.$aligner**.unsorted.no_read_groups.cleaned.fixmated.bam

Bowtie2

bowtie2 --rg-id **$root** --rg SM:sample --rg LIB:lib --rg PL:Illumina -p 40 -x **$ref** -1 **$fq_1** -2 **$fq_2** -S **$root.$aligner**.unsorted.sam

samtools view -bS **$root.$aligner**.unsorted.sam > **$root.$aligner**.unsorted.bam

rm **$root.$aligner**.unsorted.sam

java -jar picard.jar CleanSam INPUT=**$root.$aligner**.unsorted.bam OUTPUT=**$root.$aligner**.unsorted.cleaned.bam

rm **$root.$aligner**.unsorted.bam

mv **$root.$aligner**.unsorted.cleaned.bam **$root.$aligner**.unsorted.bam

BWA-mem

bwa mem -R '@RG\tID:group\tSM:sample\tPL:Illumina\tLIB:lib\tPU:unit' -t 40 -M **$ref $fq_1 $fq_2** | samtools view -Shb - > **$root.$aligner**.unsorted.bam

java -jar picard.jar CleanSam INPUT=**$root.$aligner**.unsorted.bam OUTPUT=**$root.$aligner**.unsorted.cleaned.bam

BWA-sw

cat **$fq_1 $fq_2** > **$concat_fq**

bwa bwasw -t 40 -f **$root.$aligner**.unsorted.no_read_groups.sam **$ref $concat_fq**

rm **$concat_fq**

samtools view -bS **$root.$aligner**.unsorted.no_read_groups.sam > **$root.$aligner**.unsorted.no_read_groups.bam

java -jar picard.jar CleanSam INPUT=**$root.$aligner**.unsorted.no_read_groups.bam OUTPUT=**$root.$aligner**.unsorted.no_read_groups.cleaned.bam

rm **$root.$aligner**.unsorted.no_read_groups.bam

java -jar picard.jar AddOrReplaceReadGroups INPUT=**$root.$aligner**.unsorted.no_read_groups.cleaned.bam OUTPUT=**$root.$aligner**.unsorted.bam RGID=**$root** RGLB=lib RGPL=Illumina RGPU=unit RGSM=sample

rm **$root.$aligner**.unsorted.no_read_groups.cleaned.bam

CUSHAW3

cushaw3 align -rgid **$root** -rgsm sample -rglb lib -rgpl Illumina -t 40 -r **$ref** -q **$fq_1 $fq_2** -o **$root.$aligner**.unsorted.sam

samtools view -bS **$root.$aligner**.unsorted.sam > **$root.$aligner**.unsorted.bam

rm **$root.$aligner**.unsorted.sam

java -jar picard.jar CleanSam INPUT=**$root.$aligner**.unsorted.bam OUTPUT=**$root.$aligner**.unsorted.cleaned.bam

rm **$root.$aligner**.unsorted.bam

mv **$root.$aligner**.unsorted.cleaned.bam **$root.$aligner**.unsorted.bam

GASSST

seqtk seq -A **$fq_1** > **$genome_id**.1.fa

seqtk seq -A **$fq_2** > **$genome_id**.2.fa

cat **$genome_id**.1.fa **$genome_id**.2.fa > **$genome_id**.fa

rm **$genome_id**.1.fa **$genome_id**.2.fa

Gassst -d **$ref** -i **$genome_id**.fa -p 95 -n 40 -o **$root.$aligner**.unsorted.no_read_groups.gassst

gassst_to_sam **$root.$aligner**.unsorted.no_read_groups.gassst **$root.$aligner**.unsorted.no_read_groups.sam

rm **$root.$aligner**.unsorted.no_read_groups.gassst

samtools view -bS **$root.$aligner**.unsorted.no_read_groups.sam > **$root.$aligner**.unsorted.no_read_groups.bam

rm **$root.$aligner**.unsorted.no_read_groups.sam

java -jar picard.jar CleanSam INPUT=**$root.$aligner**.unsorted.no_read_groups.bam OUTPUT=**$root.$aligner**.unsorted.no_read_groups.cleaned.bam

rm **$root.$aligner**.unsorted.no_read_groups.bam

java -jar picard.jar FixMateInformation INPUT=**$root.$aligner**.unsorted.no_read_groups.cleaned.bam OUTPUT=**$root.$aligner**.unsorted.no_read_groups.cleaned.fixmated.bam

rm **$root.$aligner**.unsorted.no_read_groups.cleaned.bam

java -jar picard.jar AddOrReplaceReadGroups INPUT=**$root.$aligner**.unsorted.no_read_groups.cleaned.fixmated.bam OUTPUT=**$root.$aligner**.unsorted.bam RGID=**$root** RGLB=lib RGPL=Illumina RGPU=unit RGSM=sample

rm **$root.$aligner**.unsorted.no_read_groups.cleaned.fixmated.bam

GEM

gem-mapper -I **$ref** -1 **$fq_1** -2 **$fq_2** -r '@RG\tID:group\tSM:sample\tPL:Illumina\tLIB:lib\tPU:unit' -z -p -o **$root.$aligner**.unsorted.sam

samtools view -bS **$root.$aligner**.unsorted.sam > **$root.$aligner**.unsorted.bam

rm **$root.$aligner**.unsorted.sam

java -jar picard.jar CleanSam INPUT=**$root.$aligner**.unsorted.bam OUTPUT=**$root.$aligner**.unsorted.cleaned.bam

rm **$root.$aligner**.unsorted.bam

mv **$root.$aligner**.unsorted.cleaned.bam **$root.$aligner**.unsorted.bam

HISAT2

hisat2-align --rg-id **$root** --rg SM:sample --rg LIB:lib --rg PL:Illumina -p 40 -x **$ref** -1 **$fq_1** -2 **$fq_2** -S **$root.$aligner**.unsorted.sam

samtools view -bS **$root.$aligner**.unsorted.sam > **$root.$aligner**.unsorted.bam

rm **$root.$aligner**.unsorted.sam

java -jar picard.jar CleanSam INPUT=**$root.$aligner**.unsorted.bam OUTPUT=**$root.$aligner**.unsorted.cleaned.bam

rm **$root.$aligner**.unsorted.bam

mv **$root.$aligner**.unsorted.cleaned.bam **$root.$aligner**.unsorted.bam

minimap2

minimap2 -ax sr **$ref** -R '@RG\tID:group\tSM:sample\tPL:Illumina\tLIB:lib\tPU:unit' -t 40 $fq_1 $fq_2 | samtools view -Shb - > **$root.$aligner**.unsorted.bam

java -jar picard.jar CleanSam INPUT=**$root.$aligner**.unsorted.bam OUTPUT=**$root.$aligner**.unsorted.cleaned.bam

rm **$root.$aligner**.unsorted.bam

mv **$root.$aligner**.unsorted.cleaned.bam **$root.$aligner**.unsorted.bam

MOSAIK

./MOSAIK/bin/MosaikBuild -fr **$ref** -oa **$root**.dat

./MOSAIK/bin/MosaikBuild -q **$fq_1** -q2 **$fq_2** -st illumina -out **$root.$aligner**.mkb

./MOSAIK/bin/MosaikAligner -in **$root.$aligner**.mkb -ia **$root**.dat -out **$root.$aligner**.unsorted -p 40 -annpe ./MOSAIK/src/networkFile/2.1.78.pe.ann -annse ./MOSAIK/src/networkFile/2.1.78.se.ann

rm **$root**.dat **$root.$aligner**.mkb **$root.$aligner**.unsorted.stat

java -classpath sam-1.99.jar net.sf.samtools.FixBAMFile **$root.$aligner**.unsorted.bam **$root.$aligner**.unsorted.fixed.bam

mv **$root.$aligner**.unsorted.fixed.bam **$root.$aligner**.unsorted.bam

java -jar picard.jar CleanSam INPUT=**$root.$aligner**.unsorted.bam OUTPUT=**$root.$aligner**.unsorted.cleaned.bam

rm **$root.$aligner**.unsorted.bam

mv **$root.$aligner**.unsorted.cleaned.bam **$root.$aligner**.unsorted.bam

The use of sam-1.99 jar in this set of commands is to avoid a potential downstream error during BAM post-processing. See the ‘operating notes’ section, below, for more details.

NGM

ngm -t 40 -r **$ref** -1 **$fq_1** -2 **$fq_2** --rg-id **$root** --rg-sm sample --rg-lb lib --rg-pl Illumina -o **$root.$aligner**.unsorted.sam

java -jar picard.jar CleanSam INPUT=**$root.$aligner**.unsorted.bam OUTPUT=**$root.$aligner**.unsorted.cleaned.bam

rm **$root.$aligner**.unsorted.bam

mv **$root.$aligner**.unsorted.cleaned.bam **$root.$aligner**.unsorted.bam

Novoalign

novoalign -c 40 -d **$ref** -F STDFQ -o SAM $'@RG\tID:group\tSM:sample\tPL:Illumina\tLIB:lib\tPU:unit' -f **$fq_1 $fq_2** > **$root.$aligner**.unsorted.sam

samtools view -bS **$root.$aligner**.unsorted.sam > **$root.$aligner**.unsorted.bam

rm **$root.$aligner**.unsorted.sam

java -jar picard.jar CleanSam INPUT=**$root.$aligner**.unsorted.bam OUTPUT=**$root.$aligner**.unsorted.cleaned.bam

rm **$root.$aligner**.unsorted.bam

mv **$root.$aligner**.unsorted.cleaned.bam **$root.$aligner**.unsorted.bam

SMALT

smalt map -n 40 -o **$root.$aligner**.unsorted.sam **$ref $fq_1 $fq_2**

samtools view -bS **$root.$aligner**.unsorted.sam > **$root.$aligner**.unsorted.bam

rm **$root.$aligner**.unsorted.sam

java -jar picard.jar CleanSam INPUT=**$root.$aligner**.unsorted.bam OUTPUT=**$root.$aligner**.unsorted.cleaned.bam

rm **$root.$aligner**.unsorted.bam

mv **$root.$aligner**.unsorted.cleaned.bam **$root.$aligner**.unsorted.bam

SNAP

snap-aligner paired **$ref $fq_1 $fq_2** -t 40 -R '@RG\tID:group\tSM:sample\tPL:Illumina\tLIB:lib\tPU:unit' -o **$root.$aligner**.unsorted.sam

samtools view -bS **$root.$aligner**.unsorted.sam > **$root.$aligner**.unsorted.bam

rm **$root.$aligner**.unsorted.sam

java -jar picard.jar CleanSam INPUT=**$root.$aligner**.unsorted.bam OUTPUT=**$root.$aligner**.unsorted.cleaned.bam

rm **$root.$aligner**.unsorted.bam

mv **$root.$aligner**.unsorted.cleaned.bam **$root.$aligner**.unsorted.bam

Stampy (without pre-mapping)

stampy.py -g **$ref** -h **$ref** -t 40 -M **$fq_1 $fq_2** | samtools view -Sb - > **$root.$aligner**.unsorted.no_read_groups.bam

java -jar picard.jar CleanSam INPUT=**$root.$aligner**.unsorted.no_read_groups.bam OUTPUT=**$root.$aligner**.unsorted.no_read_groups.cleaned.bam

rm **$root.$aligner**.unsorted.no_read_groups.bam

java -jar picard.jar AddOrReplaceReadGroups INPUT=**$root.$aligner**.unsorted.no_read_groups.cleaned.bam OUTPUT=**$root.$aligner**.unsorted.bam RGID=**$root** RGLB=lib RGPL=Illumina RGPU=unit RGSM=sample

rm **$root.$aligner**.unsorted.no_read_groups.cleaned.bam

Stampy (with BWA pre-mapping)

bwa aln -t 40 **$ref $fq_1** > **$root.$aligner**.1.sai

bwa aln -t 40 **$ref $fq_2** > **$root.$aligner**.2.sai

bwa sampe **$ref $root.$aligner**.1.sai **$root.$aligner**.2.sai **$fq_1 $fq_2** | samtools view -Sb - > **$root.$aligner**.unsorted.pre_stampy.bam

stampy.py -g **$ref** -h **$ref** -t 40 --bamkeepgoodreads -M **$root.$aligner**.unsorted.pre_stampy.bam | samtools view -Sb - > **$root.$aligner**.unsorted.no_read_groups.bam

rm **$root.$aligner**.1.sai **$root.$aligner**.2.sai **$root.$aligner**.unsorted.pre_stampy.bam

java -jar picard.jar CleanSam INPUT=**$root.$aligner**.unsorted.no_read_groups.bam OUTPUT=**$root.$aligner**.unsorted.no_read_groups.cleaned.bam

rm **$root.$aligner**.unsorted.no_read_groups.bam

java -jar picard.jar AddOrReplaceReadGroups INPUT=**$root.$aligner**.unsorted.no_read_groups.cleaned.bam OUTPUT=**$root.$aligner**.unsorted.bam RGID=**$root** RGLB=lib RGPL=Illumina RGPU=unit RGSM=sample

rm **$root.$aligner**.unsorted.no_read_groups.cleaned.bam

Yara

yara_mapper **$ref $fq_1 $fq_2** --threads 40 -rg '@RG\tID:group\tSM:sample\tPL:Illumina\tLIB:lib\tPU:unit' -output-file **$root.$aligner**.unsorted.uncleaned.bam

java -jar picard.jar CleanSam INPUT=**$root.$aligner**.unsorted.uncleaned.bam OUTPUT=**$root.$aligner**.unsorted.bam

rm **$root.$aligner**.unsorted.uncleaned.bam

**Second step of each pipeline: command lines for post-processing BAMs**

These command lines constitute an intermediary step in each pipeline, taking as input an unsorted BAM, **$root.$aligner**.unsorted.bam, and producing as output a sorted, de-duplicated and indexed BAM, **$root.$aligner**.bam. This is the final output of the alignment process and used as input to the third step of each pipeline: variant calling. All intermediary BAMs are discarded.

java -jar picard.jar SortSam INPUT=**$root.$aligner**.unsorted.bam OUTPUT=**$root.$aligner**.sorted.bam SORT_ORDER=coordinate

java -jar picard.jar MarkDuplicates INPUT=**$root.$aligner**.sorted.bam OUTPUT=**$root.$aligner**.bam METRICS_FILE=**$root.$aligner**.metrics ASSUME_SORTED=true

java -jar picard.jar BuildBamIndex INPUT=**$root.$aligner**.bam

rm **$root.$aligner**.unsorted.bam **$root.$aligner**.sorted.bam **$root.$aligner**.metrics

**Final step of each pipeline: command lines for variant calling**

All command lines for each of the following callers produce as output an unregularized VCF: **$root.$aligner**.**$caller**.vcf. This file is then regularised using the vcfallelicprimitives module of VCFlib, producing a final VCF for evaluation:

vcfallelicprimitives **$root.$aligner.$caller**.vcf > **$root.$aligner.$caller**.regularised.vcf.

16GT

bam2snapshot -i **$ref** -b **$root.$aligner**.bam -o **$root.$aligner.$caller**

snapshotSnpcaller -i **$ref** -o **$root.$aligner.$caller**

perl txt2vcf.pl **$root.$aligner.$caller**.txt **$root.$aligner.$caller $ref** > **$root.$aligner.$caller**.vcf

rm **$root.$aligner.$caller**.alignmentQC.txt **$root.$aligner.$caller**.snapshot **$root.$aligner.$caller**.txt

DeepVariant

DeepVariant was used via Docker [1]; this approach is detailed at https://github.com/google/deepvariant/blob/master/docs/deepvariant-quick-start.md. Prior to doing so, Docker-mountable input and output directories were created. Into the former was copied the reference genome and its SAMtools index (**$ref** and **$ref**.fai, respectively), alongside the indexed BAM.

mkdir ./deepvariant_input

mkdir ./deepvariant_output

cp **$ref** ./deepvariant_input

cp **$ref**.fai ./deepvariant_input

cp **$root.$aligner**.bam ./deepvariant_input

cp **$root.$aligner**.bai ./deepvariant_input

docker run -v ./deepvariant_input:/input -v ./deepvariant_output:/output gcr.io/deepvariant-docker/deepvariant:0.8.0 /opt/deepvariant/bin/run_deepvariant --model_type=WGS --ref=/input/**$ref** --reads=/input/**$root.$aligner**.bam --output_vcf=/output/**$root.$aligner.$caller**.vcf --num_shards=40

cp ./deepvariant_output/**$root.$aligner.$caller**.vcf ./**$root.$aligner.$caller**.vcf

rm -r ./deepvariant_output

rm -r ./deepvariant_input

Freebayes

freebayes -f **$ref** --ploidy 1 **$root.$aligner**.bam > **$root.$aligner.$caller**.vcf

GATK

gatk HaplotypeCaller -R **$ref** -I **$root.$aligner**.bam -O **$root.$aligner.$caller**.vcf

rm **$root.$aligner.$caller**.vcf.idx

LoFreq

lofreq call -f **$ref** -o **$root.$aligner.$caller**.vcf **$root.$aligner**.bam

mpileup

bcftools mpileup -Ou -f **$ref $root.$aligner**.bam | bcftools call --threads 40 --ploidy 1 -mv -Ov -o **$root.$aligner.$caller**.vcf

Octopus

octopus --reference **$ref** --reads **$root.$aligner**.bam --legacy --threads 40 > **$root.$aligner.$caller**.vcf

Pilon

java -jar pilon-1.23.jar --genome **$ref** --bam **$root.$aligner**.bam --threads 40 --outdir **$root.$aligner.$caller** --output **$root.$aligner.$caller** --vcf

mv **$root.$aligner.$caller/$root.$aligner.$caller**.vcf **$root.$aligner.$caller**.vcf

rm -r **$root.$aligner.$caller**

Platypus

python Python.py callVariants --bamFiles=**$root.$aligner**.bam --logFileName=**$root.$aligner.$caller**.log --refFile=**$ref** --output=**$root.$aligner.$caller**.vcf

rm **$root.$aligner.$caller**.log

SNVer

java -jar SNVerIndividual.jar -i **$root.$aligner**.bam -r **$ref** -o **$root.$aligner.$caller**

mv **$root.$aligner.$caller**.filter.vcf **$root.$aligner.$caller**.vcf

rm **$root.$aligner.$caller**.failed.log **$root.$aligner.$caller**.indel.filter.vcf **$root.$aligner.$caller**.indel.raw.vcf **$root.$aligner.$caller**.filter.vcf **$root.$aligner.$caller**.raw.vcf

SNVSniffer

samtools view -H **$root.$aligner**.bam > **$root.$aligner**.header.sam

SNVSniffer snp -f 2 -g **$ref** -o **$root.$aligner.$caller**.vcf **$root.$aligner**.header.sam **$root.$aligner**.bam

rm **$root.$aligner**.header.sam

SolSNP

java -jar SolSNP.jar INPUT=**$root.$aligner**.bam OUTPUT=**$root.$aligner.$caller**.vcf R=**$ref** OUTPUT_FORMAT=VCF

Strelka

python configureStrelkaGermlineWorkflow.py --bam **$root.$aligner**.bam --referenceFasta **$ref** --runDir **$root.$aligner.$caller**

python **$root.$aligner.$caller**/runWorkflow.py -m local -j 40

gunzip **$root.$aligner.$caller**/results/variants/genome.S1.vcf.gz

mv **$root.$aligner.$caller**/results/variants/genome.S1.vcf **$root.$aligner.$caller**.vcf

rm -r **$root.$aligner.$caller**

VarScan

samtools mpileup -B -q 1 -f **$ref** **$root.$aligner**.bam > **$root.$aligner.$caller**.mpileup

java -jar VarScan.v2.3.9.jar mpileup2snp **$root.$aligner.$caller**.mpileup --output-vcf 1 > **$root.$aligner.$caller**.vcf

rm **$root.$aligner.$caller**.mpileup

1. **Operating notes**

The following section constitutes miscellaneous notes on our experience with particular tools and may help both in replicating these findings and providing additional contextual information.

- When using NGM for alignment, we chose to output SAM instead of taking the program’s -b option to output BAM. This was to avoid a downstream error when validating index bins during BAM post-processing (detailed at https://gatkforums.broadinstitute.org/gatk/discussion/4290/sam-bin-field-error-for-the-gatk-run): “bin field of BAM record does not equal value computed based on alignment start and end, and length of sequence to which read is aligned.” As SAM files do not have a field for indexing bin, no error would arise if outputting in this format.
- The HISAT/Platypus combination was not used in this study because in numerous instances HISAT BAMs could not be successfully parsed by Platypus, resulting in a (fatal) segmentation fault: “Exception OverflowError: 'value too large to convert to short' in 'htslibWrapper.ReadIterator.get' ignored”. This error was due to CIGAR strings with H operations longer than the maximum value of ‘short’, and had previously been documented at https://groups.google.com/forum/#!topic/platypus-users/xHp_ZwhyxuM.
- Should a BAM contain no valid alignments, we found that SNVer, although running to completion without error, would produce only an empty VCF as output, **$root.$aligner.$caller**.raw.vcf, not the filtered one, **$root.$aligner.$caller**.filter.vcf.
- After running MOSAIK the subsidiary program sam-1.99.jar was necessary to resolve a downstream error in BAM indexing bins when running Picard MarkDuplicates: “bin field of BAM record does not equal value computed based on alignment start and end, and length of sequence to which read is aligned”. This error has been documented at https://sourceforge.net/p/samtools/mailman/message/31853465/ and https://gatkforums.broadinstitute.org/gatk/discussion/4290/sam-bin-field-error-for-the-gatk-run. sam-1.99.jar, used to correct MOSAIK BAMs, is available via Picard Tools but not as part of its standard deployment: https://downloads.sourceforge.net/project/picard/sam-jdk/1.99/sam-1.99.jar. An alternative solution to this error is to set the VALIDATION_STRINGENCY parameter of Picard Tools to ‘LENIENT’ (the default value is ‘STRICT’).
- Snippy produces two VCFs, **$root/$root**.raw.vcf and **$root/$root**.filt.vcf, the filtered and unfiltered output of Freebayes, respectively. For the purpose of this evaluation, we only retained the latter as the default recommendation although, as with all other pipelines, post-processed this VCF with the COMPASS-derived filter criteria detailed in Supplementary Table 12. However, irrespective of whether **$root**.raw.vcf or **$root**.filt.vcf was used, the Snippy pipeline would have already applied its own internal set of filter criteria on the basis of, for BAM parsing, minimum read mapping quality (default: Phred score ≥ 60) and minimum base quality (default: Phred score ≥ 13), and, for VCF parsing, minimum site depth (default: ≥ 10 reads) and minimum variant quality (default: Phred score ≥ 100). These internal criteria are more stringent than the COMPASS-derived minimum site depth and variant quality thresholds applied in this study for VCF filtering. Accordingly, we found no additional benefit to post-processing in this case.
- We found that SolSNP VCFs could not be regularised by vcfallelicprimitives as a result of the error “Invalid character '.' in 'GQ' FORMAT field”. In each SolSNP VCF, the value in question is a floating-point number but the FORMAT field defined it, incorrectly, as an integer. For the purpose of this evaluation, we edited this line in each SolSNP VCF, changing the word ‘Integer’ to ‘Float’. vcfallelicprimitives also reported as a warning the message “INFO 'AR' is not defined in the header, assuming Type=String”, although this did not affect functionality.

1. **Comparing pipeline performance when simulating both error-free and error-containing reads**

When initially evaluating each pipeline, all reads were simulated error-free. This was in order to exclude biases at other points in the workflow, such as in DNA library preparation. To compare pipeline performance between sets of error-free and error-containing reads, a parallel set of equivalent simulations – of 3 sets of 150bp and 3 sets of 300bp paired-end reads, each at 50x base-level coverage and aligned both to the same genome from which they were simulated and to a divergent genome – were performed only for the set of *E. coli* strains (*E. coli* was chosen as it was among the most diverse of the 10 species in this study, with the greatest range of genome sizes; see Supplementary Table 5).

Error-containing reads were simulated using dwgsim v0.1.11 (https://github.com/nh13/DWGSIM) with parameters -e 0.001-0.01 (non-uniform per-base error rate increasing across the read from 0.01 to 0.1%, approximating a generic Illumina error profile) and -y 0.01 (1% probability of simulating a random DNA read). Parallel sets of error-free reads were simulated with dwgsim parameters -e 0-0 and -y 0.

dwgsim does not output the otherwise randomly generated seed used for each simulation, although does allow seeds to be provided. To ensure results were reproducible, the same seeds were provided to dwgsim as were generated during the initial set of error-free (wgsim) simulations (i.e., as given in Supplementary Table 3).

This dataset contains 4 sets of 7134 VCFs, 2 made using dwgsim error-free reads (i.e., aligned to the same genome and aligned to the representative genome) and 2 made using error-containing reads. Each dataset of 7134 VCFs comprises 2 read lengths (150 and 300bp) * 3 replicates * 29 *E. coli* strains * 41 pipelines. All VCFs were filtered according to criteria detailed in Supplementary Table 12 (and which were originally empirically selected for use with the COMPASS pipeline; see below).

The performance statistics for each pipeline are shown in Supplementary Tables 13 (for reads aligned to the same genome from which they were simulated) and 14 (for reads aligned to the representative genome).

When aligning reads to the same genome from which they were simulated, there were near-perfect correlations between F-scores obtained using error-free and error-containing reads (Spearman’s *rho* = 0.9981, p < 10^-15^; see figure below). Sequencing error does introduce a (negligible) number of false positive calls, however. The correlation between estimates of precision (positive predictive value) is, while strong, marginally weaker than the correlation between estimates of recall (sensitivity): for precision, Spearman’s *rho* = 0.9282 (p < 10^-15^) and for recall, Spearman’s *rho* = 0.9979 (p < 10^-15^). Similar results are seen when aligning reads to a divergent, representative, genome (see figure below), with equivalently strong correlations between F-score (Spearman’s *rho* = 0.9889, p < 10^-15^), precision (Spearman’s *rho* = 0.9870, p < 10^-15^) and recall (Spearman’s *rho* = 0.9862, p < 10^-15^).

These results suggest that introducing sequencing error into the simulated datasets has a negligible effect on the overall performance rank of each pipeline, even in the absence of pre-processing quality control (such as by, e.g., the read trimmer Trimmomatic [2]) which should in principle diminish error further.

This can likely be attributed to the VCF filter criteria (which are broadly similar to those recommended by a previous study for maximising SNP validation rate [3]) and/or because many of the aligners already apply internal mechanisms for accommodating error (such as ‘soft-clipping’: omitting unaligned, i.e. error-prone, terminal regions from reads and using only partial alignments between the reads and reference [4]).


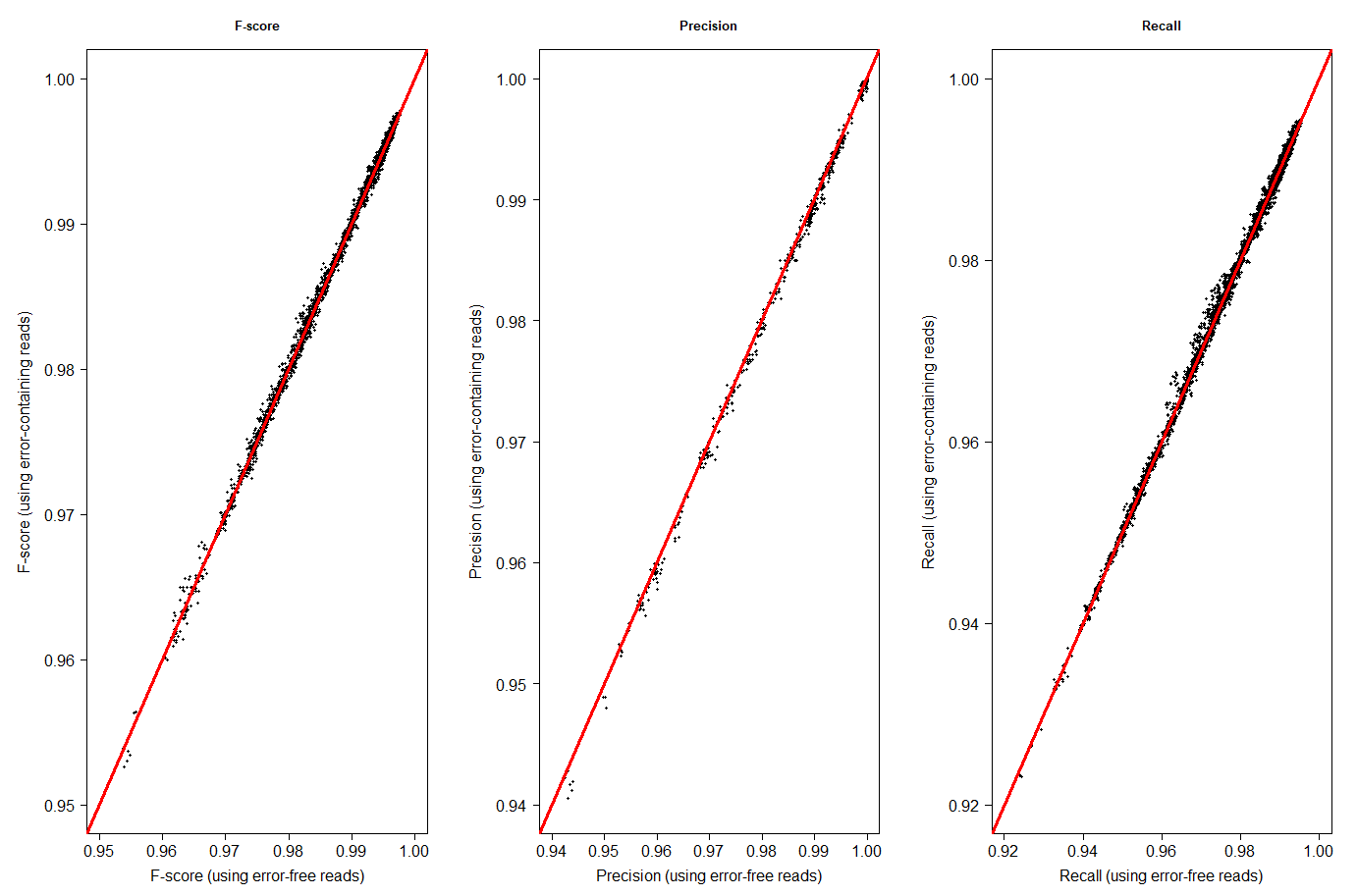


**Head-to-head performance comparison of pipelines evaluated using both error-free and error-containing reads, aligning reads to the same genome from which they were simulated.**

This figure directly compares the performance of pipelines using both error-free and error-containing reads (which have a non-uniform per-base error rate increasing across the read from 0.01 to 0.1%). In both cases, reads are simulated from 29 *E. coli* strains (detailed in Supplementary Table 2) and aligned back to the same genome. Each point represents a simulation (n = 7134, i.e. 2 read lengths [150 and 300bp] * 3 replicates * 29 *E. coli* strains * 41 pipelines). Summary statistics for each simulation are shown in Supplementary Table 13. The line y = x is shown in red.


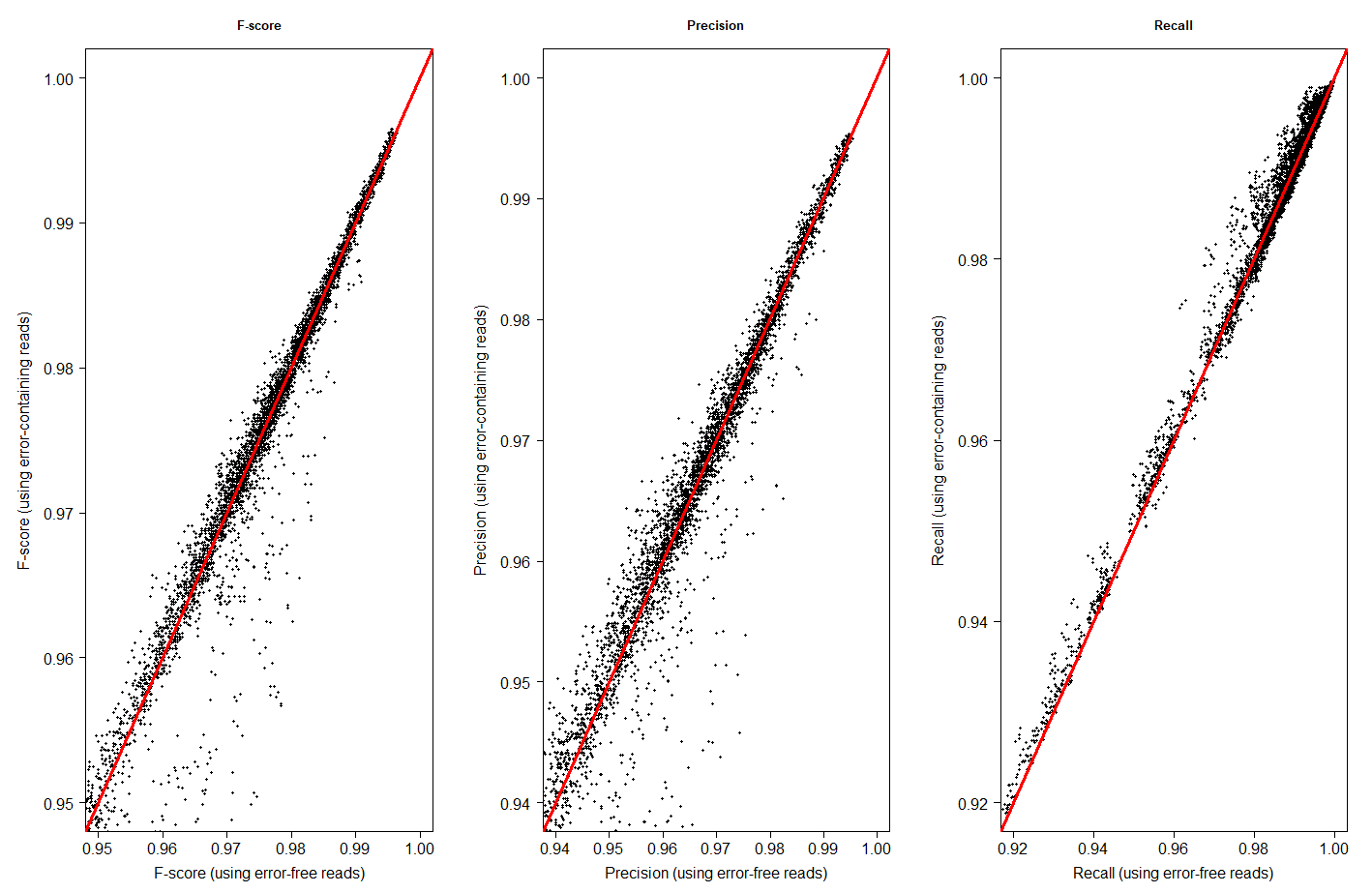


**Head-to-head performance comparison of pipelines evaluated using both error-free and error-containing reads, aligning reads to a different genome from which they were simulated.**

This figure directly compares the performance of pipelines using both error-free and error-containing reads (which have a non-uniform per-base error rate increasing across the read from 0.01 to 0.1%). In both cases, reads are simulated from 29 *E. coli* strains (detailed in Supplementary Table 2) and aligned back to a different, representative, genome. Each point represents a simulation (n = 7134, i.e. 2 read lengths [150 and 300bp] * 3 replicates * 29 *E. coli* strains * 41 pipelines). Summary statistics for each simulation are shown in Supplementary Table 14. The line y = x is shown in red.

1. **Comparing pipeline performance with error-free reads if adding an additional BAM post-processing step of local indel realignment**

A formerly commonplace post-processing step for many aligner/caller pipelines was to locally realign around indels in the BAM, correcting for mismatching bases that could otherwise be mistaken for SNPs (these can be introduced by indels in the genome that are absent in the reference).

However, indel realignment, using the GATK modules RealignerTargetCreator and IndelRealigner, is not necessarily required for variant discovery if using a variant caller that already incorporates a haplotype assembly step, either by local *de novo* re-assembly (such as Platypus, Strelka or GATK HaplotypeCaller, the successor to UnifiedGenotyper) or by building the haplotype directly from the reads (such as Freebayes). A previous study has also demonstrated that local indel realignment, even in divergent regions, had minimal impact on SNP calling accuracy [5]. To that end, the RealignerTargetCreator and IndelRealigner modules were removed from GATK v4 (and in any case are unable to re-align reads around insertions > 30bp), although this does not preclude potential added-value benefit if applied to poor-quality data (https://software.broadinstitute.org/gatk/blog?id=7847, accessed 2nd April 2019).

Consequently, we did not incorporate a routine local realignment step in each pipeline (between Picard MarkDuplicates and BuildBamIndex; see ‘second step of each pipeline’, above). To test whether local indel realignment had a quantifiable effect upon pipeline performance, we created a parallel set of simulations – of 3 sets of error-free 150bp and 3 sets of error-free 300bp paired-end reads, each at 50x base-level coverage and aligned to a divergent genome – for the diverse set of *E. coli* strains (as above). These can be directly contrasted with the error-free *E. coli* simulations in Supplementary Table 6.

To perform indel realignment, we used a deprecated version of GATK, v3.8.1 (https://github.com/broadgsa/gatk/releases, accessed 30^th^ May 2019), the last available with this functionality.

The performance statistics for each pipeline, both with- and without local indel realignment, are shown in Supplementary Table 15. There were near-perfect correlations between F-scores irrespective of whether local indel realignment was performed (Spearman’s *rho* = 0.9993, p < 10^-15^; see figure below). Equivalently strong correlations were found between precision (Spearman’s *rho* = 0.9987, p < 10^-15^) and recall (Spearman’s *rho* = 0.9993, p < 10^-15^). While local indel realignment reduces false positive calls, so increasing precision, among the poorer-performing pipelines, the effect size of this difference is negligible (Cliff’s delta = -0.008).


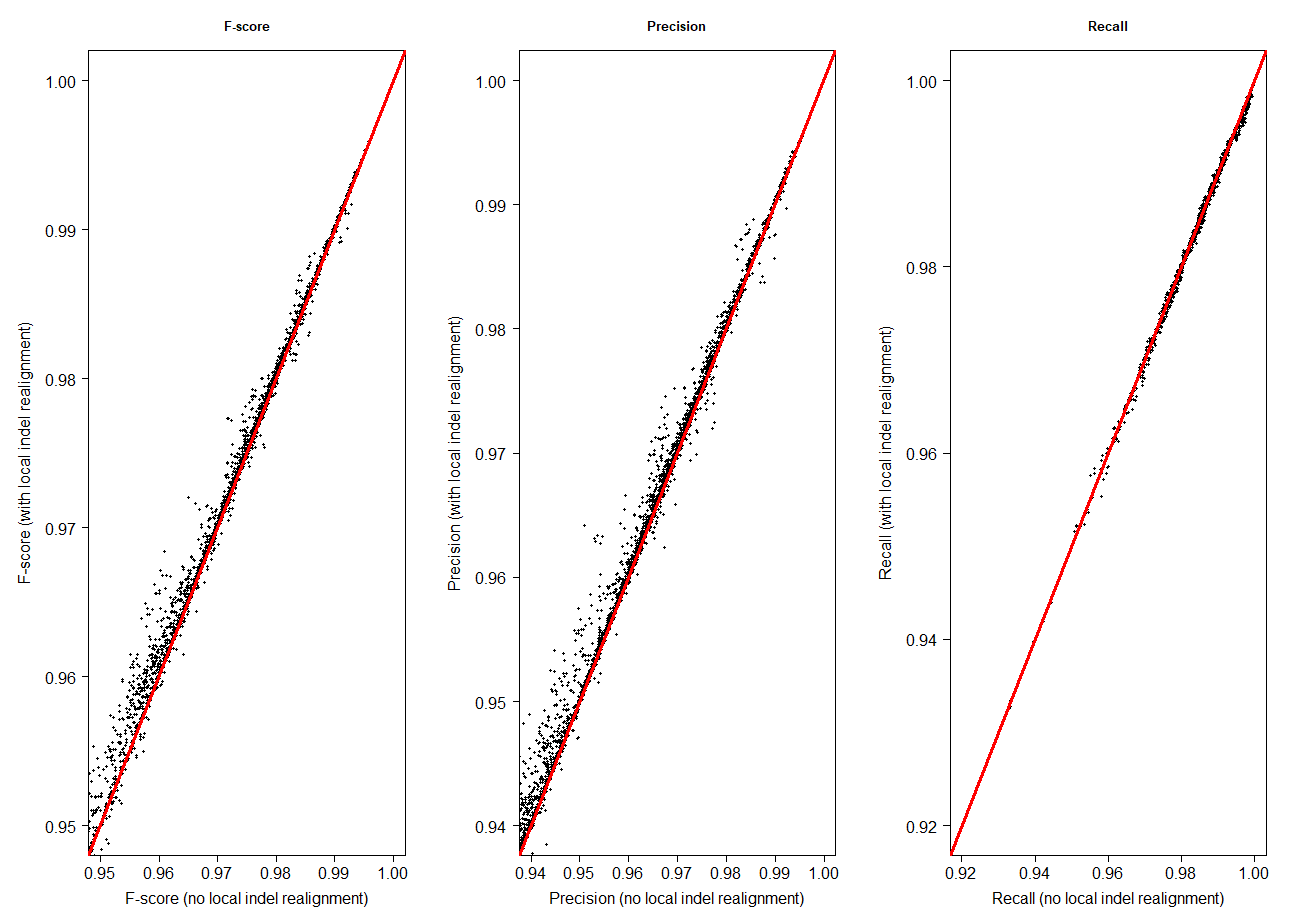


**Head-to-head performance comparison of pipelines evaluated both with and without a local indel realignment step, aligning reads to a different genome from which they were simulated.**

This figure directly compares the performance of pipelines evaluated both with and without a BAM post-processing step of local indel realignment. In both cases, reads are simulated from 29 *E. coli* strains (detailed in Supplementary Table 2) and aligned back to a different, representative, genome. Each point represents a simulation (n = 7134, i.e. 2 read lengths [150 and 300bp] * 3 replicates * 29 *E. coli* strains * 41 pipelines). Summary statistics for each simulation are shown in Supplementary Table 15. The line y = x is shown in red.

1. **Comparing pipeline performance with error-free reads if omitting the VCF post-processing step of regularisation**

Different variant callers output VCFs that can represent the same indel or complex variant in different ways. For the purpose of this evaluation, we post-processed all VCFs using the vcfallelicprimitives module of vcflib v1.0.0-rc2 (https://github.com/ekg/vcflib). In the absence of VCF regularisation (which would split adjacent SNPs into individual SNPs and otherwise standardise variant calls), we considered that VCFs could not be meaningfully contrasted – when compared to the same truth set of SNPs, two different representations of the same SNP would be evaluated differently. To demonstrate that VCF regularisation had a quantifiable effect upon pipeline performance, we created (as described above) a parallel set of simulations – of 3 sets of error-free 150bp and 3 sets of error-free 300bp paired-end reads, each at 50x base-level coverage and aligned to a divergent genome – for the diverse set of *E. coli* strains. These can be directly contrasted with the error-free *E. coli* simulations in Supplementary Table 6, as illustrated in the figure below. The raw data for this figure – performance statistics for each pipeline, both with- and without VCF regularisation – are shown in Supplementary Table 16. While VCFs output by many callers produce near-identical values irrespective of VCF regularisation (that is, they have a common approach to representing SNPs, resulting in multiple points along the line *y* = *x*) there are several exceptions, notably 16GT, LoFreq and Snippy, for which estimates of precision and recall are both distorted if evaluating only the non-regularised VCFs. It is important to note that this distortion is only relative to the ‘truth set’ of positions in this study, which have a set representation. Consequently, any deviation from *y* = *x* is not due to the accuracy of each tool but rather, how it chooses to report calls – at least, relative to the way in which the ‘truth set’ is defined for this study, accepting that no one representation is intrinsically superior.

Finally, note that vcfallelicprimitives, in parsing ‘blocks’ of variants, acts in a specific manner: “if multiple allelic primitives (gaps or mismatches) are specified in a single VCF record, split the record into multiple lines, but drop all INFO fields” (https://github.com/vcflib/vcflib#vcfallelicprimitives). In practice, this means that if an entire ‘block’ passed an (internal) filter applied by the variant caller, the FILTER column for the whole block would be set to PASS, with this status then carried through to each of the individual records (this applies also to failing positions). However, various other details normally specified in the INFO column (for example, DP) would not be carried forward into the individual records – but this is because many of these details would not be valid on an individual basis. Values in the INFO field for the original record would be aggregate properties for that ‘block’ and could not be re-calculated for individual positions. Regularisation with vcfallelicprimitives, therefore, produces a VCF with more detail available for some positions than others.

This would only be of note if applying any extra filters after regularisation (for instance, to each position on the basis of read depth) – these filters could not then be applied as the relevant information has been removed from the INFO field. However, it is important to note that these filters (as used in this study and detailed in Supplementary Table 12) could not be legitimately applied to those positions in the non-regularised VCF anyway (in the above example, for individual positions in a long block, DP would vary over its length) – and so the removal of values from the INFO field would not affect downstream VCF parsing.


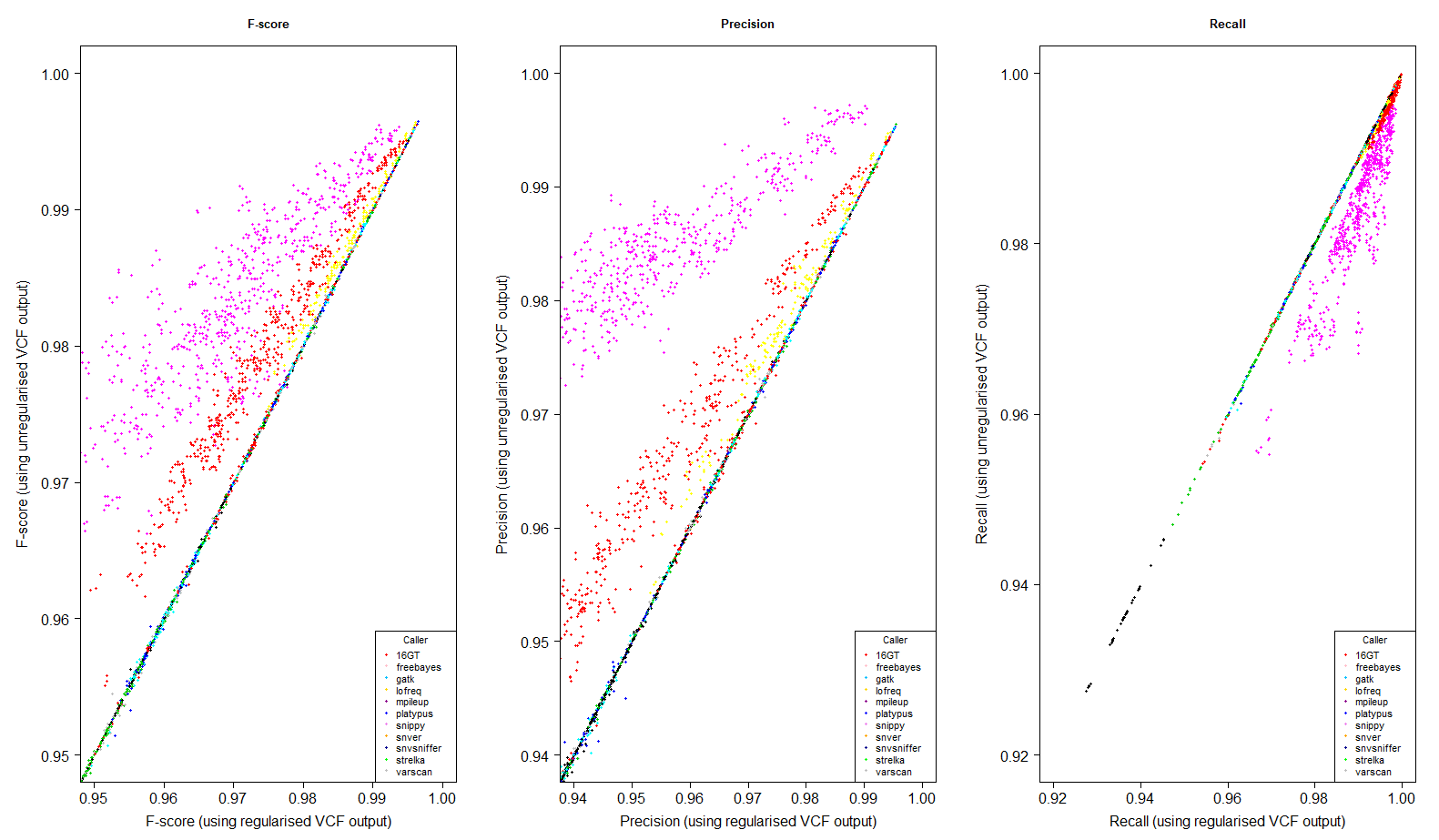


**Head-to-head performance comparison of pipelines evaluated both with and without VCF regularisation, aligning reads to a different genome from which they were simulated.**

This figure directly compares the performance of pipelines evaluated both with and without a final post-processing step of VCF regularisation. In both cases, reads are simulated from 29 *E. coli* strains (detailed in Supplementary Table 2) and aligned back to a different, representative, genome. Each point represents a simulation (n = 7134, i.e. 2 read lengths [150 and 300bp] * 3 replicates * 29 *E. coli* strains * 41 pipelines), coloured by variant caller. Summary statistics for each simulation are shown in Supplementary Table 16.

1. **Comparing pipeline performance with error-free reads at varying read depths**

The simulated datasets used in this study were all created at a high depth of coverage (a mean base-level depth of 50-fold). We assumed that given the relatively cheap cost of sequencing and the small genome sizes of bacteria, a reasonable expectation for real data would be a high depth of coverage and that, consistent with a previous study, variant calling sensitivity would be largely unaffected by increases in coverage [6]. To evaluate this assumption, we created a parallel set of simulations – of 3 sets of error-free 150bp and 3 sets of error-free 300bp paired-end reads, each aligned to a divergent genome – using the same diverse set of *E. coli* strains detailed above. We simulated reads at 5-, 10- and 25-fold mean base-level coverage, i.e. (DEPTH x genome length)/read length, where DEPTH is 5, 10 or 25, respectively, using in each case the same wgsim seed given in Supplementary Table 6. These results can be directly contrasted with the error-free 50-fold *E. coli* simulations in Supplementary Table 6. Comparisons of lower (5, 10, or 25-fold) with higher (50-fold) depths are illustrated in the figure below.

Relative to VCFs generated at 50-fold coverage, we found that progressively lower depths of coverage had poorer recall (there were a greater number of false negative calls) but higher precision (there was a reduced likelihood of false positive calls). The net effect was to increase noise in the F-score distribution at lower coverage depths. However, the figure can also be interpreted to suggest that above a certain threshold coverage there would likely be negligible difference between VCFs generated at two different read depths: variance in the 25-fold to 50-fold comparison is, for instance, far lower than in the 10-fold to 50-fold comparison.

Finally, we found that although high precision could still be attained at the lowest depth of coverage (5-fold), recall in this case was greatly compromised. This was because the VCF filter criteria used in this study (detailed in Supplementary Table 12) required a minimum of 5 reads at each variant position, which could not be met in many cases.


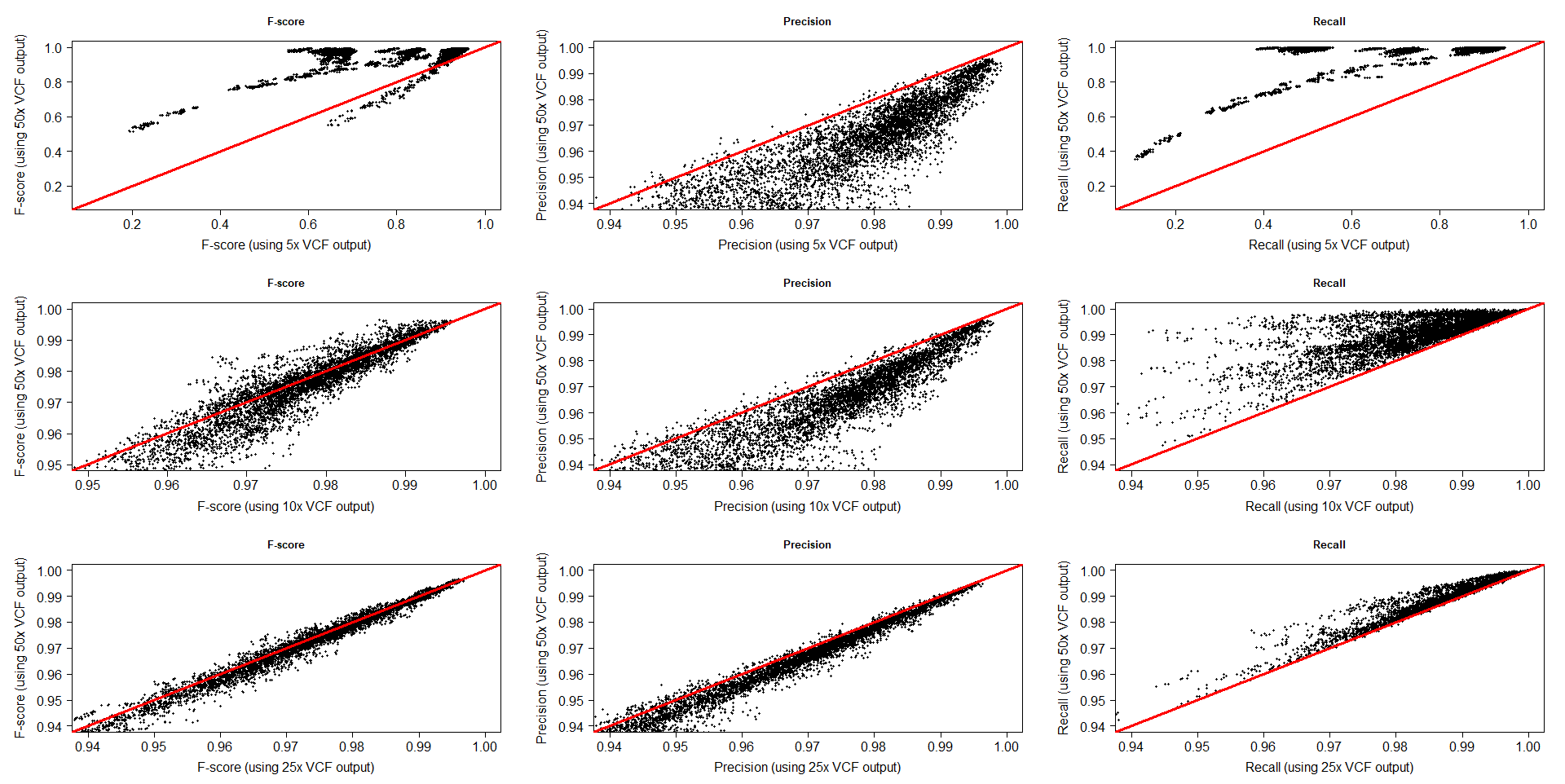


**Head-to-head performance comparison of pipelines at different read depths, aligning reads to a different genome from which they were simulated.**

This figure directly compares the performance of pipelines evaluated using simulated reads at a high depth of coverage (mean base-level coverage of 50-fold) with three lower depth simulations (mean base level coverage of 5-, 10-, and 25-fold). In each case, reads were simulated from 29 *E. coli* strains (detailed in Supplementary Table 2) and aligned back to a different, representative, genome. Each point represents a simulation (n = 7134, i.e. 2 read lengths [150 and 300bp] * 3 replicates * 29 *E. coli* strains * 41 pipelines). Summary statistics for each simulation are shown in Supplementary Table 17. The line y = x is shown in red.

1. **Comparing pipeline performance when using a repeat-masked reference genome**

Paralogous, or internally repetitive, regions within a reference genome are likely to increase the rate of read mis-mapping and thereby the number of false positive calls made by each pipeline. However, for the purpose of this evaluation, we did not seek to identify and exclude SNPs from these regions as, even if present, this would have a systematic negative effect on the performance of each pipeline.

For the strain-to-representative genome alignments used in this evaluation, we considered as the ‘truth set’ of SNPs those calls made only within one-to-one alignment blocks and so cannot exclude the possibility that repetitive or highly mutable regions within these blocks have been misaligned. However, as we also required each base within these one-to-one alignment blocks to be supported by both nucmer and ParSnp calls, this implicitly masks ambiguous bases. For this reason we did not consider it necessary to also repeat-mask each reference genome prior to alignment.

To demonstrate the effect of additional repeat-masking, we re-calculated precision, recall and F-score for each of the 209 pipelines evaluated using real sequencing data (i.e., when aligning 18 sets of non-simulated reads against one of the six representative Gram-negative genomes detailed in Supplementary Table 8). We did not test the effect of repeat-masking using the simulated *E. coli* datasets (as above) because this represents only one reference genome (i.e., *E. coli* K-12 substr. MG1655). Repetitive regions in each genome were first identified by self-self BLASTn (as in [78]), using BLAST+ v2.7.1 with default parameters, and considered those with alignments of ≥ 95% identity over length ≥ 100bp, with no more than 1 gap, and an E-value < 0.05 (not including the match of the entire genome against itself).

The representative genomes used to align the non-simulated sequencing data, and their associated BLASTn reports, are available in Supplementary Dataset 2 (hosted online via the Oxford Research Archive at https://ora.ox.ac.uk/objects/uuid:8f902497-955e-4b84-9b85-693ee0e4433e). Performance statistics for each pipeline, with and without additional repeat-masking, are given in Supplementary Table 9 and illustrated below. This figure demonstrates that the effect of additional repeat-masking on overall F-score is negligible, although it does result in a marginal improvement to precision. Nevertheless, the effect appears systematic, with the majority of points in the figure below, but parallel, to the line y = x.

It is also important to note that the parameters used for repeat-masking will determine which paralogues will be successfully masked. For the purpose of this study, we used reasonably conservative parameters and so expect to have primarily masked only the more similar paralogues. The likelihood of mis-mapping (and thereby false positive SNP calling) would increase among more divergent paralogues, although optimising parameters to detect these is non-trivial. More lenient repeat-masking parameters, in masking more divergent positions, would also reduce the number of true SNPs it is possible to call.


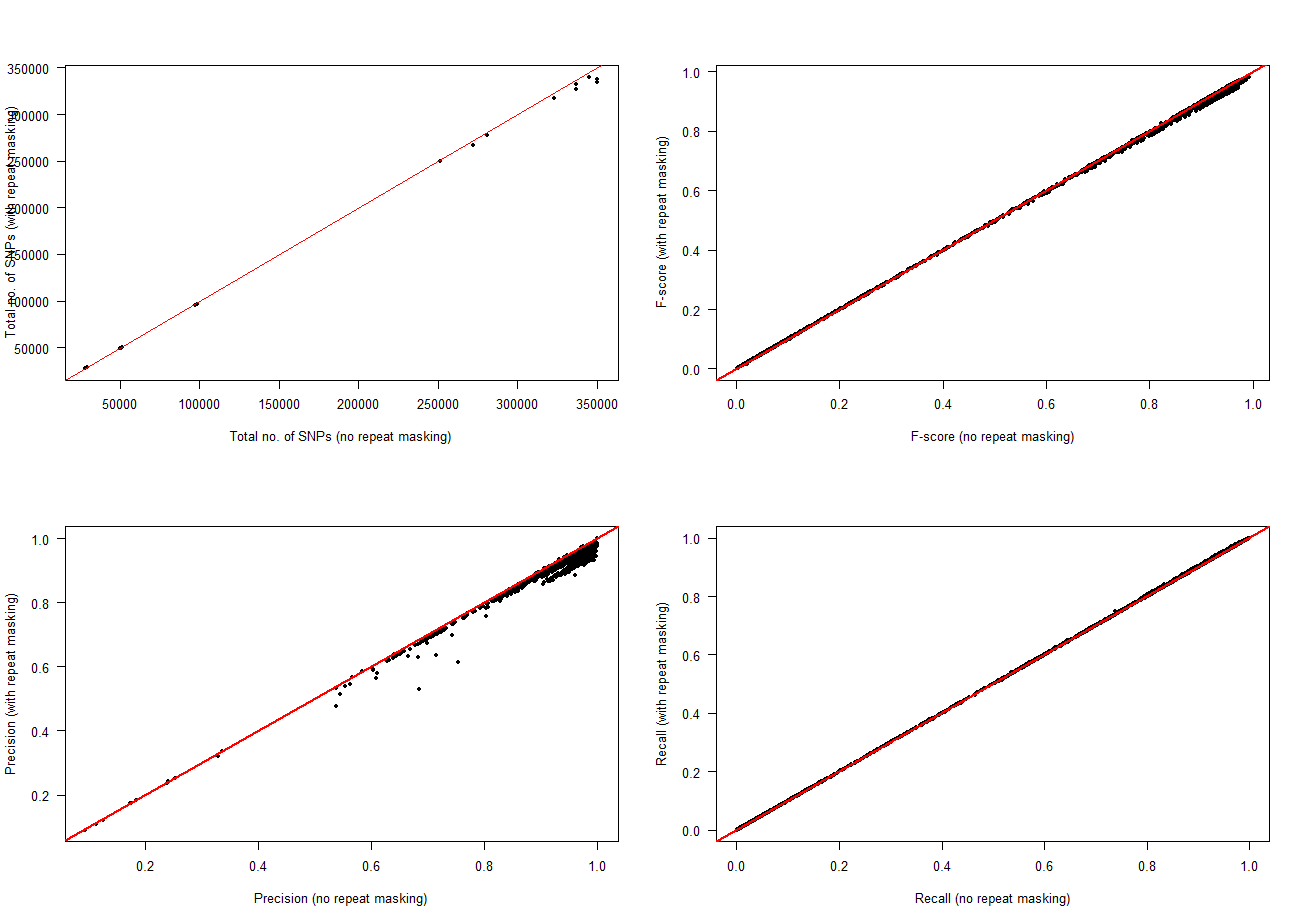


**Head-to-head performance comparison of pipelines evaluated using a repeat-masked or non-masked reference genome, aligning reads to a different genome from which they were simulated.**

This figure directly compares the performance of 209 pipelines evaluated using real sequencing reads from 18 Gram-negative genomes (detailed in Supplementary Table 8) aligned against both a repeat-masked or non-masked reference genome. Note that in this context ‘repeat-masking’ supplements the implicit masking of ambiguous bases required in both cases (i.e. requiring all reference bases to be supported by consensus nucmer and ParSnp calls in one-to-one alignment blocks; see Materials and Methods). Additional repeat masking marginally reduces the number of SNPs available to call in each genome, particularly for more divergent genomes (i.e. those with a greater number of SNPs), although the effect on overall F-score is negligible. Each point in the F-score, precision and recall plots represents a simulation (n = 3762, i.e. 18 genomes * 209 pipelines). Summary statistics for each simulation are shown in Supplementary Table 9. The line y = x is shown in red.

1. **Programs not evaluated in this study**

As there are comparatively few variant calling evaluations using bacterial data, we sought to test a broad range of programs, specifically those that are in common use, under active development and which produce comparable output in standard formats (BAM, VCF).

To create a shortlist of candidate aligners and variant callers, we used similar criteria to a previous study [6], excluding from consideration programs that:

(a) were formally deprecated (such as the aligners SHRiMP2 [7] and Masai [8] [discouraged in favour of its successor, Yara], and callers GATK UnifiedGenotyper [discouraged in favour of its successor, GATK HaplotypeCaller] and Isaac [9] [discouraged in favour of its successor, Strelka [10]]),

(b) were not being actively developed (having no major version update in many years),

(c) were not intended for use with Illumina reads (such as drFAST [11] and lordFAST [12], which are intended only for use with AB SOLiD colour space and PacBio reads, respectively),

(d) were only intended for use with single-molecule reads (such as Clairvoyante [13]),

(e) were only capable of calling specific types of variant (such as the indel-only Dindel [14], SNP-only SNPSVM [15] and structural variant-only BreakDancer [16]),

(f) had hardware requirements which could not be met (such as SOAP3-dp [17], which required a CUDA-enabled GPU),

(g) required paired, non-identical, samples as input (a common requirement of many somatic variant callers [18], such as Virmid [19] and JointSNVMix [20]),

(h) required familial relationships between samples as input (a requirement of *de novo* mutation callers such as DeNovoGear [21], FamSeq [22], and Polymutt [23]),

(f) made assumptions of the input which could not be met by the simulated datasets (such as DiscoSnp [24] and DiscoSnp++ [25], which are intended to call only isolated SNPs, i.e. those distant both up- and downstream from any other polymorphism by at least *k* nucleotides),

(g) were explicitly discouraged for use on diverse samples (such as SKA [26], which is optimised for highly-related genomes), or

(h) did not produce output in BAM/VCF format (such as BactSNP [27], which outputs SNP calls in a TSV file, and Breseq [28], which outputs SNP calls in ‘GenomeDiff’ format).

We also excluded ‘all-in-one’ tools that allowed the user a choice of components, such as PHEnix (https://github.com/phe-bioinformatics/PHEnix), which requires that the user select one of the aligners BWA or Bowtie2 and one of the callers GATK or mpileup. The performance of such a tool cannot easily be evaluated in one test.

Overall, we aimed for this evaluation to be extensive but it is neither realistic nor computationally tractable to be exhaustive. For practicality, we excluded numerous short read aligners that would otherwise meet the above criteria, including but not limited to GSNAP [29], Hobbes3 [30], mrFAST [31], mrsFAST [32], mrsFAST-Ultra [33], RazerS [34], RazerS3 [35], SeqAlto [36], SRmapper [37], and ZOOM [38].

Although it is not possible to benchmark all (or even a majority) of tools on all possible metrics, one of the core findings of this evaluation is that the Mash distance between the reads and the reference genome is especially critical to pipeline performance. To that end, there is no compelling reason to believe that those tools which have not been evaluated – assuming they were not designed for this scenario – will differ greatly in performance from those that have.

1. **Overview of the COMPASS (Complete Pathogen Sequencing Solution) pipeline and associated VCF parsing criteria**

The filter criteria used to parse each VCF (detailed in Supplementary Table 12) were adapted from those empirically developed for use in the COMPASS pipeline. COMPASS development began in 2010 by the Modernising Medical Microbiology (MMM) team (http://modmedmicro.nsms.ox.ac.uk/), with the aim to process a high volume of sequencing data into a form suitable for clinical reports. The rationale behind the development of COMPASS was that although a variety of tools were available for individual sequence analyses (for reviews, see [39, 40]), an end-to-end software solution, for use in routine clinical practice, was not. COMPASS was developed for this purpose on a cloud-based platform to offer the capability of future growth.

While not independently published, results generated by COMPASS have been utilised in numerous publications, demonstrating the strength and applicability of the pipeline [41-49]. COMPASS comprises several processes that download raw sequencing data for a given sample, undertake quality control to ensure the sequencing has worked, assembles a reference genome, maps reads to this reference, and then calls variants from the mapped read data. To do so, COMPASS employs BLAST+ v2.2.23 [50], Bowtie2 v2.2.23 [51], BWA v0.7.5a [52], FastQC v0.11.2, GATK v1.4.21 [53], Kraken v0.10.06 [54], Mykrobe Predictor v0.3.1-0-g87 [55], Picard Tools v1.31, SAMtools v0.1.19 [56], and Stampy v1.0.23 [57], with bespoke linking scripts written in Python v2.7.1 and using standard Python libraries as required.

Using per-site statistics from the subsequent VCF and empirically-determined filter criteria, COMPASS then re-calls each base in the assembled sample with respect to the reference genome, outputting an expanded VCF that contains calls for every site. As such, by extracting the ‘alt’ column of this VCF, a FASTA file can be obtained for each sample. COMPASS attempts base calling only for sample sites that have homologues in the reference genome, so the expanded VCF (and subsequent FASTA) always contains the same number of sites as the reference genome. Should one of the COMPASS filter criteria not be met, an ‘N’ call is made instead, indicating insufficient, inconclusive or conflicting information for that site.

The set of filter criteria employed by COMPASS underwent several revisions, of which only the final set of criteria for the current version are used in this evaluation. Consistency in variant calling between different sets of criteria was assessed by resequencing isolates from the same bacterial colonies on different flow cells as technical replicates. The criteria used for evaluation were the number of discordant calls between each pair of replicates, which measured how often a variant was incorrectly detected between identical genomes (i.e. false positive rate), and the proportion of the genome called between a pair of replicate sequences. This indirectly measured the “false negative” rate – assuming no-calls occur randomly, the call rate would be proportional to how often a true variant was not detected between a pair of genomes.

Briefly, COMPASS requires all variant calls to be homozygous under a diploid model. In all versions of COMPASS, variant calls were made only in non-repetitive regions of the core genome, with repetitive regions identified by self-self BLASTn and subsequently masked. For the purpose of this evaluation, we did not exclude calls within these regions as this would require appending VCFs prior to filtering, i.e. filtering calls on the basis of information (a ‘within a repetitive region’ flag) not initially output by any aligner/caller combination. In addition, the retention of repetitive regions, and their associated lower-confidence calls, would have a systematic negative effect on the performance of each pipeline.

The initial set of COMPASS filter criteria (as used in, for instance, [58]) required that a SNP have a consensus of 75% of the mapped reads and support from at least five reads, including one in each direction, at a site with a depth of high-quality (mapping quality Phred score > 20) coverage between the 2.5 and 97.5 percentiles of all sites for that isolate (i.e. the SNP does not occur at a site with unusual depth). Sites where minority variants represented more than 10% of read depth were also defined as ‘mixed’, with no base called. Variant calls were also not made if they occurred within 12bp of another nucleotide variant or indel. The most recent set of COMPASS filters, used in this evaluation, removed the ‘unusual depth’ and ‘nearby variant’ criteria as their absence was found to increase mean genome coverage without affecting estimates of genetic relatedness or introducing false positive calls [59]. The minimum requirements for coverage, Phred quality score, and call consensus were retained.

**References**

1. Merkel D: **Docker: lightweight Linux containers for consistent development and deployment.** *Linux J* 2014, **2014:**2.

2. Bolger AM, Lohse M, Usadel B: **Trimmomatic: a flexible trimmer for Illumina sequence data.** *Bioinformatics (Oxford, England)* 2014, **30:**2114-2120.

3. Jia P, Li F, Xia J, Chen H, Ji H, Pao W, Zhao Z: **Consensus rules in variant detection from next-generation sequencing data.** *PLoS ONE* 2012, **7:**e38470-e38470.

4. Tian S, Yan H, Neuhauser C, Slager SL: **An analytical workflow for accurate variant discovery in highly divergent regions.** *BMC Genomics* 2016, **17:**703.

5. Tian S, Yan H, Kalmbach M, Slager SL: **Impact of post-alignment processing in variant discovery from whole exome data.** *BMC bioinformatics* 2016, **17:**403-403.

6. Sandmann S, de Graaf AO, Karimi M, van der Reijden BA, Hellström-Lindberg E, Jansen JH, Dugas M: **Evaluating Variant Calling Tools for Non-Matched Next-Generation Sequencing Data.** *Scientific Reports* 2017, **7:**43169.

7. David M, Dzamba M, Lister D, Ilie L, Brudno M: **SHRiMP2: sensitive yet practical SHort Read Mapping.** *Bioinformatics* 2011, **27:**1011-1012.

8. Siragusa E, Weese D, Reinert K: **Fast and accurate read mapping with approximate seeds and multiple backtracking.** *Nucleic Acids Res* 2013, **41:**e78.

9. Raczy C, Petrovski R, Saunders CT, Chorny I, Kruglyak S, Margulies EH, Chuang HY, Kallberg M, Kumar SA, Liao A, et al: **Isaac: ultra-fast whole-genome secondary analysis on Illumina sequencing platforms.** *Bioinformatics* 2013, **29:**2041-2043.

10. Saunders CT, Wong WS, Swamy S, Becq J, Murray LJ, Cheetham RK: **Strelka: accurate somatic small-variant calling from sequenced tumor-normal sample pairs.** *Bioinformatics* 2012, **28:**1811-1817.

11. Hormozdiari F, Hach F, Sahinalp SC, Eichler EE, Alkan C: **Sensitive and fast mapping of di-base encoded reads.** *Bioinformatics (Oxford, England)* 2011, **27:**1915-1921.

12. Haghshenas E, Sahinalp SC, Hach F: **lordFAST: sensitive and Fast Alignment Search Tool for LOng noisy Read sequencing Data.** *Bioinformatics* 2019, **35:**20-27.

13. Luo R, Sedlazeck FJ, Lam T-W, Schatz MC: **A multi-task convolutional deep neural network for variant calling in single molecule sequencing.** *Nature Communications* 2019, **10:**998.

14. Albers CA, Lunter G, MacArthur DG, McVean G, Ouwehand WH, Durbin R: **Dindel: accurate indel calls from short-read data.** *Genome Res* 2011, **21:**961-973.

15. O'Fallon BD, Wooderchak-Donahue W, Crockett DK: **A support vector machine for identification of single-nucleotide polymorphisms from next-generation sequencing data.** *Bioinformatics* 2013, **29:**1361-1366.

16. Chen K, Wallis JW, McLellan MD, Larson DE, Kalicki JM, Pohl CS, McGrath SD, Wendl MC, Zhang Q, Locke DP, et al: **BreakDancer: an algorithm for high-resolution mapping of genomic structural variation.** *Nat Methods* 2009, **6:**677-681.

17. Luo R, Wong T, Zhu J, Liu CM, Zhu X, Wu E, Lee LK, Lin H, Zhu W, Cheung DW, et al: **SOAP3-dp: fast, accurate and sensitive GPU-based short read aligner.** *PLoS One* 2013, **8:**e65632.

18. Teer JK, Zhang Y, Chen L, Welsh EA, Cress WD, Eschrich SA, Berglund AE: **Evaluating somatic tumor mutation detection without matched normal samples.** *Human Genomics* 2017, **11:**22.

19. Kim S, Jeong K, Bhutani K, Lee JH, Patel A, Scott E, Nam H, Lee H, Gleeson JG, Bafna V: **Virmid: accurate detection of somatic mutations with sample impurity inference.** *Genome Biology* 2013, **14:**R90.

20. Roth A, Ding J, Morin R, Crisan A, Ha G, Giuliany R, Bashashati A, Hirst M, Turashvili G, Oloumi A, et al: **JointSNVMix: a probabilistic model for accurate detection of somatic mutations in normal/tumour paired next-generation sequencing data.** *Bioinformatics* 2012, **28:**907-913.

21. Ramu A, Noordam MJ, Schwartz RS, Wuster A, Hurles ME, Cartwright RA, Conrad DF: **DeNovoGear: de novo indel and point mutation discovery and phasing.** *Nature Methods* 2013, **10:**985.

22. Peng G, Fan Y, Wang W: **FamSeq: A Variant Calling Program for Family-Based Sequencing Data Using Graphics Processing Units.** *PLOS Computational Biology* 2014, **10:**e1003880.

23. Li B, Chen W, Zhan X, Busonero F, Sanna S, Sidore C, Cucca F, Kang HM, Abecasis GR: **A Likelihood-Based Framework for Variant Calling and De Novo Mutation Detection in Families.** *PLOS Genetics* 2012, **8:**e1002944.

24. Uricaru R, Rizk G, Lacroix V, Quillery E, Plantard O, Chikhi R, Lemaitre C, Peterlongo P: **Reference-free detection of isolated SNPs.** *Nucleic Acids Research* 2015, **43:**e11-e11.

25. Peterlongo P, Riou C, Drezen E, Lemaitre C: **DiscoSnp++: de novo detection of small variants from raw unassembled read set(s).** *bioRxiv* 2017**:**209965.

26. Harris SR: **SKA: Split Kmer Analysis Toolkit for Bacterial Genomic Epidemiology.** *bioRxiv* 2018**:**453142.

27. Yoshimura D, Kajitani R, Gotoh Y, Katahira K, Okuno M, Ogura Y, Hayashi T, Itoh T: **Evaluation of SNP calling methods for closely related bacterial isolates and a novel high-accuracy pipeline: BactSNP.** *Microbial Genomics* 2019, **5**.

28. Deatherage DE, Barrick JE: **Identification of mutations in laboratory-evolved microbes from next-generation sequencing data using breseq.** *Methods in molecular biology (Clifton, NJ)* 2014, **1151:**165-188.

29. Wu TD, Nacu S: **Fast and SNP-tolerant detection of complex variants and splicing in short reads.** *Bioinformatics (Oxford, England)* 2010, **26:**873-881.

30. Kim J, Li C, Xie X: **Improving read mapping using additional prefix grams.** *BMC Bioinformatics* 2014, **15:**42.

31. Alkan C, Kidd JM, Marques-Bonet T, Aksay G, Antonacci F, Hormozdiari F, Kitzman JO, Baker C, Malig M, Mutlu O, et al: **Personalized copy number and segmental duplication maps using next-generation sequencing.** *Nature genetics* 2009, **41:**1061-1067.

32. Hach F, Hormozdiari F, Alkan C, Hormozdiari F, Birol I, Eichler EE, Sahinalp SC: **mrsFAST: a cache-oblivious algorithm for short-read mapping.** *Nature methods* 2010, **7:**576-577.

33. Hach F, Sarrafi I, Hormozdiari F, Alkan C, Eichler EE, Sahinalp SC: **mrsFAST-Ultra: a compact, SNP-aware mapper for high performance sequencing applications.** *Nucleic Acids Res* 2014, **42:**W494-500.

34. Weese D, Emde A-K, Rausch T, Döring A, Reinert K: **RazerS--fast read mapping with sensitivity control.** *Genome research* 2009, **19:**1646-1654.

35. Weese D, Holtgrewe M, Reinert K: **RazerS 3: faster, fully sensitive read mapping.** *Bioinformatics* 2012, **28:**2592-2599.

36. Mu JC, Jiang H, Kiani A, Mohiyuddin M, Bani Asadi N, Wong WH: **Fast and accurate read alignment for resequencing.** *Bioinformatics* 2012, **28:**2366-2373.

37. Gontarz PM, Berger J, Wong CF: **SRmapper: a fast and sensitive genome-hashing alignment tool.** *Bioinformatics* 2013, **29:**316-321.

38. Lin H, Zhang Z, Zhang MQ, Ma B, Li M: **ZOOM! Zillions of oligos mapped.** *Bioinformatics (Oxford, England)* 2008, **24:**2431-2437.

39. Wyres KL, Conway TC, Garg S, Queiroz C, Reumann M, Holt K, Rusu LI: **WGS Analysis and Interpretation in Clinical and Public Health Microbiology Laboratories: What Are the Requirements and How Do Existing Tools Compare?** *Pathogens* 2014, **3:**437-458.

40. Land M, Hauser L, Jun SR, Nookaew I, Leuze MR, Ahn TH, Karpinets T, Lund O, Kora G, Wassenaar T, et al: **Insights from 20 years of bacterial genome sequencing.** *Funct Integr Genomics* 2015, **15:**141-161.

41. Eyre DW, Babakhani F, Griffiths D, Seddon J, Del Ojo Elias C, Gorbach SL, Peto TE, Crook DW, Walker AS: **Whole-genome sequencing demonstrates that fidaxomicin is superior to vancomycin for preventing reinfection and relapse of infection with Clostridium difficile.** *J Infect Dis* 2014, **209:**1446-1451.

42. Young BC, Golubchik T, Batty EM, Fung R, Larner-Svensson H, Votintseva AA, Miller RR, Godwin H, Knox K, Everitt RG, et al: **Evolutionary dynamics of Staphylococcus aureus during progression from carriage to disease.** *Proc Natl Acad Sci U S A* 2012, **109:**4550-4555.

43. Walker TM, Crook DW, Peto TE, Conlon CP: **Whole-genome sequencing identifies nosocomial transmission of extra-pulmonary M. tuberculosis.** *QJM* 2016, **109:**819-820.

44. Walker TM, Ip CL, Harrell RH, Evans JT, Kapatai G, Dedicoat MJ, Eyre DW, Wilson DJ, Hawkey PM, Crook DW, et al: **Whole-genome sequencing to delineate Mycobacterium tuberculosis outbreaks: a retrospective observational study.** *Lancet Infect Dis* 2013, **13:**137-146.

45. Walker TM, Kohl TA, Omar SV, Hedge J, Del Ojo Elias C, Bradley P, Iqbal Z, Feuerriegel S, Niehaus KE, Wilson DJ, et al: **Whole-genome sequencing for prediction of Mycobacterium tuberculosis drug susceptibility and resistance: a retrospective cohort study.** *Lancet Infect Dis* 2015, **15:**1193-1202.

46. Price JR, Golubchik T, Cole K, Wilson DJ, Crook DW, Thwaites GE, Bowden R, Walker AS, Peto TE, Paul J, Llewelyn MJ: **Whole-genome sequencing shows that patient-to-patient transmission rarely accounts for acquisition of Staphylococcus aureus in an intensive care unit.** *Clin Infect Dis* 2014, **58:**609-618.

47. Stoesser N, Giess A, Batty EM, Sheppard AE, Walker AS, Wilson DJ, Didelot X, Bashir A, Sebra R, Kasarskis A, et al: **Genome sequencing of an extended series of NDM-producing Klebsiella pneumoniae isolates from neonatal infections in a Nepali hospital characterizes the extent of community- versus hospital-associated transmission in an endemic setting.** *Antimicrob Agents Chemother* 2014, **58:**7347-7357.

48. Stoesser N, Batty EM, Eyre DW, Morgan M, Wyllie DH, Del Ojo Elias C, Johnson JR, Walker AS, Peto TE, Crook DW: **Predicting antimicrobial susceptibilities for Escherichia coli and Klebsiella pneumoniae isolates using whole genomic sequence data.** *J Antimicrob Chemother* 2013, **68:**2234-2244.

49. Gordon NC, Price JR, Cole K, Everitt R, Morgan M, Finney J, Kearns AM, Pichon B, Young B, Wilson DJ, et al: **Prediction of Staphylococcus aureus antimicrobial resistance by whole-genome sequencing.** *J Clin Microbiol* 2014, **52:**1182-1191.

50. Camacho C, Coulouris G, Avagyan V, Ma N, Papadopoulos J, Bealer K, Madden TL: **BLAST+: architecture and applications.** *BMC Bioinformatics* 2009, **10:**421.

51. Langmead B, Salzberg SL: **Fast gapped-read alignment with Bowtie 2.** *Nat Methods* 2012, **9:**357-359.

52. Li H, Durbin R: **Fast and accurate short read alignment with Burrows-Wheeler transform.** *Bioinformatics* 2009, **25:**1754-1760.

53. McKenna A, Hanna M, Banks E, Sivachenko A, Cibulskis K, Kernytsky A, Garimella K, Altshuler D, Gabriel S, Daly M, DePristo MA: **The Genome Analysis Toolkit: a MapReduce framework for analyzing next-generation DNA sequencing data.** *Genome Res* 2010, **20:**1297-1303.

54. Wood DE, Salzberg SL: **Kraken: ultrafast metagenomic sequence classification using exact alignments.** *Genome Biol* 2014, **15:**R46.

55. Bradley P, Gordon NC, Walker TM, Dunn L, Heys S, Huang B, Earle S, Pankhurst LJ, Anson L, de Cesare M, et al: **Rapid antibiotic-resistance predictions from genome sequence data for Staphylococcus aureus and Mycobacterium tuberculosis.** *Nat Commun* 2015, **6:**10063.

56. Li H, Handsaker B, Wysoker A, Fennell T, Ruan J, Homer N, Marth G, Abecasis G, Durbin R, Genome Project Data Processing S: **The Sequence Alignment/Map format and SAMtools.** *Bioinformatics* 2009, **25:**2078-2079.

57. Lunter G, Goodson M: **Stampy: a statistical algorithm for sensitive and fast mapping of Illumina sequence reads.** *Genome Res* 2011, **21:**936-939.

58. Walker TM, Ip CLC, Harrell RH, Evans JT, Kapatai G, Dedicoat MJ, Eyre DW, Wilson DJ, Hawkey PM, Crook DW, et al: **Whole-genome sequencing to delineate Mycobacterium tuberculosis outbreaks: a retrospective observational study.** *The Lancet Infectious diseases* 2013, **13:**137-146.

59. Walker TM, Lalor MK, Broda A, Ortega LS, Morgan M, Parker L, Churchill S, Bennett K, Golubchik T, Giess AP, et al: **Assessment of Mycobacterium tuberculosis transmission in Oxfordshire, UK, 2007-12, with whole pathogen genome sequences: an observational study.** *The Lancet Respiratory medicine* 2014, **2:**285-292.
