# Supplementary figures and images for: Genomic diversity affects the accuracy of bacterial single-nucleotide polymorphism–calling pipelines
Source: Gigascience. 2020 Feb 6;9(2):giaa007. doi: 10.1093/gigascience/giaa007 (PMC7002876; doi:10.1093/gigascience/giaa007)

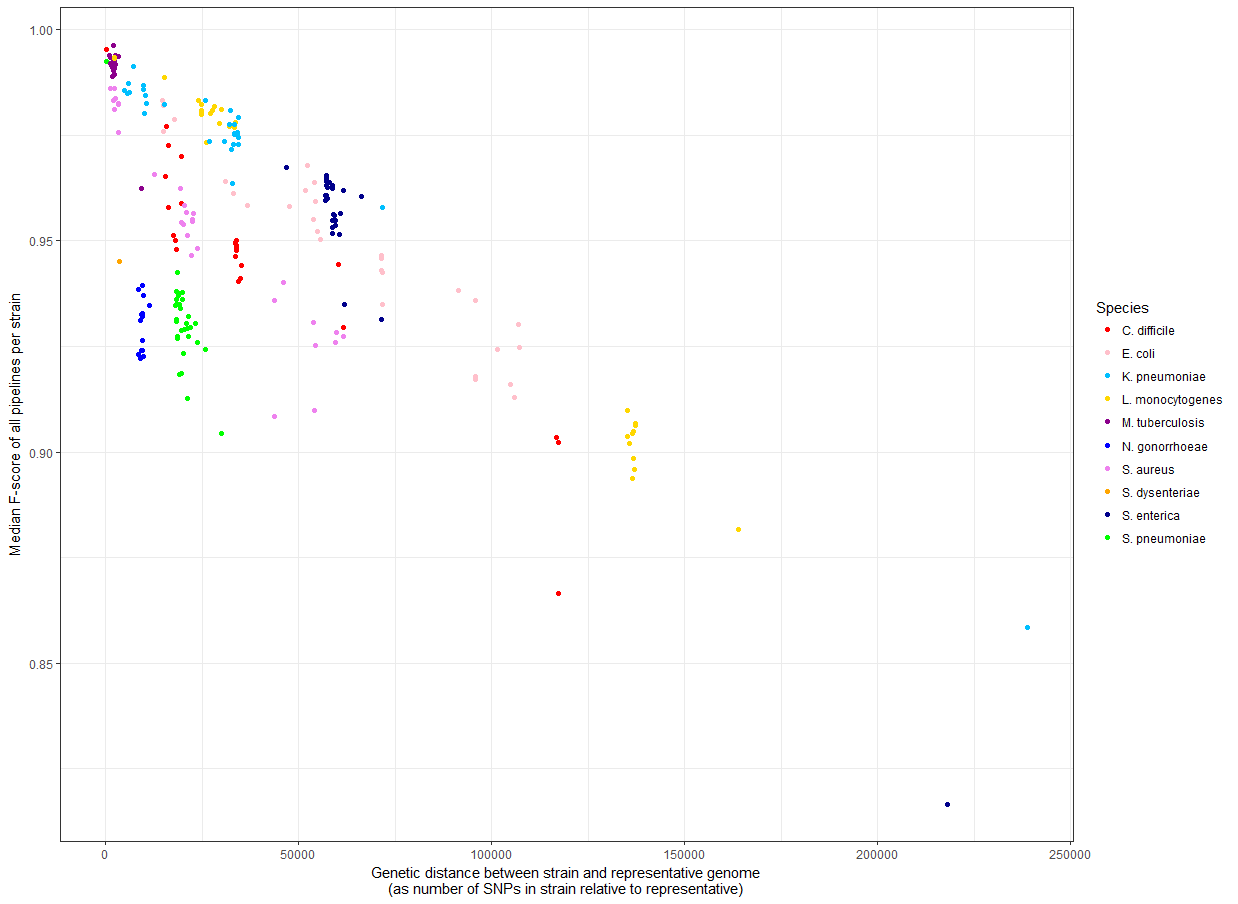

Supplement: giaa007_Supplement_Files [file giaa007_supplement_files.zip › Supplementary Figure 1.png]

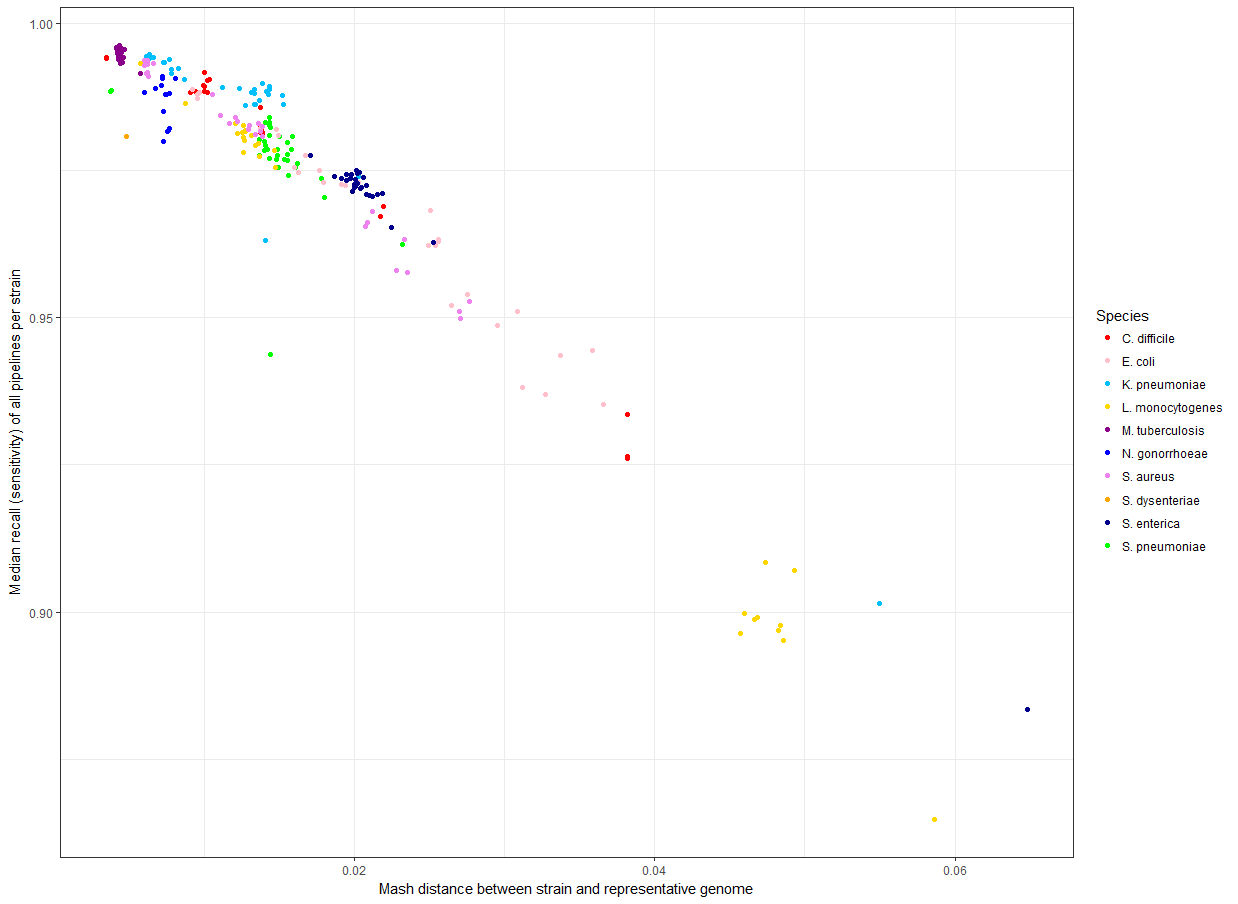

Supplement: giaa007_Supplement_Files [file giaa007_supplement_files.zip › Supplementary Figure 2.png]

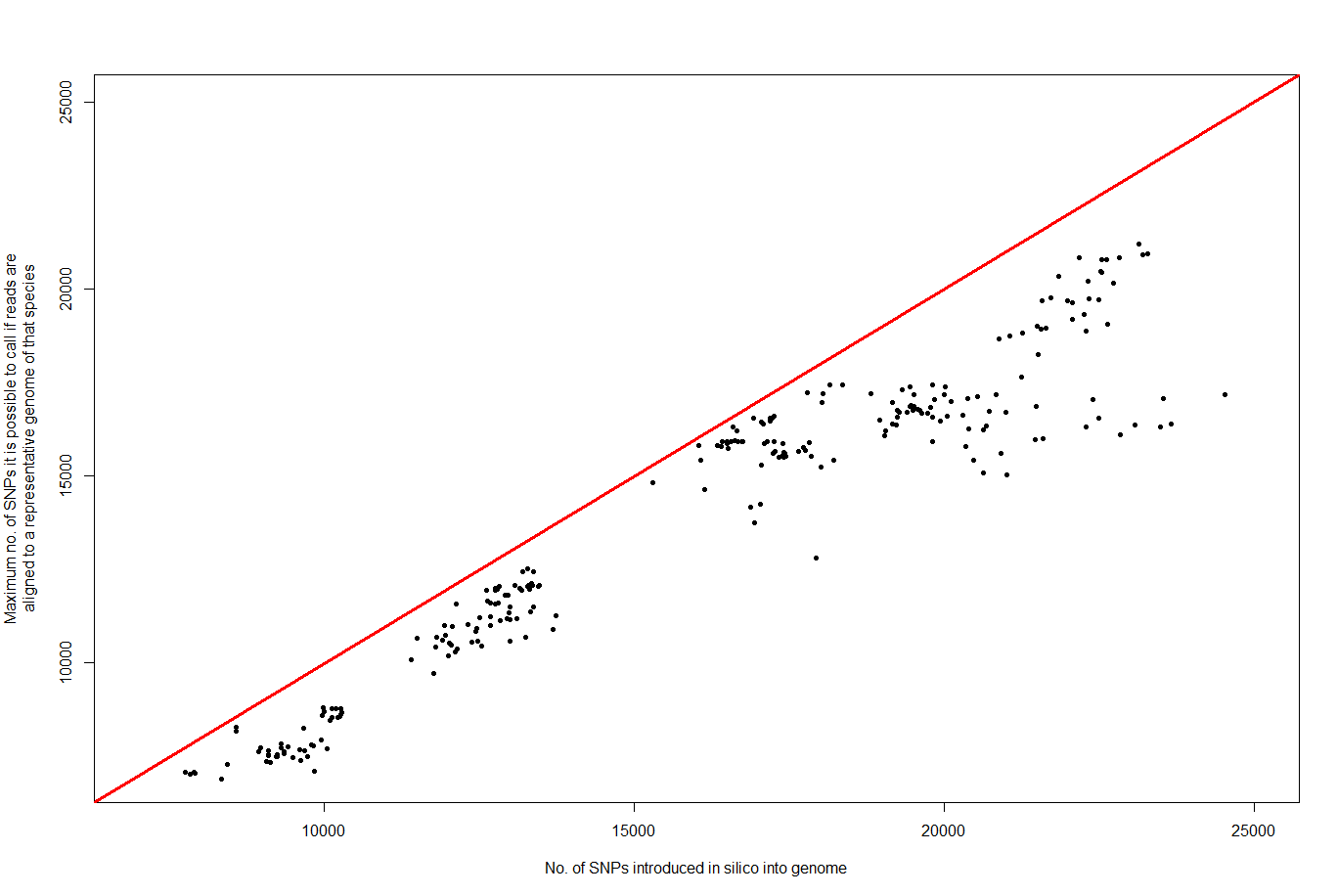

Supplement: giaa007_Supplement_Files [file giaa007_supplement_files.zip › Supplementary Figure 3.png]

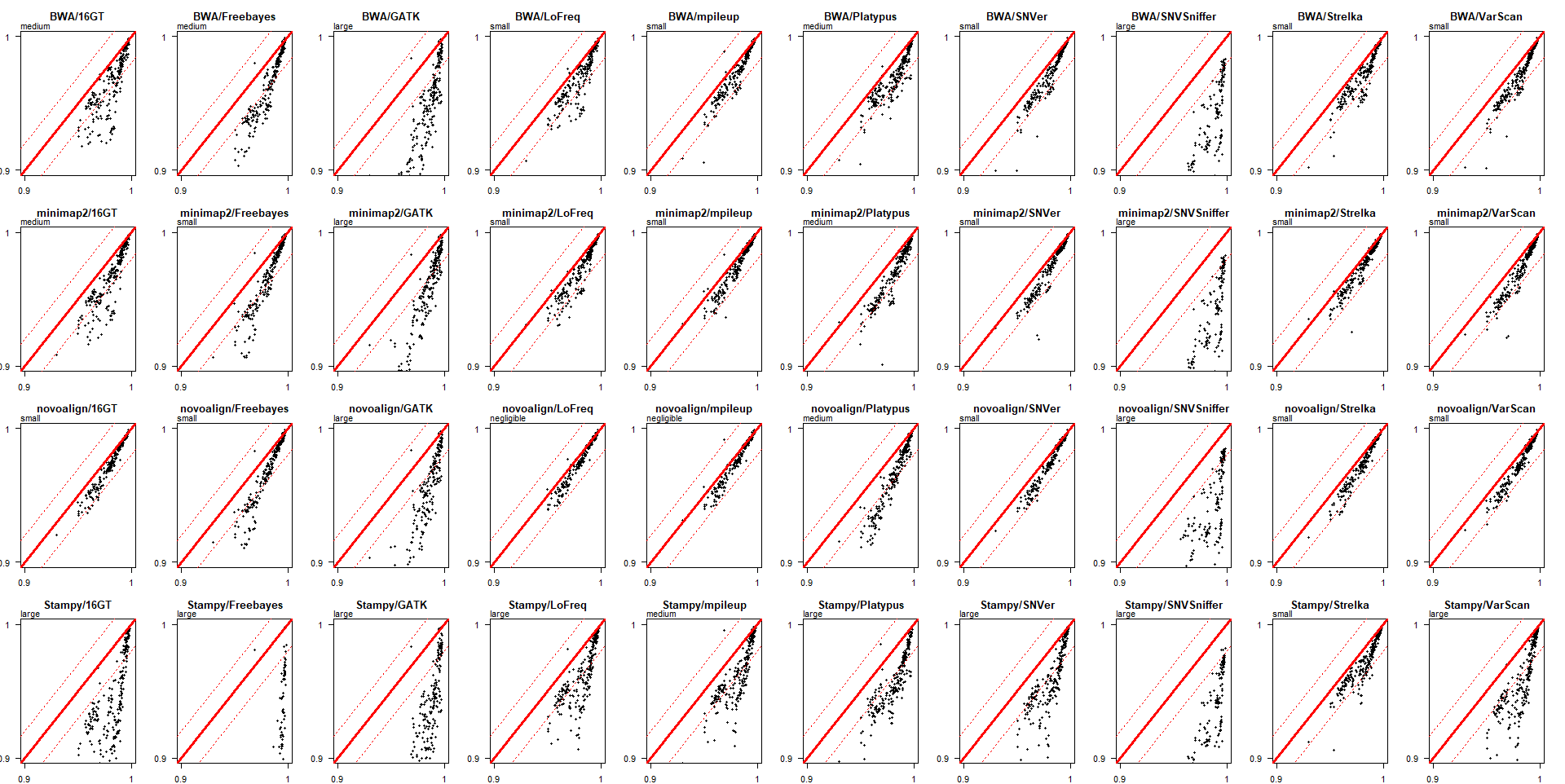

Supplement: giaa007_Supplement_Files [file giaa007_supplement_files.zip › Supplementary Figure 4.png]

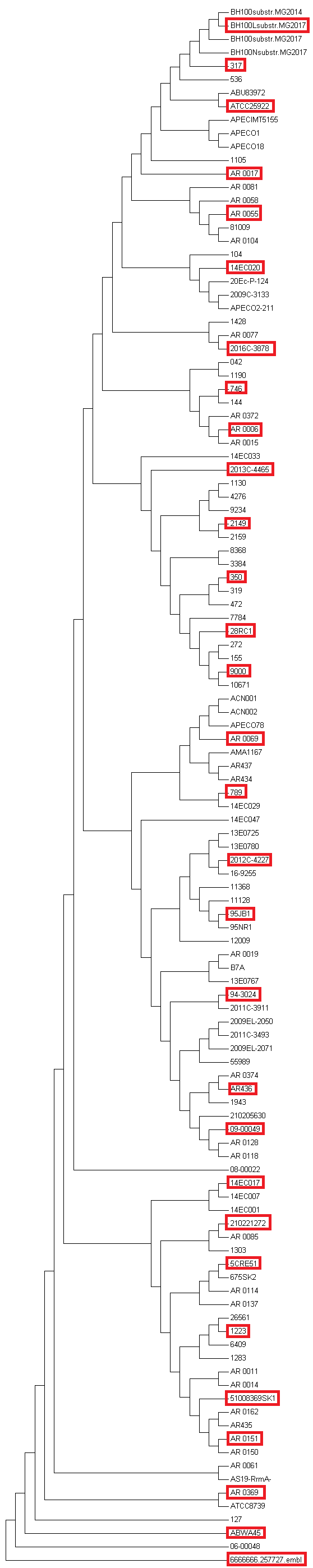

Supplement: giaa007_Supplement_Files [file giaa007_supplement_files.zip › Supplementary Figure 5.png]

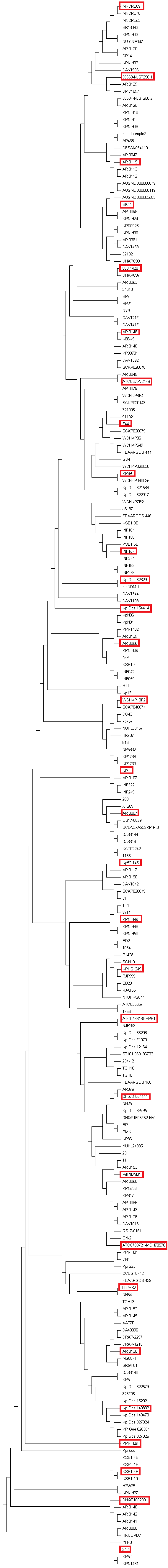

Supplement: giaa007_Supplement_Files [file giaa007_supplement_files.zip › Supplementary Figure 6.png]

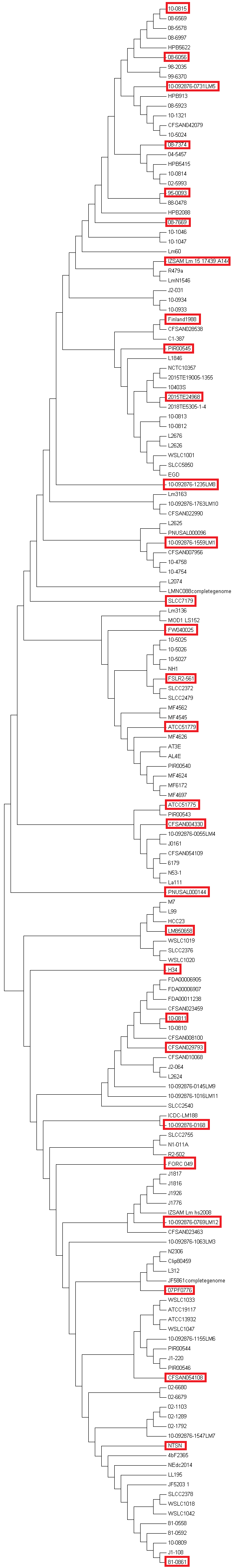

Supplement: giaa007_Supplement_Files [file giaa007_supplement_files.zip › Supplementary Figure 7.png]

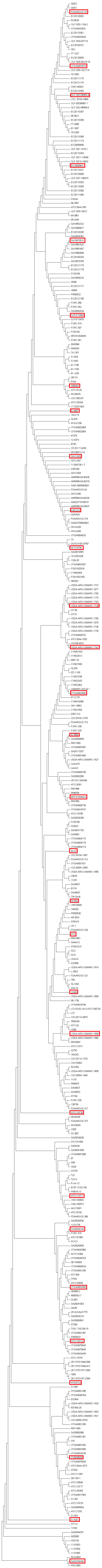

Supplement: giaa007_Supplement_Files [file giaa007_supplement_files.zip › Supplementary Figure 8.png]

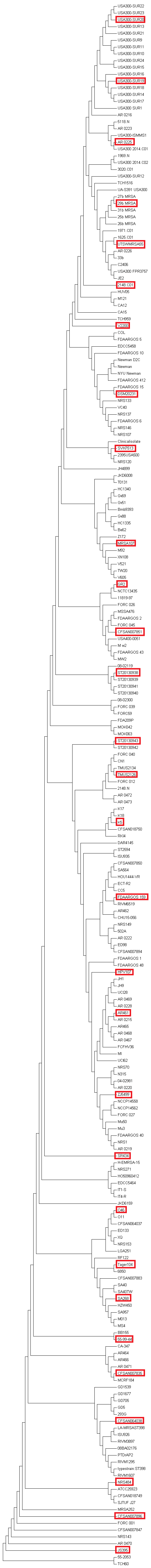

Supplement: giaa007_Supplement_Files [file giaa007_supplement_files.zip › Supplementary Figure 9.png]
