# Supplementary material for: Genomic diversity affects the accuracy of bacterial single-nucleotide polymorphism–calling pipelines
Source: Gigascience. 2020 Feb 6;9(2):giaa007. doi: 10.1093/gigascience/giaa007 (PMC7002876; doi:10.1093/gigascience/giaa007)
Supplement: giaa007_Response_to_Reviewer_Comments_Original_Submission [file giaa007_response_to_reviewer_comments_original_submission.pdf]

Author's Response To Reviewer Comments

Close

We would like to thank the reviewers for their comments, which we address in detail below. All line numbers refer to the unmarked version of the revised text.
Aside from in-text clarifications, the principal changes to this revised version are:
(a) A substantial expansion of the supplementary material to include an archive comprising original scripts plus raw materials (that is, reference genomes, associated indices and truth sets) and output (that is, VCFs), allowing replication and expansion of the evaluation employing real data. This is now available as Supplementary Dataset 2, at https://ora.ox.ac.uk/objects/uuid:8f902497-955e-4b84-9b85-693ee0e4433e (an archive of the simulated datasets was already made available in the original manuscript as Supplementary Dataset 1, at http://dx.doi.org/10.5287/bodleian:AmNXrjYN8).
(b) An expansion of the number of aligner/caller combinations evaluated (on real data) from 41 to 209, with associated updates of supplementary tables 9 and 10, and one additional figure (figure 7). These additional pipelines also now include the 'all-in-one' SpeedSeq and SPANDx.
(c) An expansion of the supplementary text to include more detailed justifications for various choices, such as not repeat-masking the reference genome and for simulating reads at high depth.

Reviewer reports:

Reviewer #1: This paper presents the results of analyzing several datasets with a range of short read aligners and variant callers. The analysis is exhaustive and the results are important for researchers conducting these type of analyses, especially when using a single reference genome.

The results seem to confirm results seen by others, specifically Bertels et al. (PMID:24600054) and Sahl et al. (PMID:28348869), neither of which are cited. The RealPhy paper suggests using multiple reference genomes and merging the results to mitigate the effects of a distant reference.

Response: We have expanded the text to discuss other approaches to overcoming issues that arise when using a single reference genome, and have added the two references suggested by the reviewer. Specifically, we have added, from line 516, the text:
"An alternative approach to reducing errors introduced when using a single reference genome could be to merge results from multiple reference genomes (the approach taken by REALPHY to reconstruct phylogenies from bacterial SNPs [98]) or from multiple aligners and/or callers, obtaining consensus calls across a set of methods. This is the approach taken by the NASP pipeline [99], which can integrate data from any combination of the aligners Bowtie2, BWA-mem, Novoalign and SNAP, and the callers GATK UnifiedGenotyper, mpileup, SolSNP and VarScan (ensemble approaches have similarly been used for somatic variant calling, for example by SomaticSeq [100])."

The goal of the paper is to analyze 'SNP pipelines', although only a single 'self contained' SNP pipeline (Snippy) is included. I would argue that the rest of the analyses are based on aligner/variant caller pairs and not complete SNP pipelines. While this could be a semantic issue, comparing Snippy with these other methods could be considered an apples to oranges comparison. Out of the dozens of 'self contained' pipelines, why was only Snippy used? The fact that Snippy is performing much better than its corresponding aligner/variant caller pairs suggests that it is doing additional work not performed by other 'pipelines'.

Response: We had used 'pipeline' as shorthand for 'aligner/caller combination', but we agree they are not synonymous. To that end, we now state early in the introduction (line 87) that:

"SNP calling pipelines are typically constructed around a read aligner (which takes FASTQ as input and produces BAM as output) and a variant caller (which takes BAM as input and produces VCF as output), often with several pre- and post-processing steps (for instance, cleaning a raw FASTQ prior to alignment, or filtering a BAM prior to variant calling). For the purpose of this study, when evaluating the two core components of aligner and caller, we use 'pipeline' to mean 'an aligner/caller combination, with all other steps in common'."

Further to the description of each aligner and caller used in this study, we now also note (line 106) that: "where possible, we applied a common set of pre- or post-processing steps to each aligner/caller combination, although note that these could differ from those applied within an 'all-in-one' tool (discussed further in Supplementary Text 1)."

The advantage to users (especially less experienced users) of having "all-in-one/self-contained" SNP analysis pipelines is clear, however, in that they potentially substantially streamline bioinformatics workflows; we therefore believe that they are useful to include in our study. We have now expanded the evaluation to contain two other 'all-in-one' pipelines, SpeedSeq and SPANDx, and discuss in the supplementary text (line 719) why some others could not reasonably be used – in certain cases, because they offer the user a choice of aligner and/or caller (such as PHEnix) and so cannot be easily be evaluated as a single entity. Specifically in line 436 of the main text, we have added: "in this study we sought to use all aligners and callers uniformly, with equivalent quality-control steps applied to all reads. To that end, while direct comparisons of any aligner/caller pipeline with 'all-in-one' tools (such as Snippy, SPANDx and SpeedSeq) are possible, the results should be interpreted with caution. This is because it is in principle possible to improve the performance of the former through additional quality control steps – that is, compared to an 'all-in-one' tool, it is not necessarily the aligner or caller alone to which any difference in performance may be attributed. For instance, although Snippy and SpeedSeq employ BWA-mem and Freebayes, both tools are distinct from the BWA-mem/Freebayes pipeline used in this study (Figure 7 and Supplementary Table 10). This is because they implement additional steps between the BWA and Freebayes components, as well as altering the default parameters relative to standalone use. Snippy, for example, employs samclip (https://github.com/tseemann/samclip) to post-process the BAM file produced by BWA-mem, removing clipped alignments in order to reduce false positive SNPs near structural variants".

For introduced SNPs, it would be nice to know which SNPs are in paralogs and tandem repeats. These regions could be problematic and may be introducing false positives due to mismapping. While the authors discuss that using long reads could fix some of these problems, the effects of including these regions on the results should be considered. For example, the true positive SNPs in the real data analyses are based on MUMmer and Parsnp, neither of which filter paralogous regions. The nature of the alignment algorithm would likely control how many false SNPs were reported in these regions and could impact overall performance.

Response: We agree that the retention of paralogous regions would likely increase the rate of read mis-mapping and thereby the number of false positive calls, although assuming this to be a systematic error, it should not affect the rank order of pipelines. In the 'study limitations' section of the discussion, we have added this point to the main text (line 365): "For the strain-to-representative genome alignments in this study, we considered SNP calls only within one-to-one alignment blocks and cannot exclude the possibility that repetitive or highly mutable regions within these blocks have been misaligned. However, we did not seek to identify and exclude SNPs from these regions as, even if present, this would have a systematic negative effect on the performance of each pipeline. To demonstrate this, we re-calculated each performance metric for the 209 pipelines evaluated using real sequencing data after identifying, and masking, repetitive regions of the reference genome with self-self BLASTn (as in [77]). As we already required reference bases within each one-to-one alignment block to be supported by both nucmer and ParSnp calls (that is, implicitly masking ambiguous bases), we found that repeat-masking the reference genome had negligible effect on overall F-score although marginally improved precision (see Supplementary Text 1)."

Within Supplementary Text 1, we added the following text at line 662:
"To demonstrate the effect of additional repeat-masking, we re-calculated precision, recall and F-score for each of the 209 pipelines evaluated using real sequencing data (i.e., when aligning 18 sets of non-simulated reads against one of the six representative Gram-negative genomes detailed in Supplementary Table 8). We did not test the effect of repeat-masking using the simulated E. coli datasets (as above) because this represents only one reference genome (i.e., E. coli K-12 substr. MG1655). Repetitive regions in each genome were first identified by self-self BLASTn (as in [78]), using BLAST+ v2.7.1 with default parameters, and considered those with alignments of ≥ 95% identity over length ≥ 100bp, with no more than 1 gap, and an E-value < 0.05 (not including the match of the entire genome against itself)." We also illustrate the effect of additional masking on the F-score, precision and recall distributions with a new figure within Supplementary Text 1 (on page 33).

Some discussion on how these effects could impact data interpretation would be helpful. In the case of

transmission events, one would assume that a closely related reference would be chosen, which would mitigate biases, any may not be sensitive to the aligner/caller used. How would these results affect large, population genomics studies?

Response: We agree that this is a useful point to include, but would note that many transmission studies use a single reference so that when mapping all isolates (i.e. both putative outbreak and non-outbreak isolates), the reference is typically most similar to the outbreak isolates of interest, or is chosen because a particular genome has widespread prior use in similar evaluations. We have added to the discussion (line 478):
"More closely related genomes would have lower Mash distances and so be more suitable as reference genomes for SNP calling. This would be particularly appropriate if, for example, studying transmission events as a closely-related reference would increase specificity, irrespective of the aligner or caller used. For larger studies that require multiple samples to be processed using a common reference, the choice of reference genome could be one which 'triangulates' between the set of samples – that is, has on average a similar distance to each sample, rather than being closer to some and more distant from others."

Reviewer #2: In this paper, Bush et al. evaluate a large number of bacterial SNP calling pipelines against variously divergent references. Their main conclusion is that different pipelines perform very differently as the reference diverges, and that Jaccard similarity is a good way to choose the "best" (closest) reference for mapping.

This paper is full of nice figures and analyses, and moreover we have seen the same thing in our work, so I agreed with the major points of the paper in advance!

The only real weakness I see in the paper is that the authors use simulated data, which comes with many advantages but also means that oddball sequencer mistakes are not necessarily measured. This is an acceptable tradeoff to me, but I wanted to mention it...

Response: We initially used simulated data from 10 species, although the latter half of the results section employed real data from 16 environmentally-sourced samples plus 2 reference strains (detailed from line 730 onwards and made available as Supplementary Dataset 2). The "real-world" isolates used are members of the Enterobacteriaceae bacterial family, and are typically genetically complex (i.e. having multiple orthologs/paralogs, repeats etc), thus representing, in our minds, an appropriate analytical challenge.

I think the general conclusion that Jaccard similarity (or, really, ANI) is the best way to choose reference genomes is both important and indisputable, so it's nice to see a thorough evaluation of it.

I encourage the authors to make their evaluation code, scripts, notebooks, figure generation, etc. available. I could not seem to find it. Reproducibility is minimal but acceptable given Supp Text 1.

Response: We agree that reproducibility is critical to benchmarking studies and to that end have supplemented the pseudocode of Supplementary Text 1 by:
(a) Making the full set of evaluation and figure creation scripts available as a public archive, Supplementary Dataset 2 (https://ora.ox.ac.uk/objects/uuid:8f902497-955e-4b84-9b85-693ee0e4433e). This archive also contains both the raw data necessary for evaluation (i.e. reads and indexed reference genomes) alongside example output (i.e. VCFs and summary tables).
(b) Adding an additional 'operating notes' section to Supplementary Text 1, detailing our specific experience with certain tools, with particular regard to bugs and workarounds. This section may be considered a 'laboratory notebook'.

Close
