# Supplementary material for: Genomic diversity affects the accuracy of bacterial single-nucleotide polymorphism–calling pipelines
Source: Gigascience. 2020 Feb 6;9(2):giaa007. doi: 10.1093/gigascience/giaa007 (PMC7002876; doi:10.1093/gigascience/giaa007)
Supplement: giaa007_Response_to_Reviewer_Comments_Revision_1 [file giaa007_response_to_reviewer_comments_revision_1.pdf]

Author's Response To Reviewer Comments

Close

Response to reviewers

For the Perl scripts we would recommend to put these in a code repository and include a software section at the end of the paper that is structured as follows:

Availability of supporting source code and requirements
Project name: e.g. My bioinformatics project
Project home page: e.g. https://github.com/ISA-tools
Operating system(s): e.g. Platform independent
Programming language: e.g. Java
Other requirements: e.g. Java 1.3.1 or higher, Tomcat 4.0 or higher
License: e.g. GNU GPL, FreeBSD etc.
RRID: if applicable, e.g. RRID: SCR_014986 (see below)

Response: we have added the Perl scripts to a GitHub repository, https://github.com/oxfordmmm/GenomicDiversityPaper, now referred to on line 1130. Lines 1133-1141 specify the requirements:
Project name: “Genomic diversity affects the accuracy of bacterial SNP calling pipelines”
Project home page: https://github.com/oxfordmmm/GenomicDiversityPaper
Operating system(s): platform-independent
Programming language: Perl (v5.22.1)
Other requirements: third-party software prerequisites are detailed in documentation provided with Supplementary Dataset 2 (https://ora.ox.ac.uk/objects/uuid:8f902497-955e-4b84-9b85-693ee0e4433e).
License: GNU GPL.

Reviewer reports:
Reviewer #1: The authors did a good job at addressing my previous comments as well as expanding the analyses to cover a more diverse suite of tools. The authors still use 'pipeline' to sometimes describe an aligner/variant caller and also an all-in-one method, which may cause confusion, but is ultimately their decision. The authors still mention Snippy as one of the best performing tools, which seems odd considering the performance in Supplementary Table 10 using real data. Perhaps the authors could state that snippy did well on simulated data, while other tools performed better on real data. The captions on the supplementary tables could also be updated to differentiate between simulated and real data.

Response: we removed from the abstract (line 47) the statement that “across the full range of genomes, among the consistently highest performing pipelines was Snippy” as this conclusion was drawn from its performance across both simulated and real datasets, when n=41 pipelines. However, with the expansion of the number of pipelines to 209, and the testing of these additional pipelines only on real data, we sought to keep the conclusions drawn based on real data distinct from those based on simulated data. To that end, we also amended line 549 to read “Nevertheless, Snippy, which employs Freebayes, is particularly robust to this, being among the most sensitive pipelines when evaluated using simulated data (Figure 5 and Supplementary Figure 4).” We have also amended the titles of Figure 5 and Supplementary Figure 4, and Supplementary Tables 3, 4, 6, 7, 13, 14, 15, 16 and 17 to emphasise their use of simulated data (the supplementary tables containing results from real data, numbers 9 and 10, were already so labelled).

Additionally, the authors include an analysis that masks repeats using BLAST. However, the thresholds chosen for BLAST will likely only mask very similar paralogs, while the more divergent paralogs are expected to have a greater impact on mis-mapping and variant discovery (this could just be a discussion point).

Response: we agree that the parameters used for repeat-masking are especially important and have

expanded the discussion to include this. We have added, at line 377: "it is important to note that the parameters used for repeat-masking will determine which paralogues will be successfully masked. For the purpose of this study, we used reasonably conservative parameters (detailed in Supplementary Text 1) and so expect to have primarily masked more similar paralogues. The likelihood of mis-mapping (and thereby false positive SNP calling) would increase among more divergent paralogues, although optimising parameters to detect these is non-trivial. More lenient repeat-masking parameters, in masking more divergent positions, would also reduce the number of true SNPs it is possible to call." This has also been added to the supplementary text, at lines 680-686.

Some additional thoughts that may improve the manuscript:

L306: The authors should mention that they also now include 2 additional "all-in-one" pipelines

Response: we have revised the sentence to read "we next expanded the scope of the evaluation to 209 pipelines (representing the addition of 12 aligners, 4 callers, and 2 'all-in-one' pipelines, SpeedSeq and SPANDx)…"

L1127-1128: Please check this link. I received a 404 error when I tried to access it. The link in the response to reviewers did work for me

Response: I'm afraid we can't replicate this 404 – we've re-checked the link (https://ora.ox.ac.uk/objects/uuid:8f902497-955e-4b84-9b85-693ee0e4433e) and do find it accessible.

Figure 7: The x-axis labels don't line up with the bars, which makes it difficult to interpret. Would staggering the labels between the top and bottom of the graph help with this?

Response: we have re-drawn with Figure 7 with better-positioned x-axis labels.

Close
