# Supplementary material for: Genomic diversity affects the accuracy of bacterial single-nucleotide polymorphism–calling pipelines
Source: Gigascience. 2020 Feb 6;9(2):giaa007. doi: 10.1093/gigascience/giaa007 (PMC7002876; doi:10.1093/gigascience/giaa007)
Supplement: giaa007_Reviewer_1_Report_Original_Submission — Jason Sahl -- 7/15/2019 Reviewed [file giaa007_reviewer_1_report_original_submission.pdf]

Reviewer Report

Title: Genomic diversity affects the accuracy of bacterial SNP calling pipelines

Version: Original Submission **Date:** 7/15/2019

Reviewer name: Jason Sahl

Reviewer Comments to Author:

This paper presents the results of analyzing several datasets with a range of short read aligners and variant callers. The analysis is exhaustive and the results are important for researchers conducting these type of analyses, especially when using a single reference genome. The results seem to confirm results seen by others, specifically Bertels et al. (PMID:24600054) and Sahl et al. (PMID:28348869), neither of which are cited. The RealPhy paper suggests using multiple reference genomes and merging the results to mitigate the effects of a distant reference.

The goal of the paper is to analyze 'SNP pipelines', although only a single 'self contained' SNP pipeline (Snippy) is included. I would argue that the rest of the analyses are based on aligner/variant caller pairs and not complete SNP pipelines. While this could be a semantic issue, comparing Snippy with these other methods could be considered an apples to oranges comparison. Out of the dozens of 'self contained' pipelines, why was only Snippy used? The fact that Snippy is performing much better than its corresponding aligner/variant caller pairs suggests that it is doing additional work not performed by other 'pipelines'.

For introduced SNPs, it would be nice to know which SNPs are in paralogs and tandem repeats. These regions could be problematic and may be introducing false positives due to mismapping. While the authors discuss that using long reads could fix some of these problems, the effects of including these regions on the results should be considered. For example, the true positive SNPs in the real data analyses are based on MUMmer and Parsnp, neither of which filter paralogous regions. The nature of the alignment algorithm would likely control how many false SNPs were reported in these regions and could impact overall performance.

Some discussion on how these effects could impact data interpretation would be helpful. In the case of transmission events, one would assume that a closely related reference would be chosen, which would mitigate biases, any may not be sensitive to the aligner/caller used. How would these results affect large, population genomics studies?

Methods

Are the methods appropriate to the aims of the study, are they well described, and are necessary controls included? Choose an item.

Conclusions

Are the conclusions adequately supported by the data shown? Choose an item.

Reporting Standards

Does the manuscript adhere to the journal's guidelines on minimum standards of reporting? Choose an item.

Choose an item.

Statistics

Are you able to assess all statistics in the manuscript, including the appropriateness of statistical tests used? Choose an item.

Quality of Written English

Please indicate the quality of language in the manuscript: Choose an item.

Declaration of Competing Interests

Please complete a declaration of competing interests, considering the following questions:

- Have you in the past five years received reimbursements, fees, funding, or salary from an organisation that may in any way gain or lose financially from the publication of this manuscript, either now or in the future?
- Do you hold any stocks or shares in an organisation that may in any way gain or lose financially from the publication of this manuscript, either now or in the future?
- Do you hold or are you currently applying for any patents relating to the content of the manuscript?
- Have you received reimbursements, fees, funding, or salary from an organization that holds or has applied for patents relating to the content of the manuscript?
- Do you have any other financial competing interests?
- Do you have any non-financial competing interests in relation to this paper?

If you can answer no to all of the above, write 'I declare that I have no competing interests' below. If your reply is yes to any, please give details below.

I declare that I have no competing interests

I agree to the open peer review policy of the journal. I understand that my name will be included on my report to the authors and, if the manuscript is accepted for publication, my named report including any attachments I upload will be posted on the website along with the authors' responses. I agree for my report to be made available under an Open Access Creative Commons CC-BY license (http://creativecommons.org/licenses/by/4.0/). I understand that any comments which I do not wish to be included in my named report can be included as confidential comments to the editors, which will not be published.

Choose an item.

To further support our reviewers, we have joined with Publons, where you can gain additional credit to further highlight your hard work (see: https://publons.com/journal/530/gigascience). On publication of this paper, your review will be automatically added to Publons, you can then choose whether or not to claim your Publons credit. I understand this statement.

Yes Choose an item.
