# Supplementary material for: Genomic diversity affects the accuracy of bacterial single-nucleotide polymorphism–calling pipelines
Source: Gigascience. 2020 Feb 6;9(2):giaa007. doi: 10.1093/gigascience/giaa007 (PMC7002876; doi:10.1093/gigascience/giaa007)
Supplement: giaa007_Reviewer_1_Report_Revision_1 — Jason Sahl -- 12/17/2019 Reviewed [file giaa007_reviewer_1_report_revision_1.pdf]

Reviewer Report

Title: Genomic diversity affects the accuracy of bacterial SNP calling pipelines

Version: Revision 1 **Date:** 12/17/2019

Reviewer name: Jason Sahl

Reviewer Comments to Author:

The authors did a good job at addressing my previous comments as well as expanding the analyses to cover a more diverse suite of tools. The authors still use 'pipeline' to sometimes describe an aligner/variant caller and also an all-in-one method, which may cause confusion, but is ultimately their decision. The authors still mention Snippy as one of the best performing tools, which seems odd considering the performance in Supplementary Table 10 using real data. Perhaps the authors could state that snippy did well on simulated data, while other tools performed better on real data. The captions on the supplementary tables could also be updated to differentiate between simulated and real data. Additionally, the authors include an analysis that masks repeats using BLAST. However, the thresholds chosen for BLAST will likely only mask very similar paralogs, while the more divergent paralogs are expected to have a greater impact on mis-mapping and variant discovery (this could just be a discussion point). Some additional thoughts that may improve the manuscript:
L306: The authors should mention that they also now include 2 additional "all-in-one" pipelines
L1127-1128: Please check this link. I received a 404 error when I tried to access it. The link in the response to reviewers did work for me
Figure 7: The x-axis labels don't line up with the bars, which makes it difficult to interpret. Would staggering the labels between the top and bottom of the graph help with this?

Methods

Are the methods appropriate to the aims of the study, are they well described, and are necessary controls included? Choose an item.

Conclusions

Are the conclusions adequately supported by the data shown? Choose an item.

Reporting Standards

Does the manuscript adhere to the journal's guidelines on [minimum standards of reporting?](#) Choose an item.

Choose an item.

Statistics

Are you able to assess all statistics in the manuscript, including the appropriateness of statistical tests used? Choose an item.

Quality of Written English

Please indicate the quality of language in the manuscript: Choose an item.

Declaration of Competing Interests

Please complete a declaration of competing interests, considering the following questions:

- Have you in the past five years received reimbursements, fees, funding, or salary from an organisation that may in any way gain or lose financially from the publication of this manuscript, either now or in the future?
- Do you hold any stocks or shares in an organisation that may in any way gain or lose financially from the publication of this manuscript, either now or in the future?
- Do you hold or are you currently applying for any patents relating to the content of the manuscript?
- Have you received reimbursements, fees, funding, or salary from an organization that holds or has applied for patents relating to the content of the manuscript?
- Do you have any other financial competing interests?
- Do you have any non-financial competing interests in relation to this paper?

If you can answer no to all of the above, write 'I declare that I have no competing interests' below. If your reply is yes to any, please give details below.

I declare that I have no competing interests

I agree to the open peer review policy of the journal. I understand that my name will be included on my report to the authors and, if the manuscript is accepted for publication, my named report including any attachments I upload will be posted on the website along with the authors' responses. I agree for my report to be made available under an Open Access Creative Commons CC-BY license (http://creativecommons.org/licenses/by/4.0/). I understand that any comments which I do not wish to be included in my named report can be included as confidential comments to the editors, which will not be published.

Choose an item.

To further support our reviewers, we have joined with Publons, where you can gain additional credit to further highlight your hard work (see: https://publons.com/journal/530/gigascience). On publication of this paper, your review will be automatically added to Publons, you can then choose whether or not to claim your Publons credit. I understand this statement.

Yes Choose an item.
